# Supplementary material for: Longitudinal variability in the urinary microbiota of healthy premenopausal women and the relation to neighboring microbial communities: A pilot study
Source: PLoS One. 2022 Jan 14;17(1):e0262095. doi: 10.1371/journal.pone.0262095 (PMC8759677; doi:10.1371/journal.pone.0262095)
Supplement: S1 Fig — (PDF) [file pone.0262095.s001.pdf]

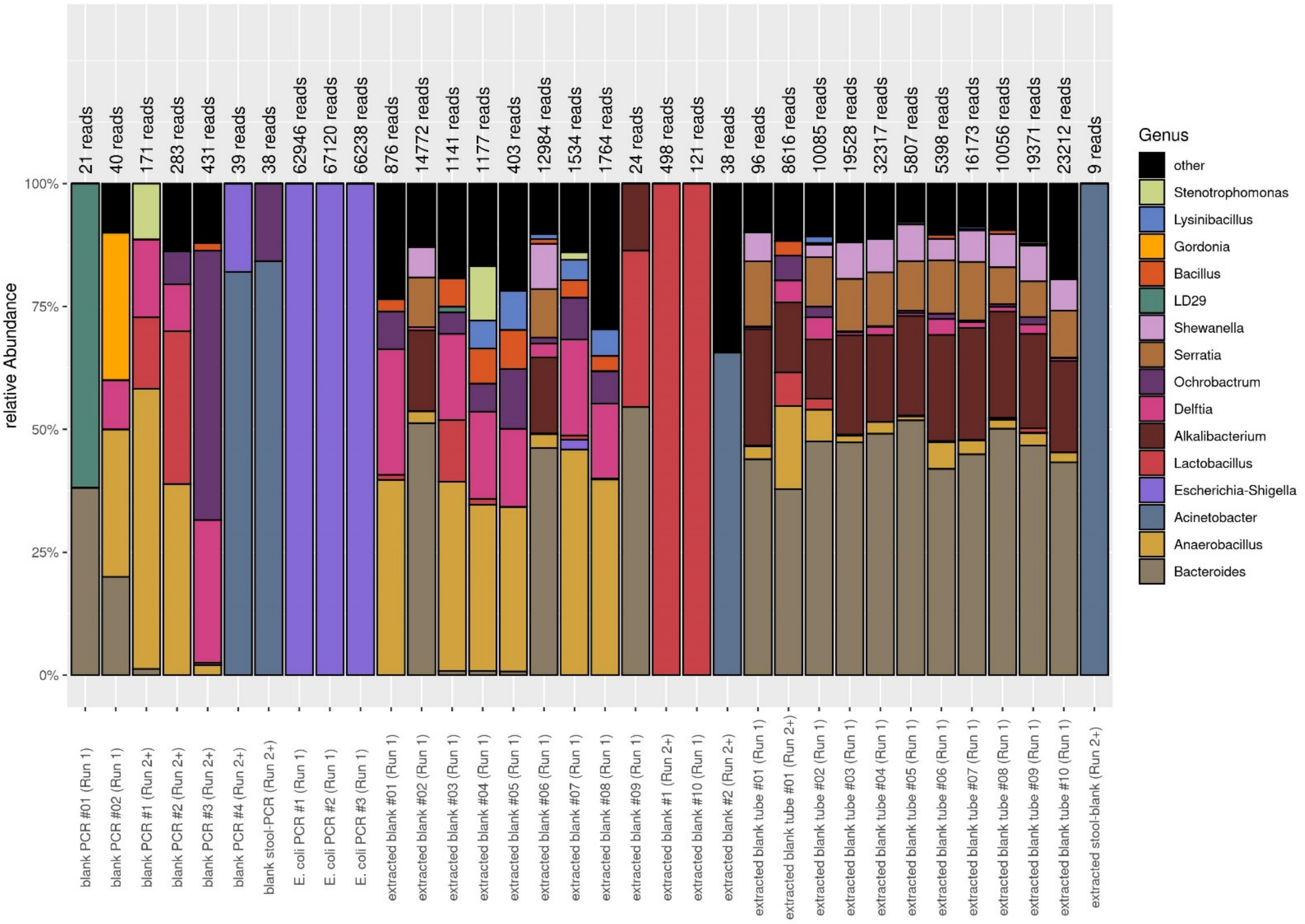

S1 Fig: Relative abundance ofbacterial genera and number oftotal readsincontrol samples

Negative control samples repeatedly showed high abundances of ASVs belonging to Anaerobacillus, Alkalibacterium, Bacteroides, Shewanella and Serratia.

<u>blank PCR:</u> negative control from extraction buffer subjected to PCR and sequencing; <u>E. coli PCR:</u> positive control with E. coli DNA in extraction buffer subjected to PCR and sequencing; <u>extracted blank:</u> negative control from extraction buffer subjected to DNA extraction, PCR and sequencing; <u>extracted blank tube:</u> negative control from sterile saline sampled through urinary catheters and tubes imitating preanalytics of catheter urine samples
