# Supplementary material for: Longitudinal variability in the urinary microbiota of healthy premenopausal women and the relation to neighboring microbial communities: A pilot study
Source: PLoS One. 2022 Jan 14;17(1):e0262095. doi: 10.1371/journal.pone.0262095 (PMC8759677; doi:10.1371/journal.pone.0262095)
Supplement: S2 Fig — (PDF) [file pone.0262095.s002.pdf]

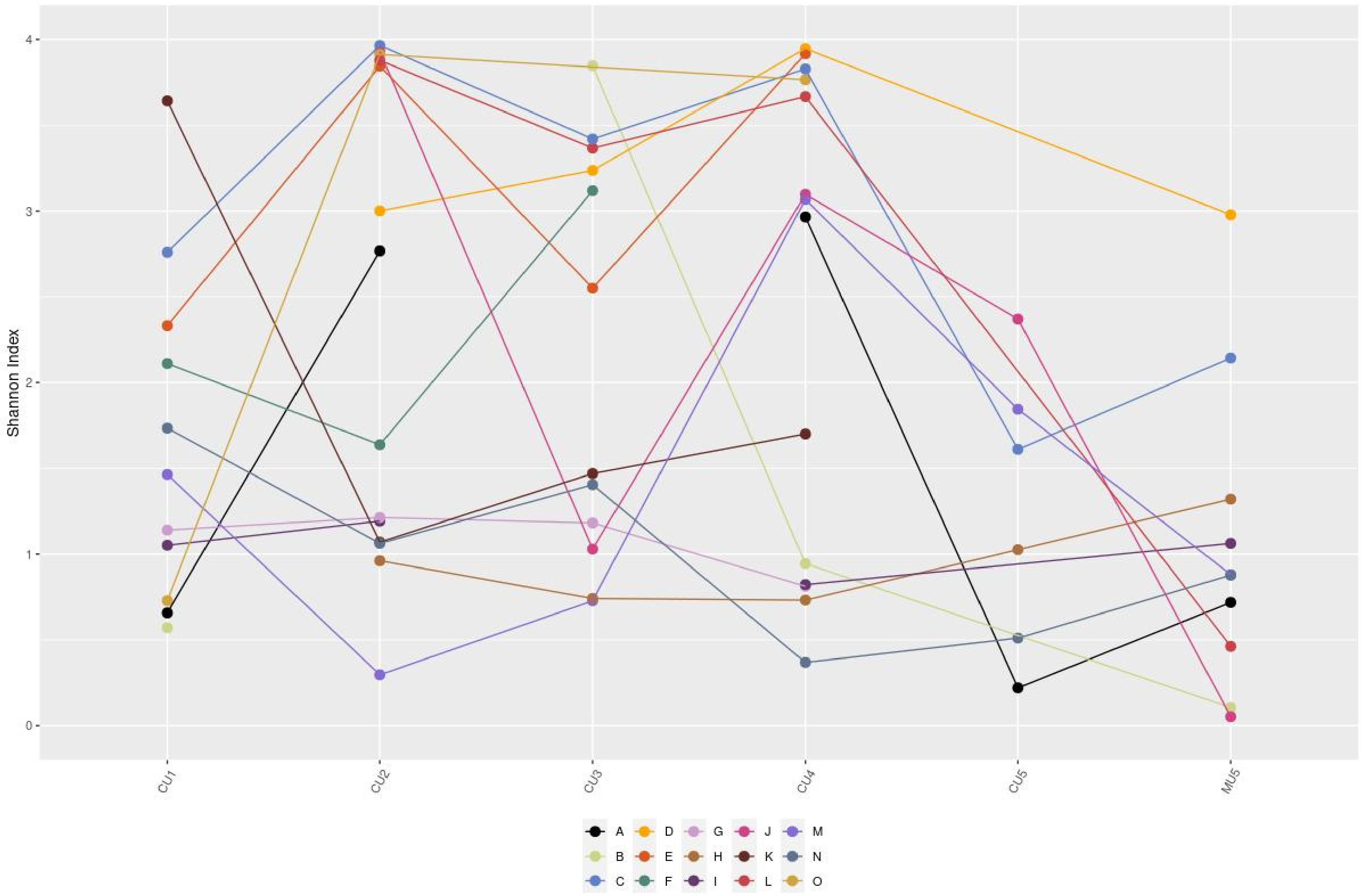

S2 Fig: Intraindividual changes in alpha diversity (Shannon diversity index) of urine samples collected during the study

Visit 1-4 took place within six months, visit 5 was additionally undertaken 1 year after visit 4 in 10 subjects with collection of both CU and MU at one visit.

CU: catheter urine; MU: midstream urine
