# Supplementary material for: Longitudinal variability in the urinary microbiota of healthy premenopausal women and the relation to neighboring microbial communities: A pilot study
Source: PLoS One. 2022 Jan 14;17(1):e0262095. doi: 10.1371/journal.pone.0262095 (PMC8759677; doi:10.1371/journal.pone.0262095)
Supplement: S3 Fig — (PDF) [file pone.0262095.s003.pdf]

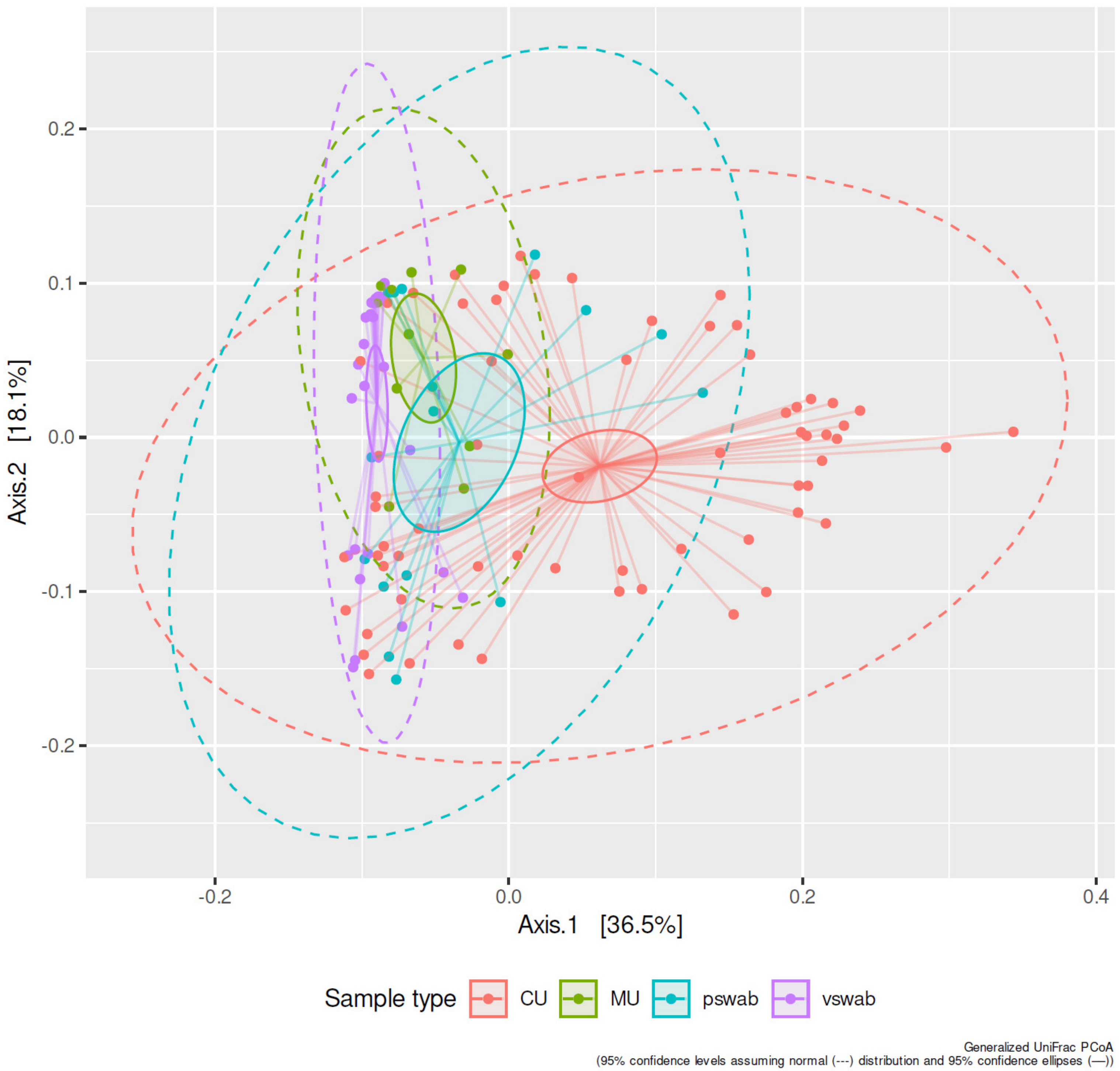

S3 Fig: Beta-diversity (generalized UniFrac) of CU, MU, pswab and vswab samples visualized by principal coordinates analysis (PCoA). Permutational multivariate analysis of variance using distance matrices (PERMANOVA) shows sample type as significant factor explaining observed beta-diversity partly ($R^2 = 0.17$, $p = 0.001$).

CU: catheter urine; MU: midstream urine; pswab: periurethral swab; vswab: vaginal swab
