# Supplementary material for: Longitudinal variability in the urinary microbiota of healthy premenopausal women and the relation to neighboring microbial communities: A pilot study
Source: PLoS One. 2022 Jan 14;17(1):e0262095. doi: 10.1371/journal.pone.0262095 (PMC8759677; doi:10.1371/journal.pone.0262095)
Supplement: S4 Fig — (PDF) [file pone.0262095.s004.pdf]

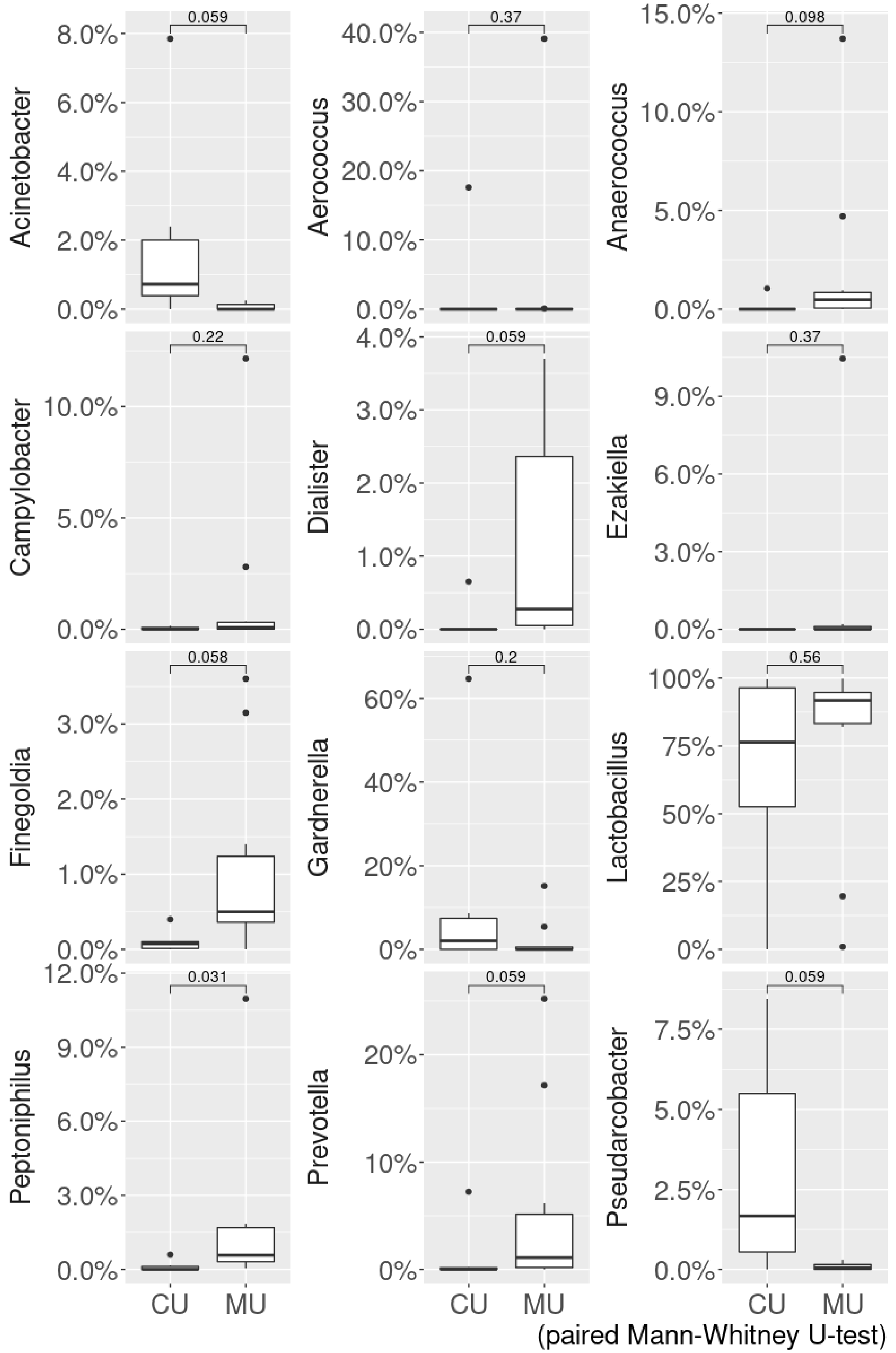

S4 Fig: Comparison of CU and MU samples. Mean relative abundances of genera detected in CU and MU samples from visit 5 with a minimum abundance of 1%. Reporting in the manuscript was restricted to those genera with a significant difference in mean relative abundances.
