# Supplementary material for: Longitudinal variability in the urinary microbiota of healthy premenopausal women and the relation to neighboring microbial communities: A pilot study
Source: PLoS One. 2022 Jan 14;17(1):e0262095. doi: 10.1371/journal.pone.0262095 (PMC8759677; doi:10.1371/journal.pone.0262095)
Supplement: S5 Fig — (PDF) [file pone.0262095.s005.pdf]

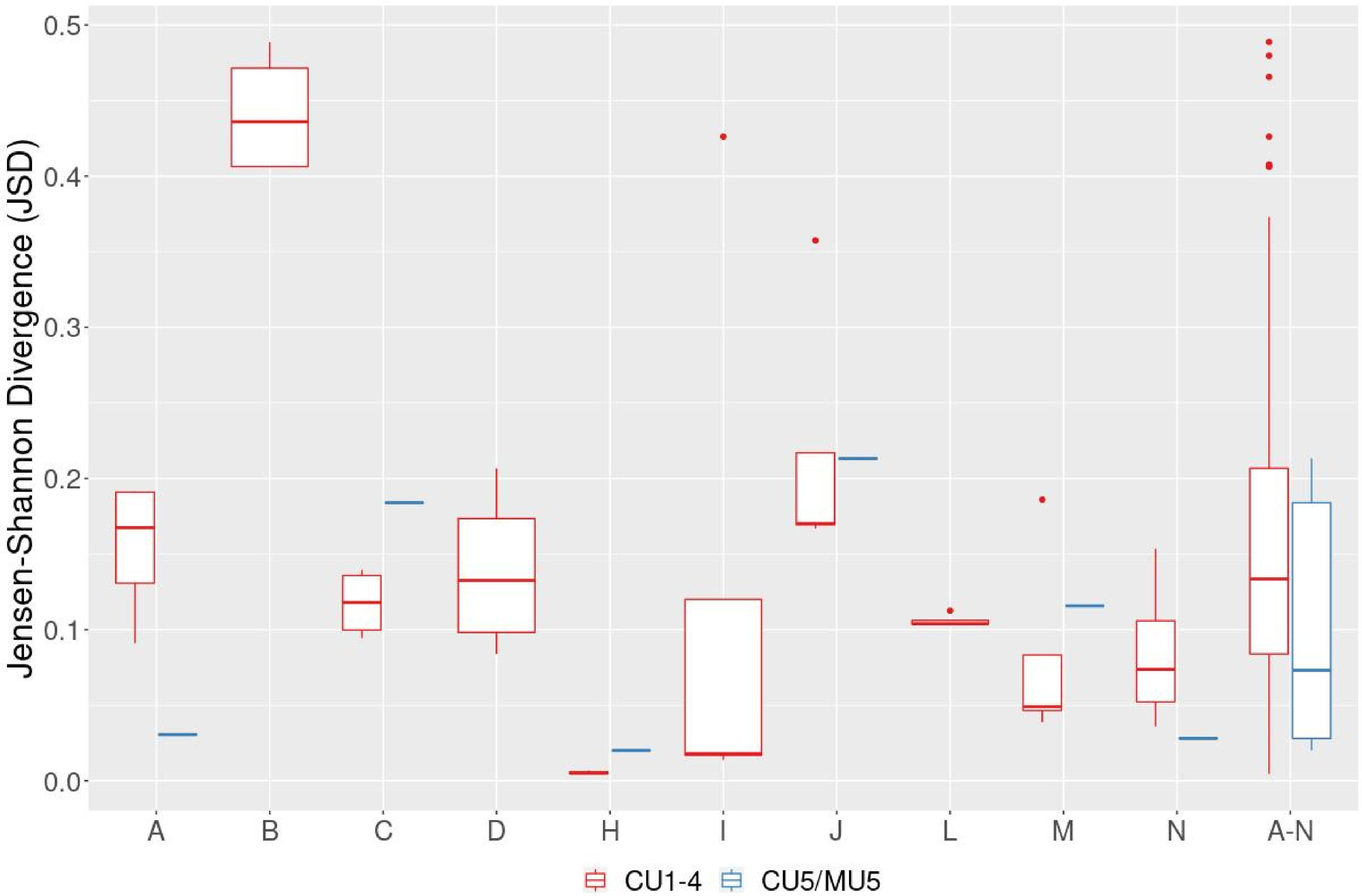

S5 Fig: Variability ofCUsamples and MUsamples shown asmedian Jensen-Shannon Divergence values Letters indicate volunteer. Red boxes represent JSD values from CU samples 1-4, blue dashes represent JSD values derived from comparison ofCUand corresponding MU sample from visit 5; CU: catheter urine; MU: midstream urine
