# Supplementary material for: Longitudinal variability in the urinary microbiota of healthy premenopausal women and the relation to neighboring microbial communities: A pilot study
Source: PLoS One. 2022 Jan 14;17(1):e0262095. doi: 10.1371/journal.pone.0262095 (PMC8759677; doi:10.1371/journal.pone.0262095)
Supplement: S6 Fig — (PDF) [file pone.0262095.s006.pdf]

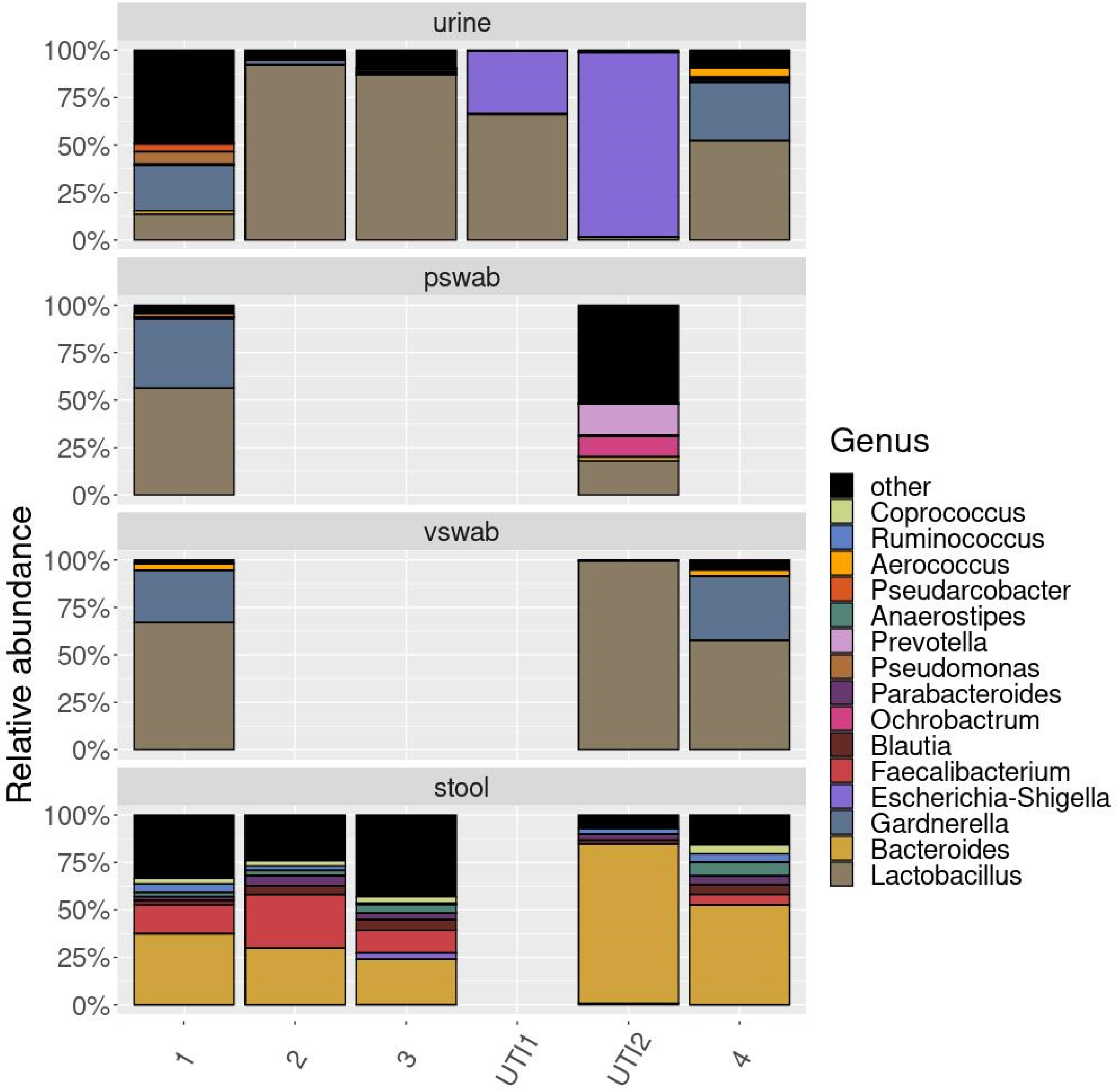

S6 Fig: Comparison of taxa abundance plots of all analyzed samples of volunteer K on genus level Volunteer K developed a urinary tract infection (UTI) between the third and fourth sampling visit leading to additional samples taken on the day of diagnosis (UTI1, midstream urine) and three days later (UTI2, catheter urine, pswab, vswab and stool). Urine samples 1-4 were catheter urine. Expanded culture of the urine from UTI1 yielded *Escherichia coli, Streptococcus agalactiae, Staphylococcus lugdunensis, Propionibacterium avidum* and *Actinomyces neuii.* Sequencing of urine samples from UTI1 and UTI2 showed a corresponding emergence of the genus *Escherichia-Shigella*.
pswab: periurethral swab, vswab: vaginal swab
