# Supplementary material for: Longitudinal variability in the urinary microbiota of healthy premenopausal women and the relation to neighboring microbial communities: A pilot study
Source: PLoS One. 2022 Jan 14;17(1):e0262095. doi: 10.1371/journal.pone.0262095 (PMC8759677; doi:10.1371/journal.pone.0262095)
Supplement: S1 Table — (PDF) [file pone.0262095.s007.pdf]

S1 Table: Fifteen most abundant taxa in different sample types on genus level

Sample type	Genus	Number of samples (%)*	Relative abundance in %				
			Mean	SD	Median	Min	Max
CU (n=63)	*Lactobacillus*	61 (98.8)	49.7	37.7	49.7	0.0	100.0
	Gardnerella	32 (50.8)	8.4	16.5	0.1	0.0	64.7
	Pseudarcobacter	60 (95.2)	3.7	3.7	2.2	0.0	12.1
	Pseudomonas	56 (88.9)	2.6	2.8	1.3	0.0	8.9
	Aeromonas	58 (92.0)	2.4	2.5	1.3	0.0	7.8
	Shewanella	57 (90.5)	1.7	1.8	1.0	0.0	6.6
	Bacteroides	53 (84.1)	1.5	1.9	0.6	0.0	8.5
	Vibrio	57 (90.5)	1.5	1.5	0.9	0.0	5.8
	Ochrobactrum	43 (85.3)	1.4	2.4	0.3	0.0	13.0
	Serratia	50 (79.4)	1.3	1.4	0.6	0.0	4.3
	Enterococcus	51 (81.0)	1.2	1.3	0.7	0.0	4.2
	Psychrobacter	55 (87.3)	1.2	1.2	0.9	0.0	4.3
	Chryseobacterium	55 (87.3)	1.1	1.2	0.5	0.0	5.5
	Rhodanobacter	50 (79.4)	1.0	1.1	0.5	0.0	3.4
	Paludibacterium	52 (82.5)	0.9	1.0	0.4	0.0	3.6
Vswab (n=30)	*Lactobacillus*	29 (96.7)	84.4	28.7	98.6	0.0	100.0
	Gardnerella	16 (53.3)	7.6	19.4	0.1	0.0	97.1
	Sneathia	4 (13.3)	1.9	7.3	0.0	0.0	30.5
	Atopobium	7 (23.3)	1.6	5.2	0.0	0.0	25.3
	Ureaplasma	14 (46.7)	1.0	2.1	0.0	0.0	6.3
	Aerococcus	6 (20.0)	0.5	1.8	0.0	0.0	8.9
	Bifidobacterium	2 (6.7)	0.4	2.1	0.0	0.0	11.6
	Prevotella	11 (36.7)	0.4	1.5	0.0	0.0	8.5
	Gemella	3 (10.0)	0.4	1.6	0.0	0.0	8.5
	Clostridium_sensu_stricto_1	4 (13.3)	0.4	1.3	0.0	0.0	6.4
	Finegoldia	16 (53.5)	0.3	0.8	0.1	0.0	4.1
	Streptococcus	6 (20.0)	0.3	1.2	0.0	0.0	6.5
	Anaerococcus	9 (30.0)	0.2	0.4	0.0	0.0	1.3
	Veillonella	3 (10.0)	0.1	0.7	0.0	0.0	3.9
	Dialister	7 (23.3)	0.1	0.3	0.0	0.0	1.5

Sample type	Genus	Number of samples (%)*	Relative abundance in %				
			Mean	SD	Median	Min	Max
Pswab (n=15)	*Lactobacillus*	15 (100.0)	69.1	34.5	85.7	0.8	98.2
	Gardnerella	7 (46.7)	13.4	27.7	0.0	0.0	96.8
	Atopobium	4 (26.7)	1.8	4.6	0.0	0.0	15.5
	Ochrobactrum	15 (100.0)	1.6	2.8	0.5	0.1	10.6
	Finegoldia	10 (67.7)	1.1	2.8	0.3	0.0	10.7
	Prevotella	13 (86.7)	1.0	2.1	0.3	0.0	8.2
	Delftia	14 (93.3)	0.8	1.0	0.5	0.0	3.2
	Sneathia	2 (13.3)	0.6	2.2	0.0	0.0	8.4
	Psychrobacter	12 (80.0)	0.5	1.0	0.1	0.0	3.9
	Aerococcus	3 (20.0)	0.5	1.3	0.0	0.0	5.1
	Anaerobacillus	9 (60.0)	0.5	0.8	0.1	0.0	2.5
	Streptococcus	5 (33.3)	0.4	1.4	0.0	0.0	5.6
	Flavobacterium	8 (53.3)	0.4	0.9	0.1	0.0	3.2
	uncultured	9 (60.0)	0.3	0.5	0.1	0.0	1.5
	Enterococcus	5 (33.3)	0.3	1.0	0.0	0.0	4.0
Fecal samples (n=59)	*Bacteroides*	59 (100.0)	21.7	12.4	22.4	2.3	54.0
	Faecalibacterium	58 (98.3)	15.4	7.7	13.7	0.0	35.2
	Blautia	59 (100.0)	4.7	3.8	3.5	0.8	20.3
	Subdoligranulum	58 (98.3)	4.3	2.8	3.7	0.0	13.1
	Prevotella	24 (40.7)	3.5	6.4	0.0	0.0	25.6
	Anaerostipes	58 (98.3)	3.3	2.4	2.9	0.0	9.0
	Coprococcus	54 (91.5)	3.1	2.6	2.2	0.0	13.5
	Agathobacter	55 (93.2)	3.0	3.2	2.3	0.0	14.7
	Alistipes	57 (96.6)	2.9	3.0	2.3	0.0	18.5
	Ruminococcus	55 (93.2)	2.6	2.0	2.3	0.0	7.3
	Clostridia_UCG-014	40 (67.8)	2.2	2.9	1.1	0.0	11.9
	Parabacteroides	55 (93.2)	2.0	2.2	1.0	0.0	11.1
	Eubacterium hallii group	59 (100.0)	1.6	1.4	1.2	0.1	6.8
	Lachnospiraceae_NK4A136 group	55 (93.2)	1.6	2.1	0.7	0.0	8.4
	Dorea	58 (98.3)	1.6	1.1	1.5	0.0	4.8

CU: catheter urine; vswab: vaginal swab; pswab: periurethral swab; SD: standard deviation

* Percentages refer to total number of analyzed samples per sample type
