# Supplementary material for: Longitudinal variability in the urinary microbiota of healthy premenopausal women and the relation to neighboring microbial communities: A pilot study
Source: PLoS One. 2022 Jan 14;17(1):e0262095. doi: 10.1371/journal.pone.0262095 (PMC8759677; doi:10.1371/journal.pone.0262095)
Supplement: S2 Table — (PDF) [file pone.0262095.s008.pdf]

S2 Table: Most frequently detected species in expanded urinary culture of CU samples

Genus	Species*	N= 67** (%)
Lactobacillus		**43 (64.2)**
	Lactobacillus crispatus	17 (25.4)
	Lactobacillus vaginalis	16 (23.9)
	Lactobacillus jensenii	12 (17.9)
	Lactobacillus iners	10 (14.9)
	Lactobacillus gasseri	7 (10.4)
	Lactobacillus species	4 (6.0)
	Lactobacillus acidophilus	1 (1.5)
	Lactobacillus rhamnosus	1 (1.5)
Gardnerella		**15 (22.4)**
	Gardnerella vaginalis	15 (22.4)
Propionibacterium		**9 (13.4)**
	Propionibacterium avidum	5 (7.5)
	Propionibacterium acnes	4 (6.0)
Streptococcus		**8 (11.9)**
	Streptococcus agalactiae	5 (7.5)
	Streptococcus anginosus	3 (4.5)
	Streptococcus mitis	1 (1.5)
Actinomyces		**5 (7.5)**
	Actinomyces neuii	4 (6.0)
	Actinomyces species	1 (1.5)
Bifidobacterium		**4 (6.0)**
	Bifidobacterium breve	2 (3.0)
	Bifidobacterium bifidum	1 (1.5)
	Bifidobacterium longum	1 (1.5)
Staphylococcus		**4 (6.0)**
	Staphylococcus epidermidis	4 (6.0)
	Staphylococcus lugdunensis	1 (1.5)
Finegoldia		**3 (4.5)**
	Finegoldia magna	3 (4.5)
**Other*****		11 (16.4)

CU: catheter urine;

* Some samples yielded multiple species of the same genus. Percentages refer to total number of 67 samples that were subjected to culture.

** No cultural growth in 8 samples (11.9%), 1 sample was not subjected to culture (F3U) due to low urine volume.

*** Other: anaerobic gram positive cocci without further species identification (2), *Enterococcus faecalis* (2), *Micrococcus luteus* (2), *Burkholderia species* (1), *Escherichia coli* (1), *Prevotella bivia* (1), *Aerococcus sanguicola* (1), *Peptoniphilus harei* (1), *Actinotignum schaalii* (1)
