# Supplementary material for: Longitudinal variability in the urinary microbiota of healthy premenopausal women and the relation to neighboring microbial communities: A pilot study
Source: PLoS One. 2022 Jan 14;17(1):e0262095. doi: 10.1371/journal.pone.0262095 (PMC8759677; doi:10.1371/journal.pone.0262095)
Supplement: S3 Table — (PDF) [file pone.0262095.s009.pdf]

S3 Table: Comparison of urinary microbiota results of 16S rRNA gene sequencing and expanded culture of CU

**Result**	**All samples N=63 (%)**
All cultured taxa* present in sequencing results	44 (69.8)
- and all cultured taxa with a relative abundance of >10%	21 (33.3)
- but at least one with a relative abundance >10%	9 (14.3)
- but none with a relative abundance >10%	14 (22.2)
Not all cultured taxa present in sequencing results**	10 (15.9)
None of the cultured taxa present in sequencing results	1 (1.6)
No bacterial growth in expanded culture	8 (12.7)

CU: catheter urine

* Cultured genera were compared with either the taxa found on genus level or in cases without detection on genus level with taxa found on family level

** Cultured bacteria not confirmed by sequencing: 2x *Propionibacterium acnes* (after enrichment; e); 3x *Actinomyces neuii* ($1x10^3$ CFU/ml; 2xe); 2x *Micrococcus luteus* (10^3 CFU/ml; e); *Lactobacillus rhamnosus* (e); *Gardnerella vaginalis* (e); *Bifidobacterium bifidum*; *Staphylococcus epidermidis* (e); *Streptococcus anginosus* (e); *Peptoniphilus harei* (e); *Actinomyces species* (e); *Propionibacterium avidum* (e)

**** *Bifidobacterium longum* (after enrichment; e), *Lactobacillus species* (e); *Gardnerella vaginalis* (e); *Actinomyces species* (e); *Propionibacterium avidum* (e)
