# Supplementary material for: Longitudinal variability in the urinary microbiota of healthy premenopausal women and the relation to neighboring microbial communities: A pilot study
Source: PLoS One. 2022 Jan 14;17(1):e0262095. doi: 10.1371/journal.pone.0262095 (PMC8759677; doi:10.1371/journal.pone.0262095)
Supplement: S4 Table — (PDF) [file pone.0262095.s010.pdf]

S4 Table: Comparison of cultural results from CU and corresponding MU samples

Sample visit ID	Cultured species CU sample (CFU/ml)	Cultured species MU sample (CFU/ml)	Concordance of detected species
A5	*Lactobacillus crispatus* (10^{3})	*Lactobacillus crispatus* (10^{4})	CU species in MU sample, but additional species in MU
		Streptococcus mutans (10^{3})	
		Bifidobacterium breve (10^{3})	
		Staphylococcus lugdunensis (e)	
		Staphylococcus epidermidis (e)	
B5	*Streptococcus agalactiae* (e)	*Streptococcus agalactiae* (e)	CU species in MU sample, but additional species in MU
		Lactobacillus crispatus (10^{3})	
		Enterococcus faecalis (e)	
		Staphylococcus epidermidis (e)	
C5	*Gardnerella vaginalis* (10^{3})	*Gardnerella vaginalis* (10^{6})	CU species in MU sample, but additional species in MU
	Lactobacillus rhamnosus (e)	*Lactobacillus rhamnosus* (10^{3})	
		Lactobacillus fermentum (10^{3})	
		Streptococcus anginosus (10^{3})	
		Prevotella bivia (10^{3})	
D5	*Streptococcus anginosus* (e)	*Streptococcus anginosus* (10^{3})	Not all CU species in MU samples and additional species in MU
	Streptococcus agalactiae (e)	*Lactobacillus crispatus* (10^{4})	
		Propionibacterium avidum (10^{3})	
		Lactobacillus jensenii (10^{3})	
		Prevotella disiens (10^{3})	
H5	*Lactobacillus crispatus* (10^{4})	*Lactobacillus crispatus* (10^{4})	Not all CU species in MU samples and additional species in MU
	Streptococcus anginosus (e)	*Streptococcus anginosus* (10^{3})	
	Peptoniphilus harei (e)	*Staphylococcus epidermidis* (10^{3})	
I5	*No bacterial growth*	*Lactobacillus crispatus* (10^{4})	No concordance
		Streptococcus anginosus (10^{3})	
J5	*Lactobacillus crispatus* (e)	*Lactobacillus crispatus* (10^{4})	Same species in CU and MU
L5	*Streptococcus agalactiae* (e)	*Streptococcus agalactiae* (10^{3})	CU species in MU sample, but additional species in MU
		Lactobacillus crispatus (10^{5})	
M5	*Lactobacillus gasseri* (e)	*Lactobacillus gasseri* (10^{4})	Not all CU species in MU samples and additional species in MU
	Lactobacillus crispatus (e)	*Lactobacillus jensenii* (10^{3})	
	Propionibacterium avidum (e)	*Staphylococcus epidermidis* (10^{3})	
		Bifidobacterium bifidum (e)	
		Actinomyces neuii (e)	
		Campylobacter ureolyticus (e)	
N5	*Gardnerella vaginalis* (10^{3})	*Gardnerella vaginalis* (10^{3})	CU species in MU sample, but additional species in MU
		Propionibacterium acnes (10^{3})	
		Lactobacillus jensenii (10^{3})	
		Staphylococcus condimenti (e)	
		Prevotella bivia (e)	

CU: catheter urine; (e): detection only after enrichment, no quantification performed
