# Supplementary material for: Longitudinal variability in the urinary microbiota of healthy premenopausal women and the relation to neighboring microbial communities: A pilot study
Source: PLoS One. 2022 Jan 14;17(1):e0262095. doi: 10.1371/journal.pone.0262095 (PMC8759677; doi:10.1371/journal.pone.0262095)
Supplement: S1 File — (PDF) [file pone.0262095.s011.pdf]

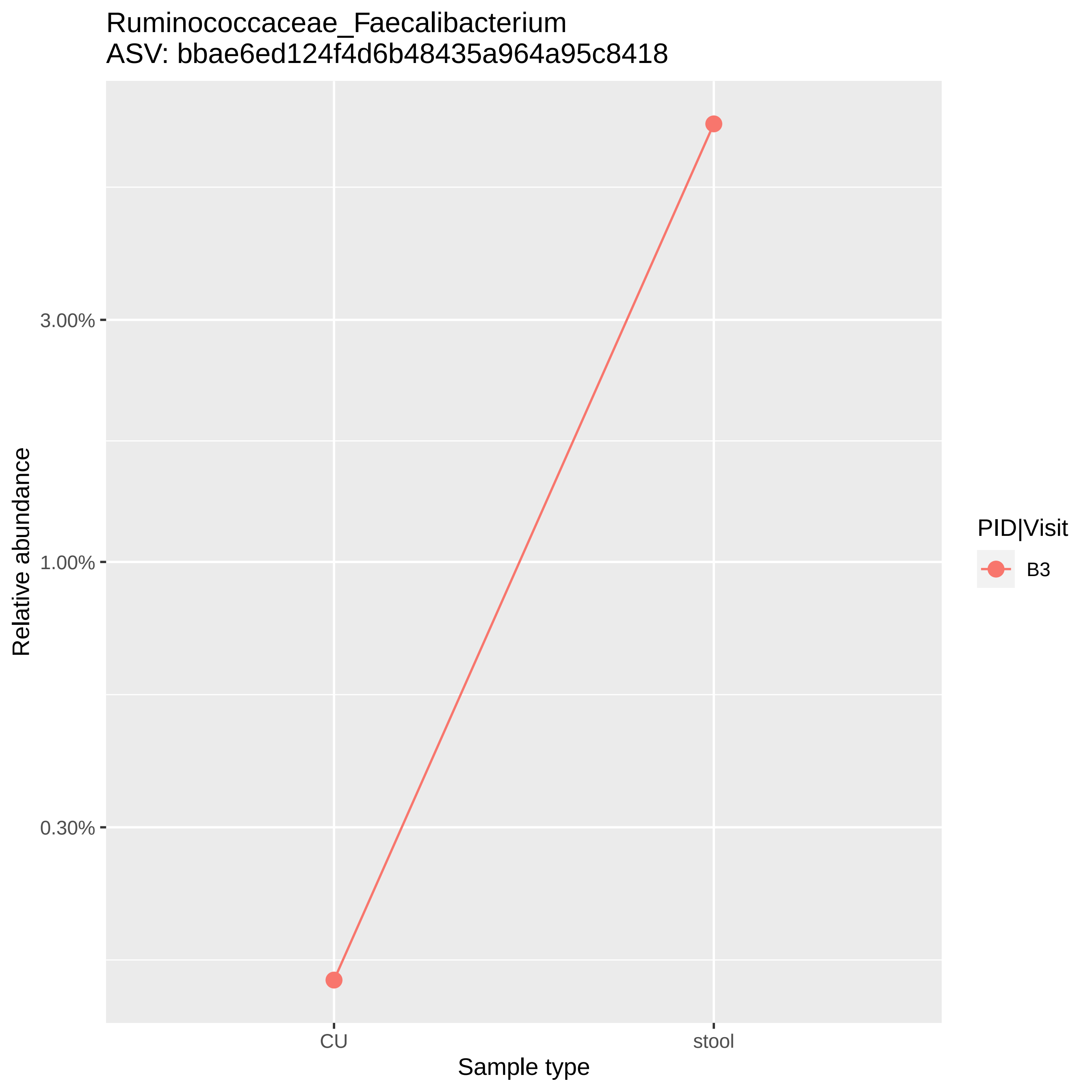
Ruminococcaceae_Faecalibacterium
ASV: bbae6ed124f4d6b48435a964a95c8418
Relative abundance
3.00%
1.00%
0.30%
CU
stool
Sample type
PID|Visit
B3

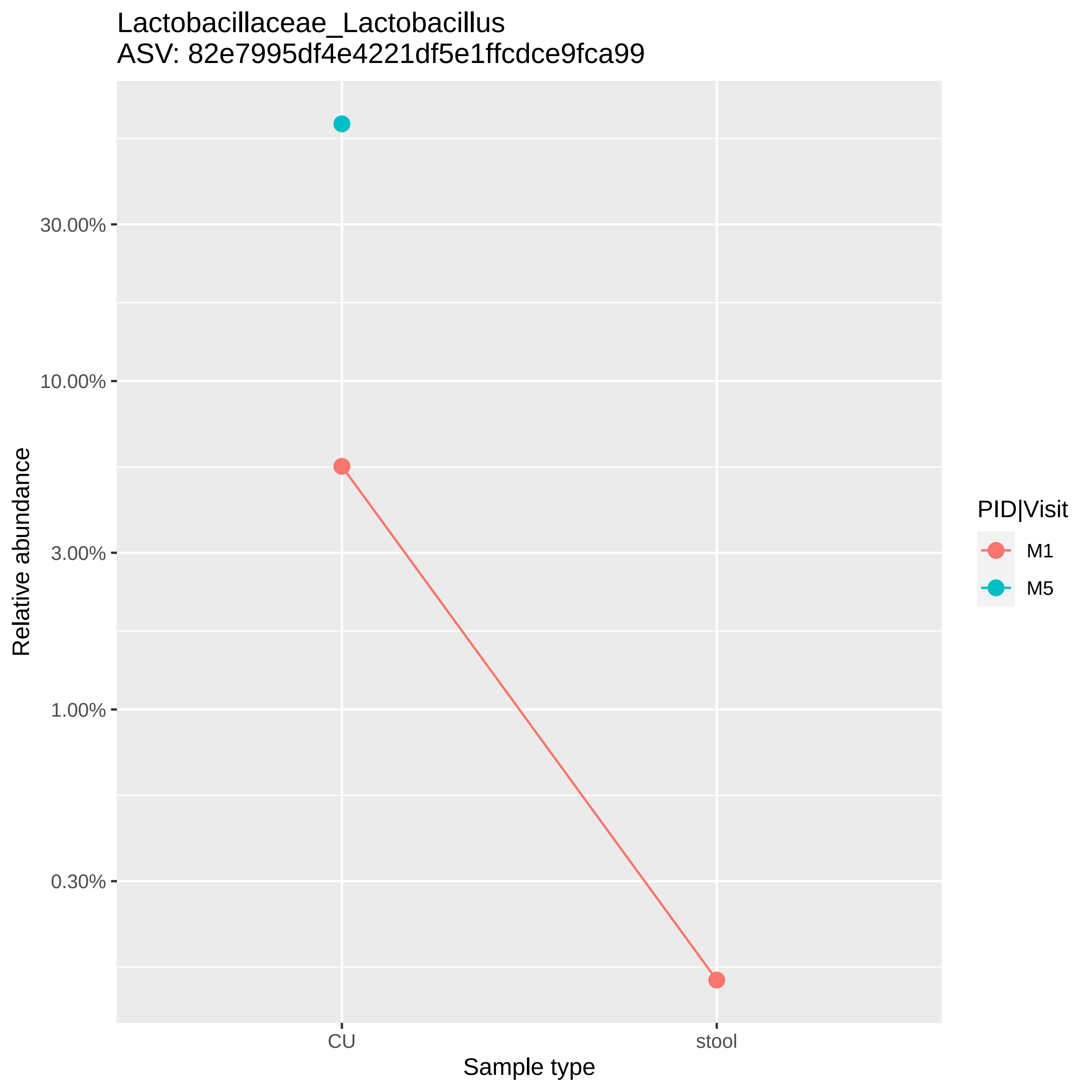

Lactobacillaceae_Lactobacillus
ASV: 82e7995df4e4221df5e1ffcdce9fca99
Relative abundance
30.00%
10.00%
3.00%
1.00%
0.30%
CU
stool
Sample type
PID|Visit
M1
M5

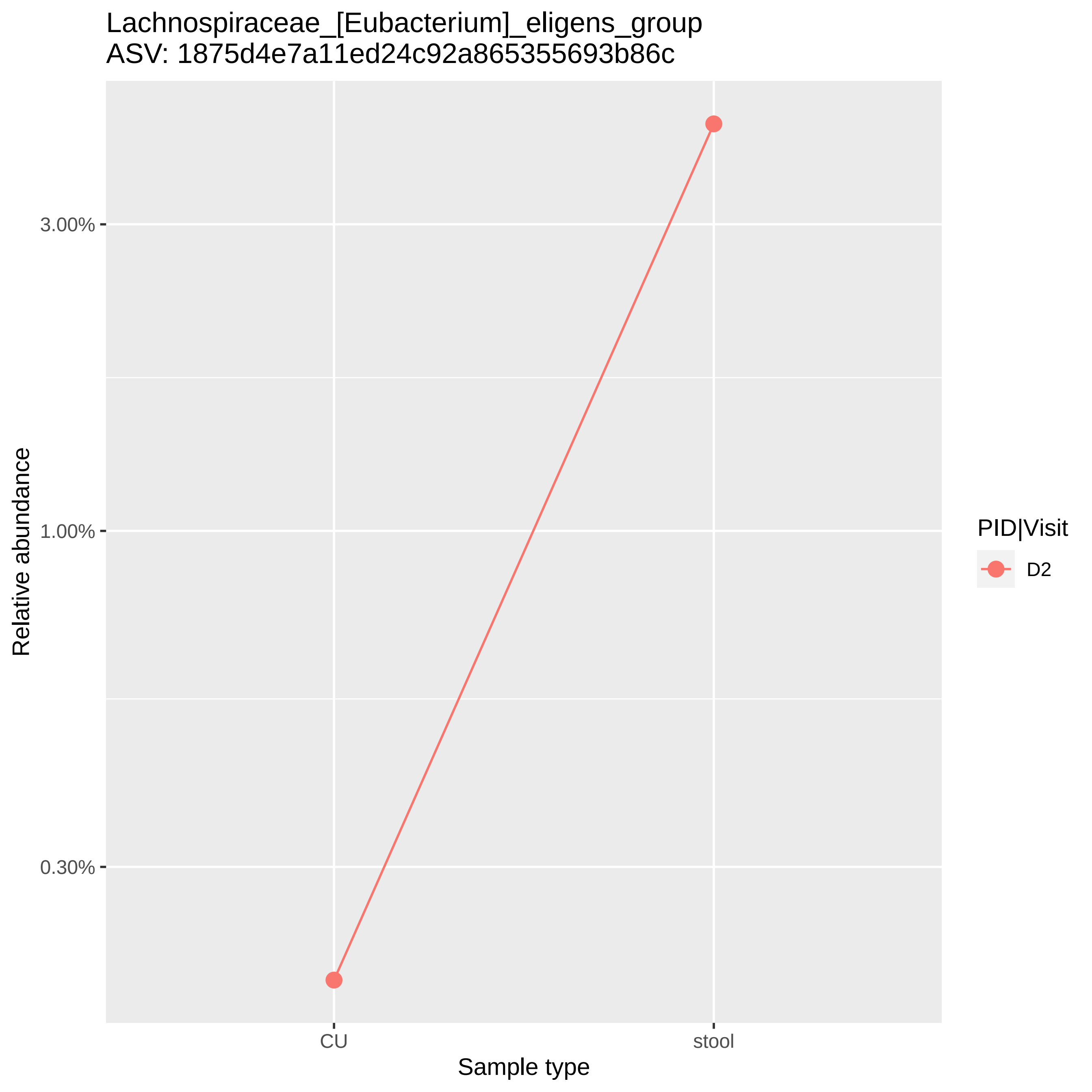
Lachnospiraceae_[Eubacterium]_eligens_group
ASV: 1875d4e7a11ed24c92a865355693b86c
Relative abundance
3.00%
1.00%
0.30%
CU
stool
Sample type
PID|Visit
D2

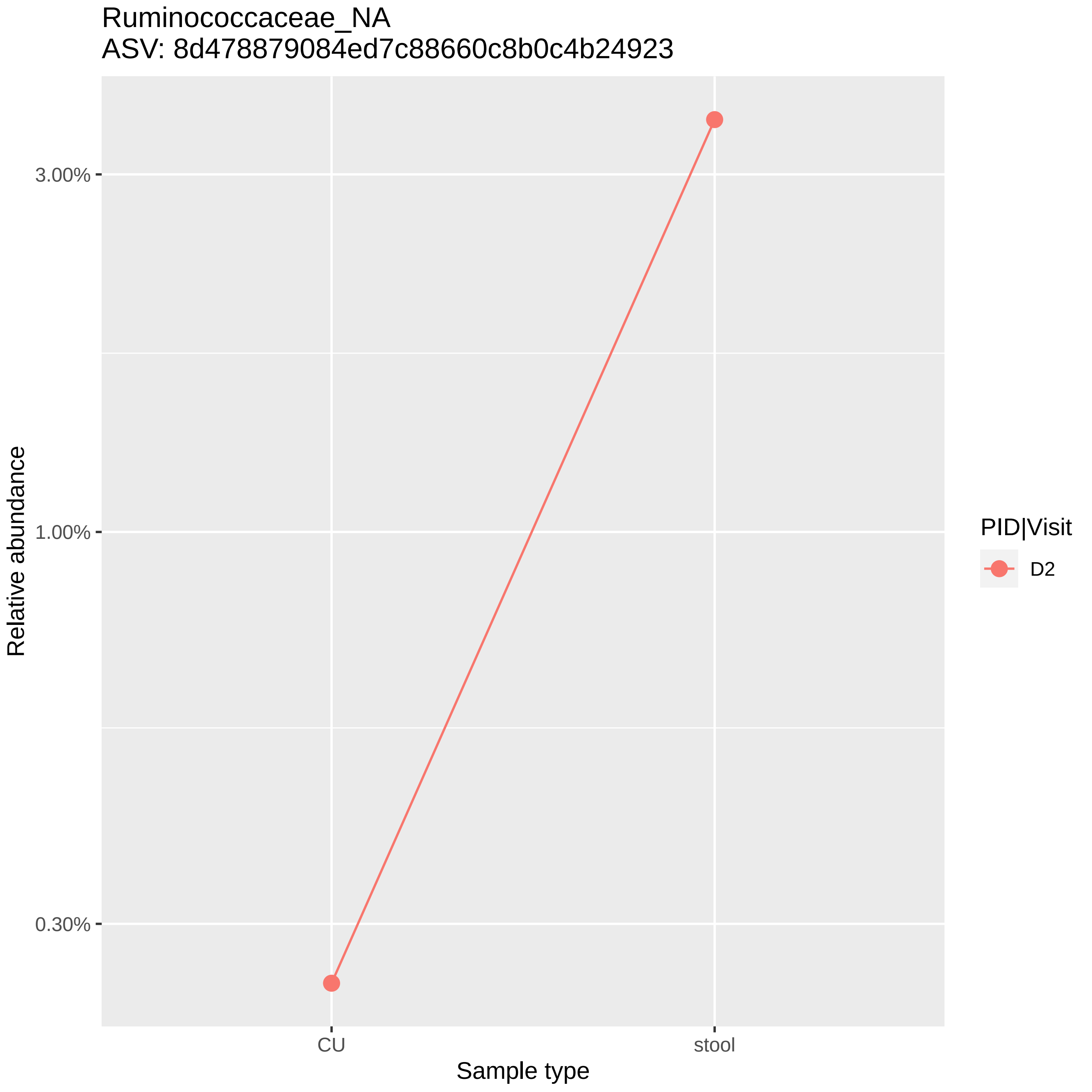

Ruminococcaceae_NA
ASV: 8d478879084ed7c88660c8b0c4b24923
3.00%
1.00%
0.30%
Relative abundance
CU
stool
Sample type
PID|Visit
D2

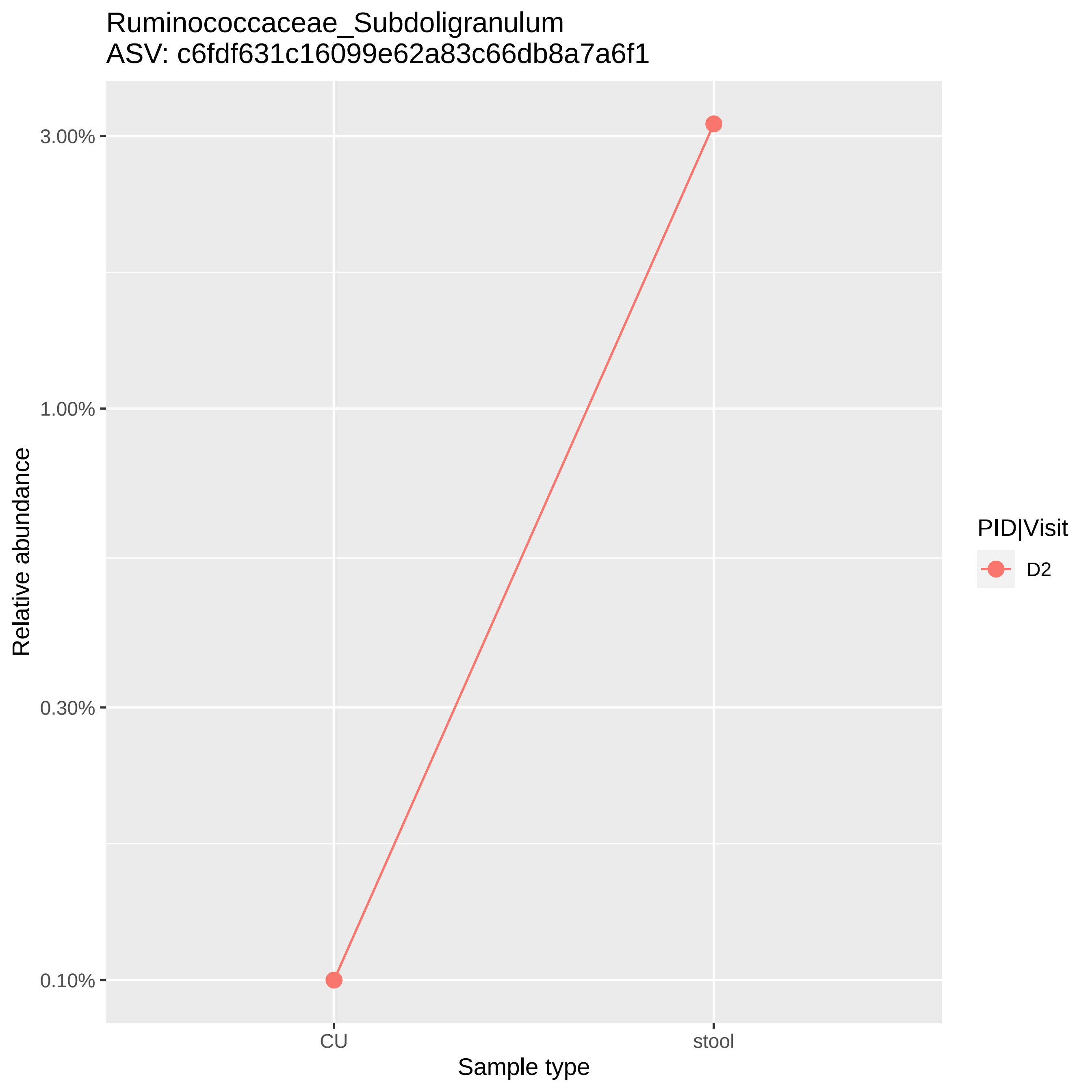
Ruminococcaceae_Subdoligranulum
ASV: c6fdf631c16099e62a83c66db8a7a6f1
3.00%
1.00%
0.30%
0.10%
Relative abundance
CU
stool
Sample type
PID|Visit
D2

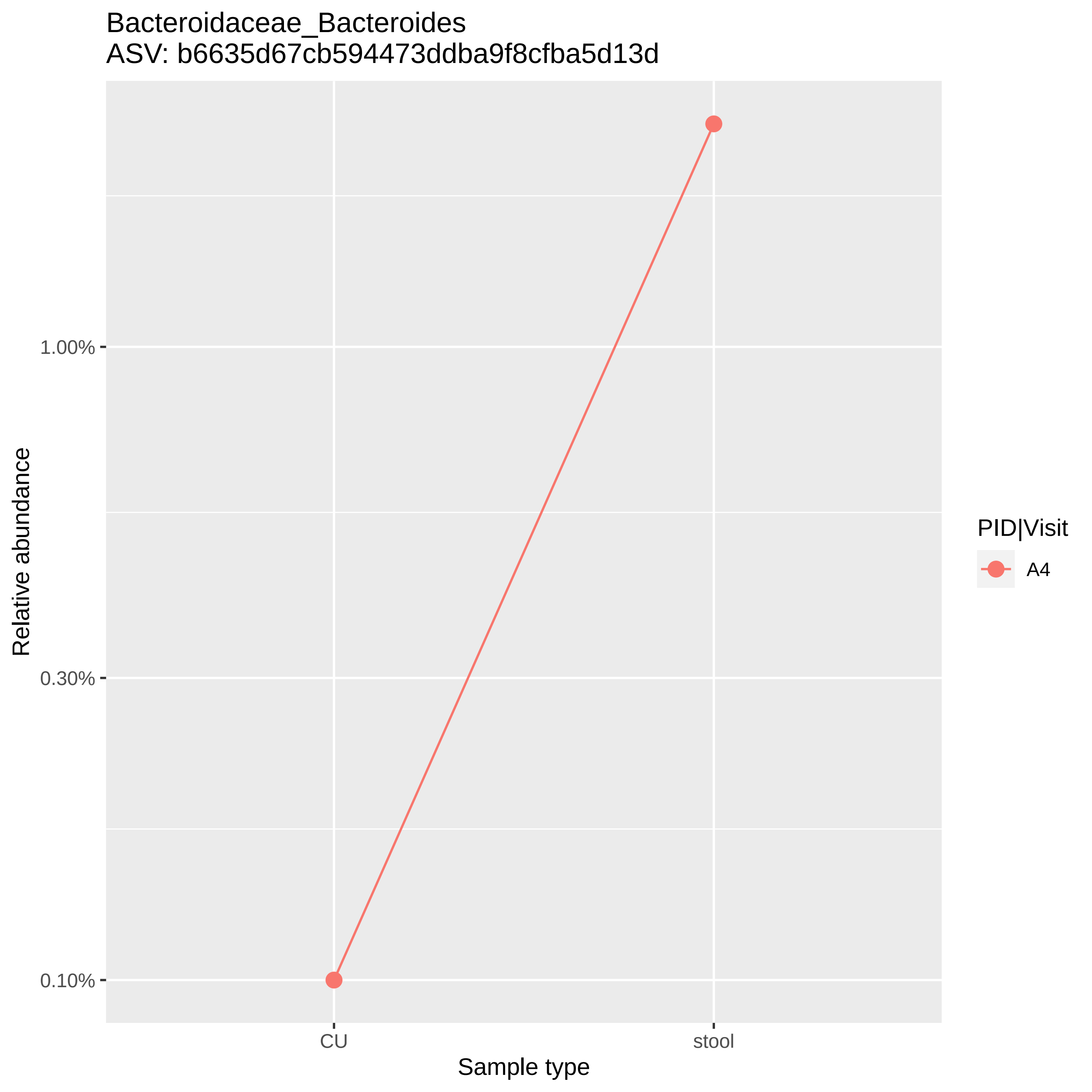
Bacteroidaceae_Bacteroides
ASV: b6635d67cb594473ddba9f8cfba5d13d
Relative abundance
1.00%
0.30%
0.10%
CU
stool
Sample type
PID|Visit
A4

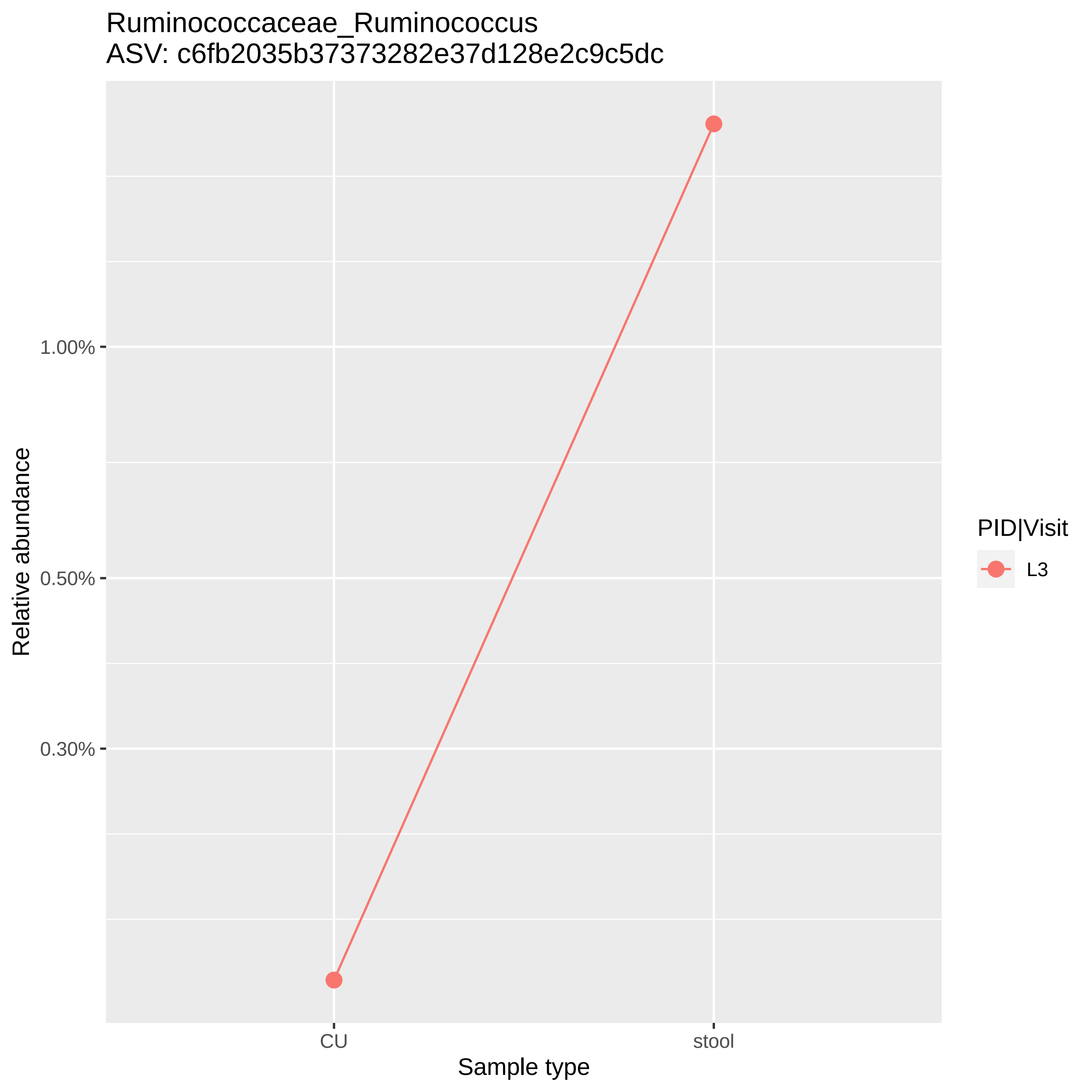
Ruminococcaceae_Ruminococcus
ASV: c6fb2035b37373282e37d128e2c9c5dc
Relative abundance
1.00%
0.50%
0.30%
CU
stool
Sample type
PID|Visit
L3

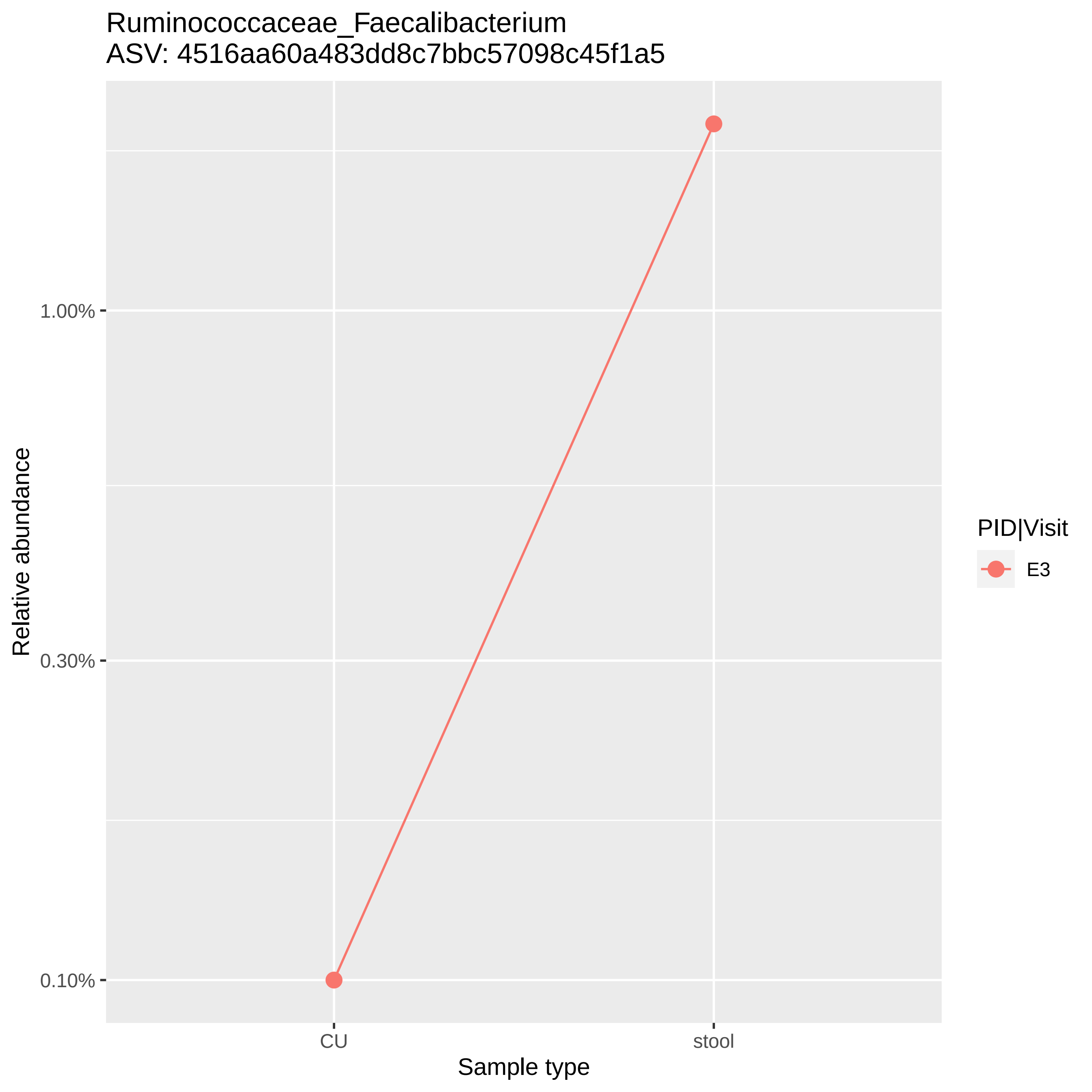
Ruminococcaceae_Faecalibacterium
ASV: 4516aa60a483dd8c7bbc57098c45f1a5
Relative abundance
1.00%
0.30%
0.10%
CU
stool
Sample type
PID|Visit
E3

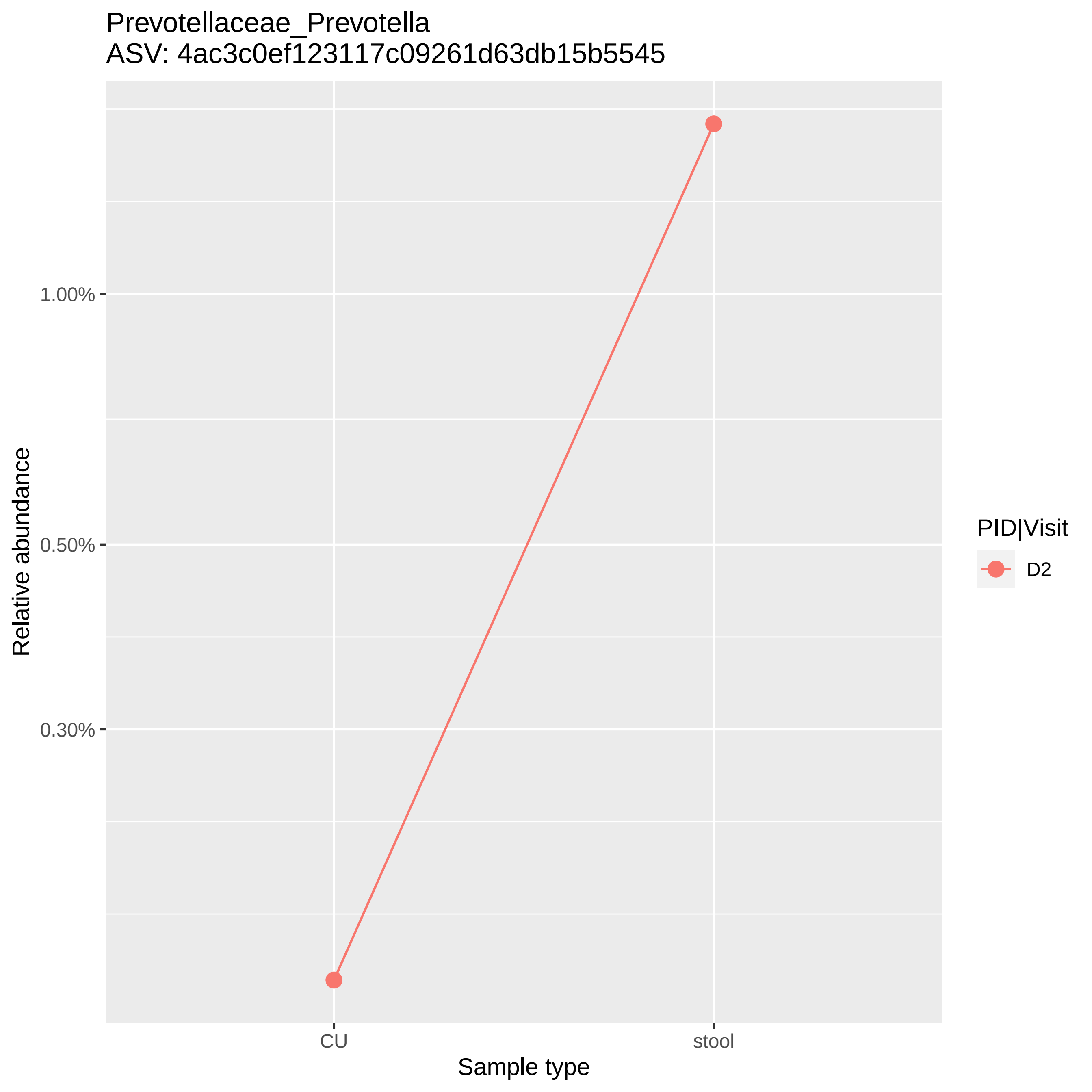

Prevotellaceae_Prevotella
ASV: 4ac3c0ef123117c09261d63db15b5545
Relative abundance
1.00%
0.50%
0.30%
CU
stool
Sample type
PID|Visit
D2

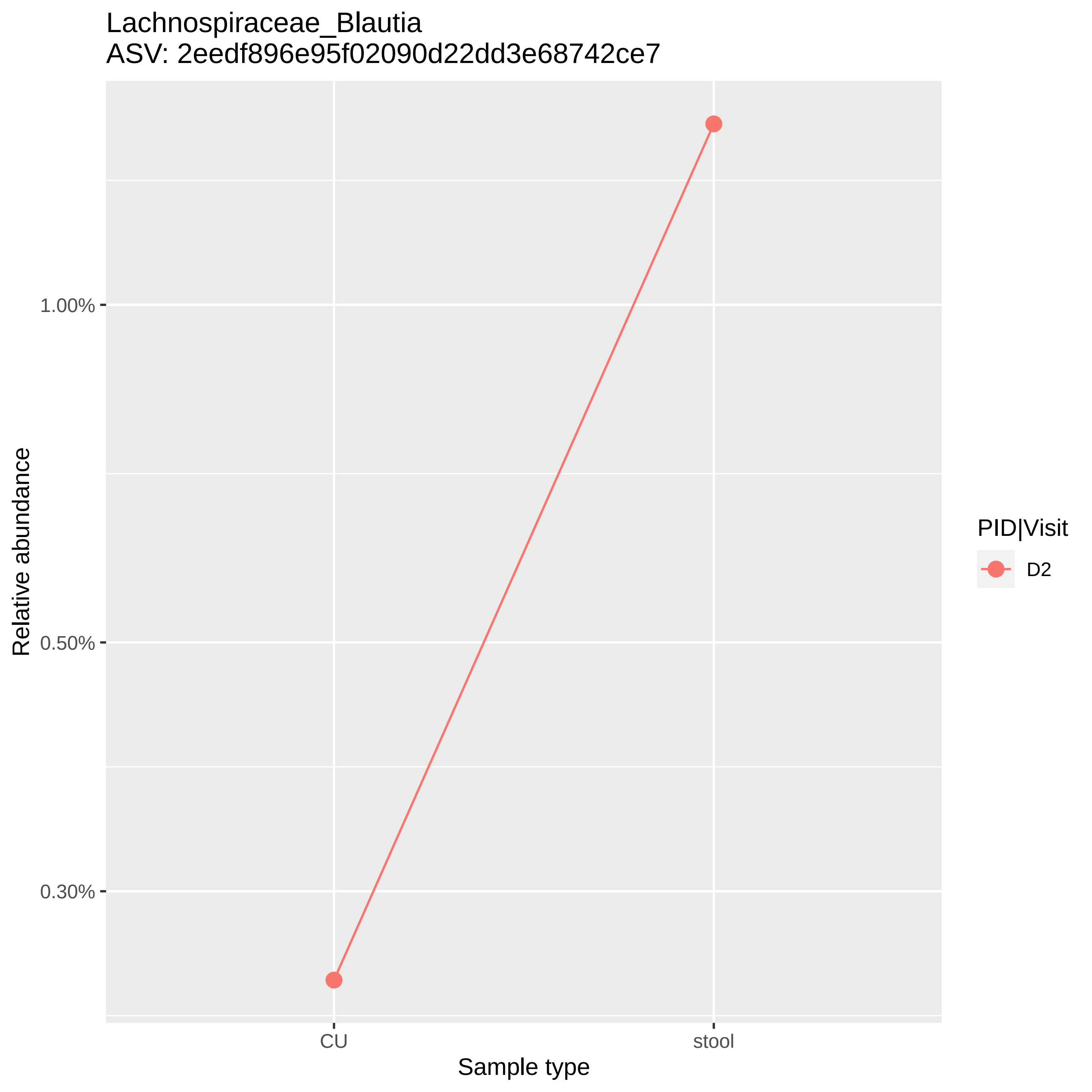
Lachnospiraceae_Blautia
ASV: 2eedf896e95f02090d22dd3e68742ce7
Relative abundance
1.00%
0.50%
0.30%
CU
stool
Sample type
PID|Visit
D2

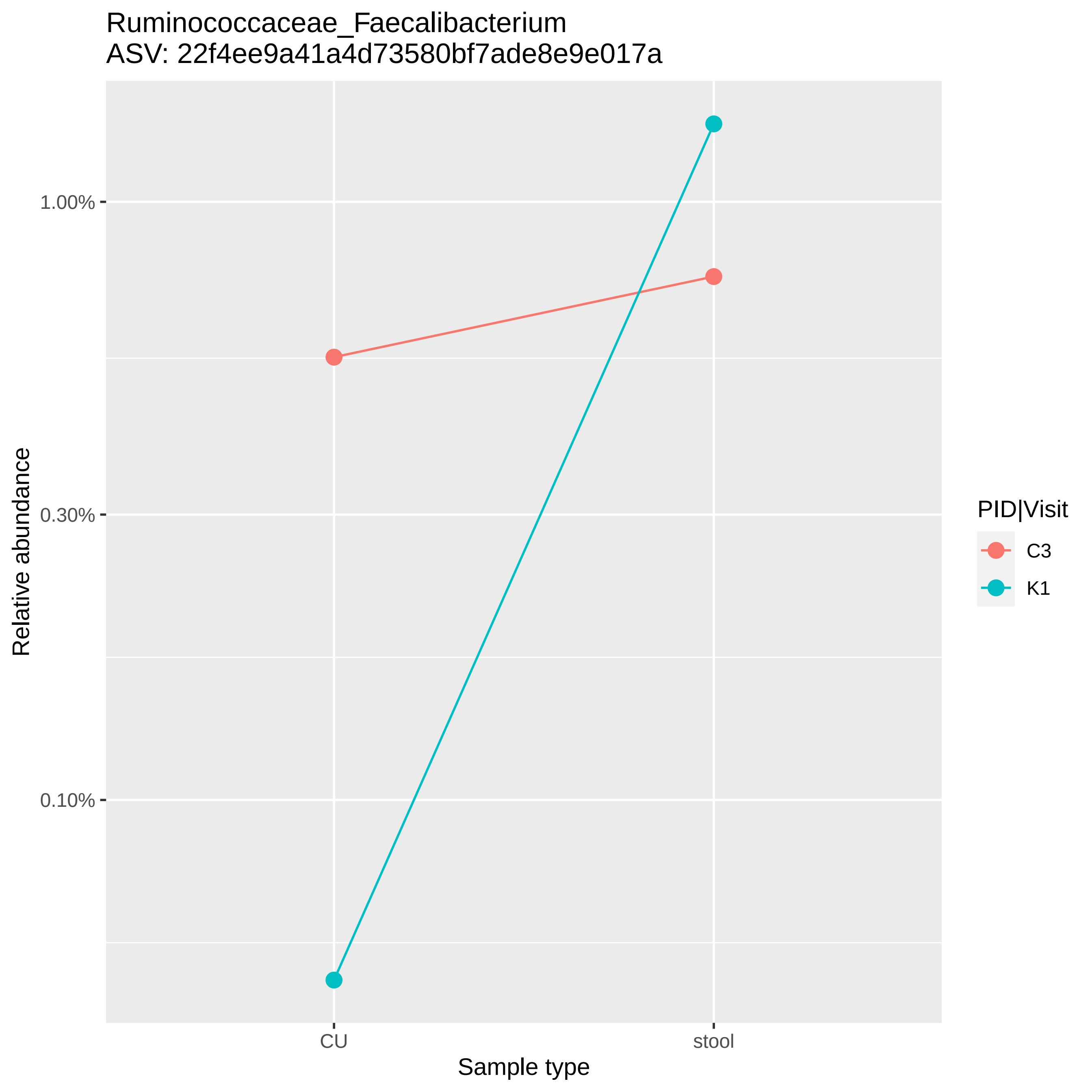
Ruminococcaceae_Faecalibacterium
ASV: 22f4ee9a41a4d73580bf7ade8e9e017a
1.00%
0.30%
0.10%
Relative abundance
CU
stool
Sample type
PID|Visit
C3
K1

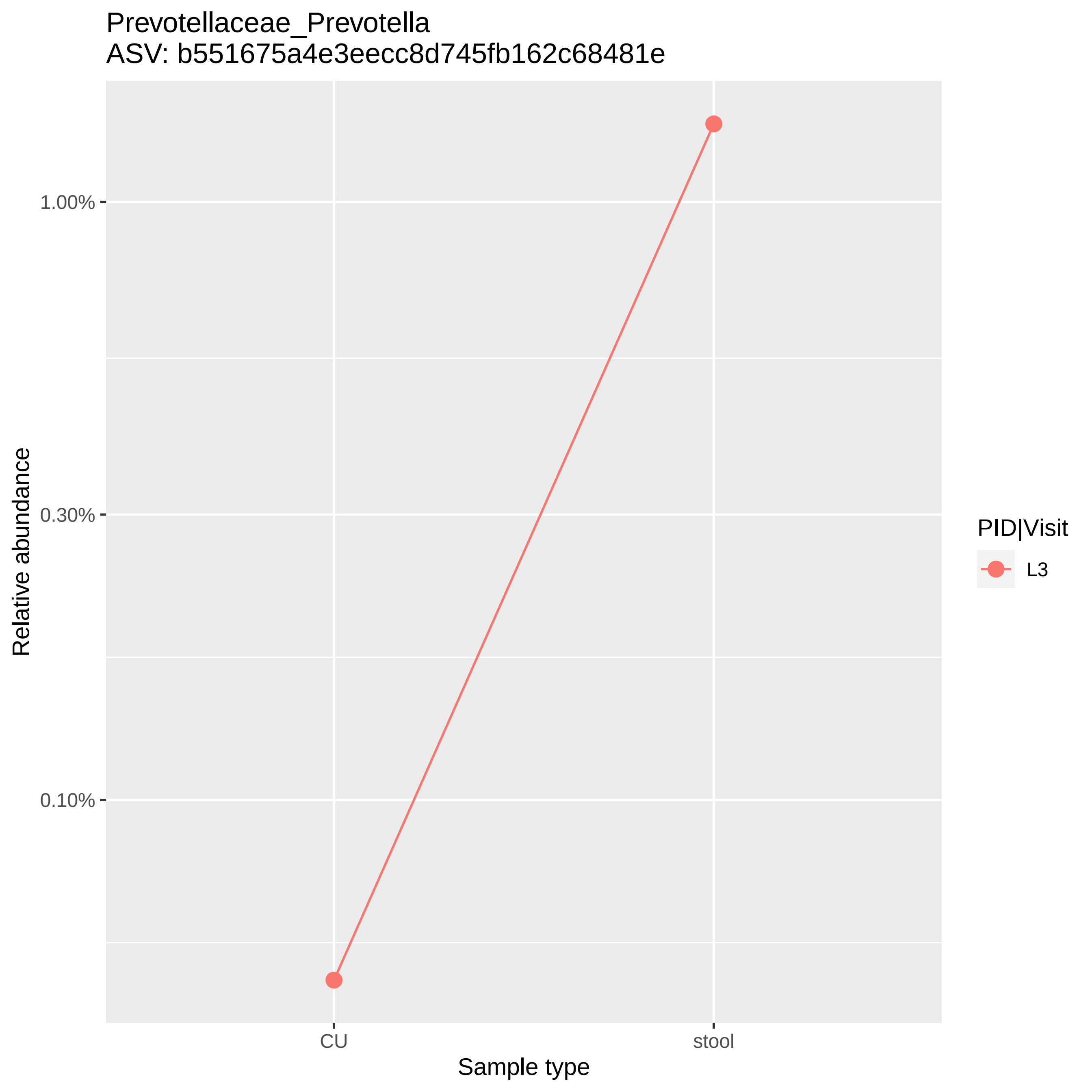
Prevotellaceae_Prevotella
ASV: b551675a4e3eecc8d745fb162c68481e
1.00%
0.30%
0.10%
Relative abundance
CU
stool
Sample type
PID|Visit
L3

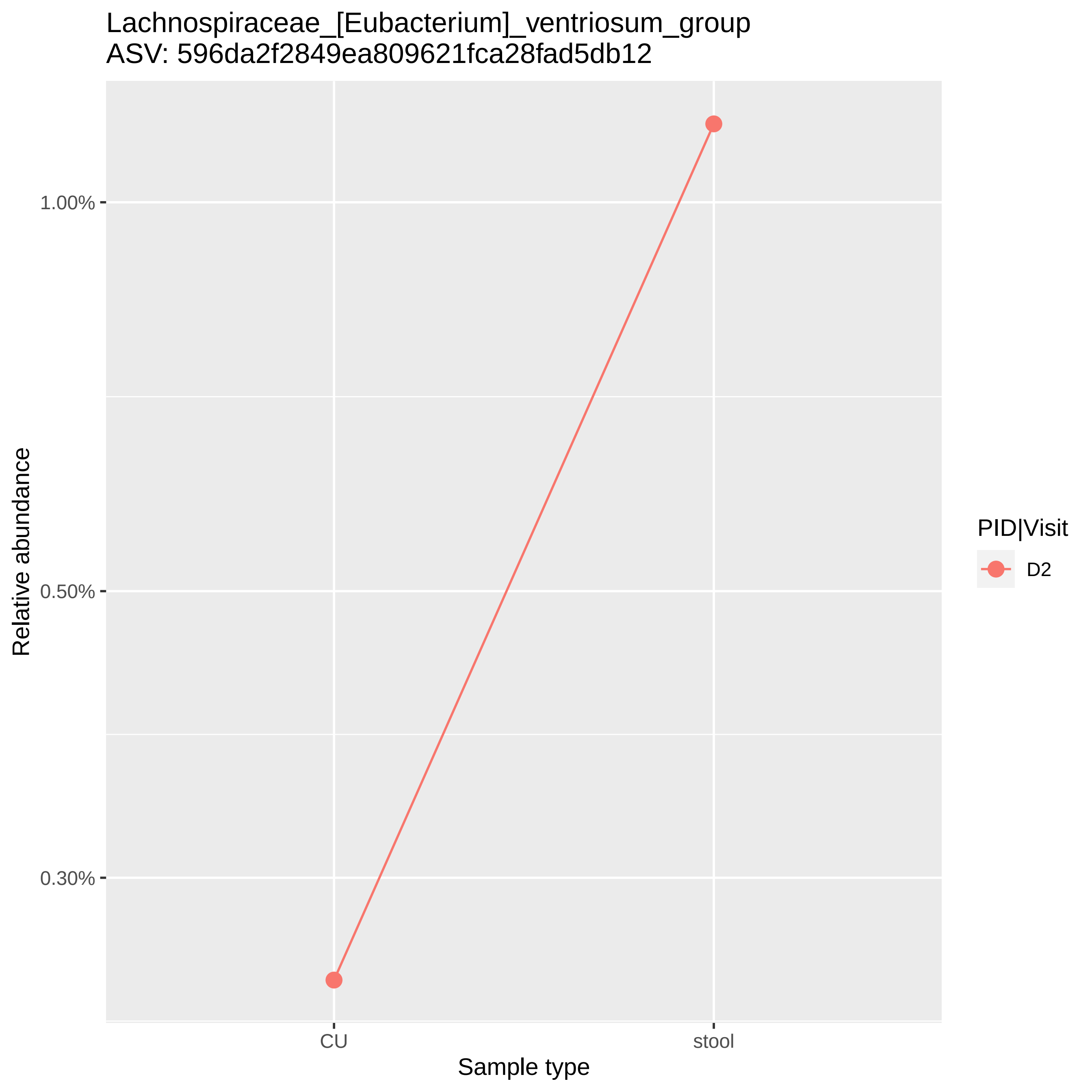
Lachnospiraceae_[Eubacterium]_ventriosum_group
ASV: 596da2f2849ea809621fca28fad5db12
1.00%
0.50%
0.30%
Relative abundance
CU
stool
Sample type
PID|Visit
D2

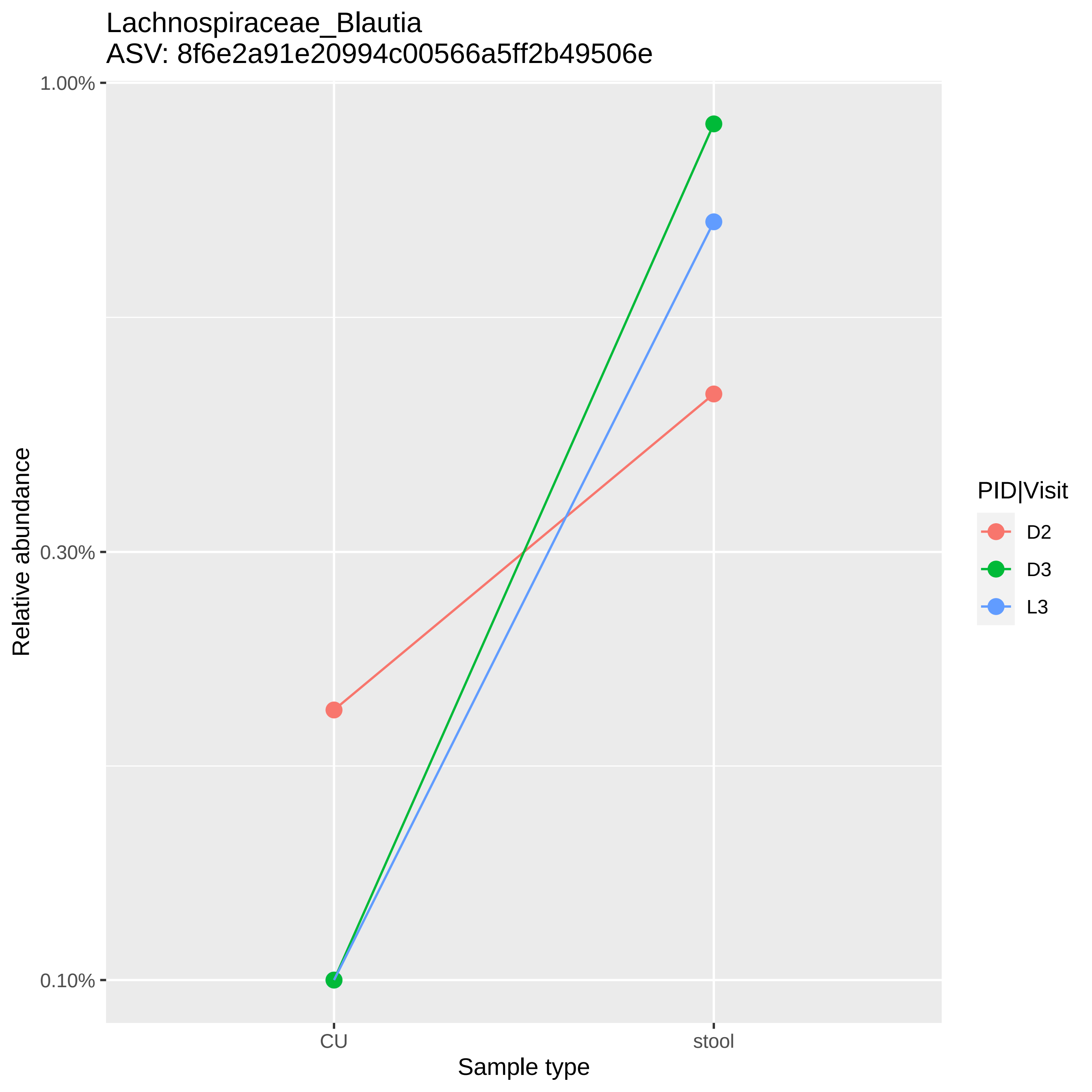

Lachnospiraceae_Blautia
ASV: 8f6e2a91e20994c00566a5ff2b49506e
1.00%
0.30%
0.10%
Relative abundance
CU
stool
Sample type
PID|Visit
D2
D3
L3

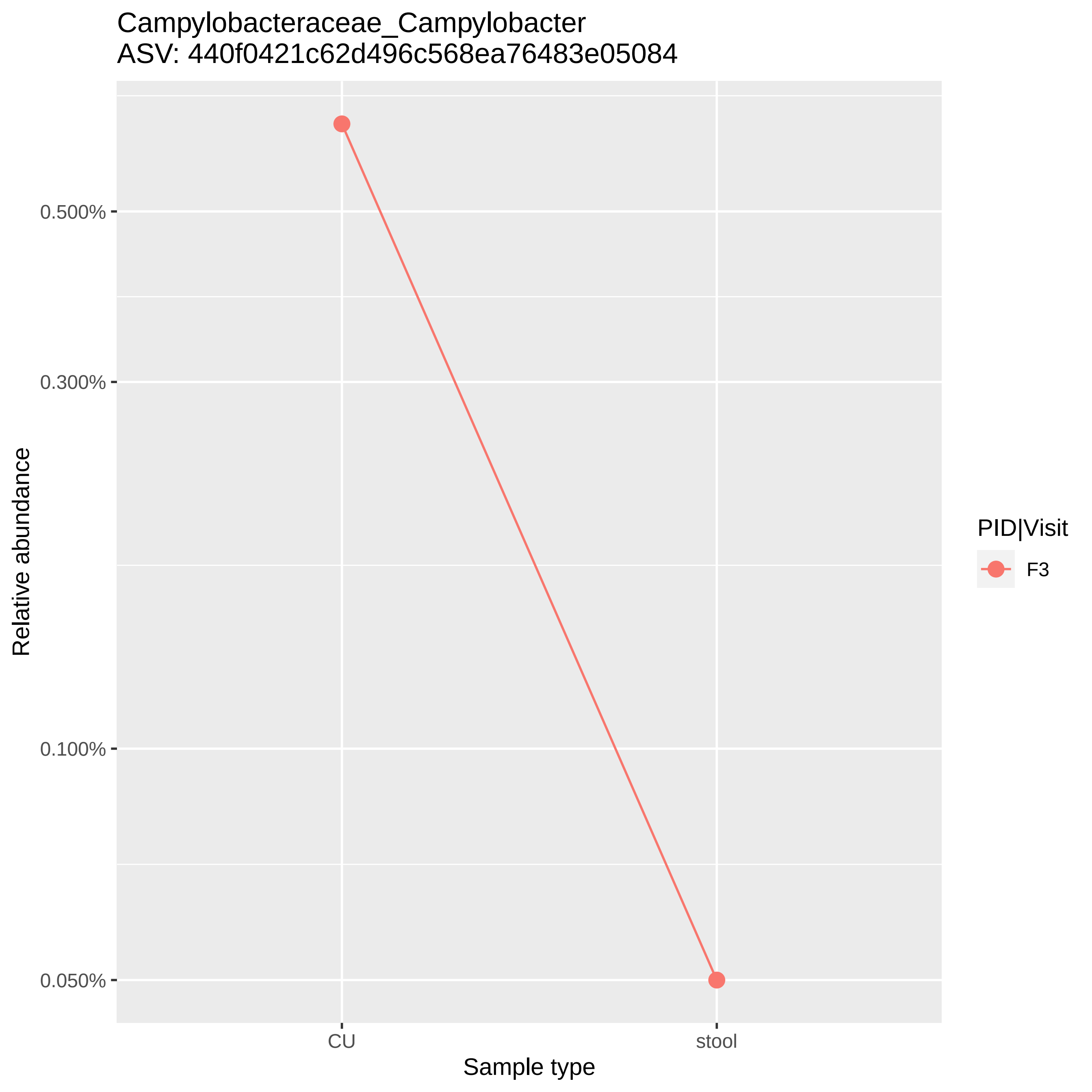

Campylobacteraceae_Campylobacter
ASV: 440f0421c62d496c568ea76483e05084
0.500%
0.300%
0.100%
0.050%
Relative abundance
CU
stool
Sample type
PID|Visit
F3

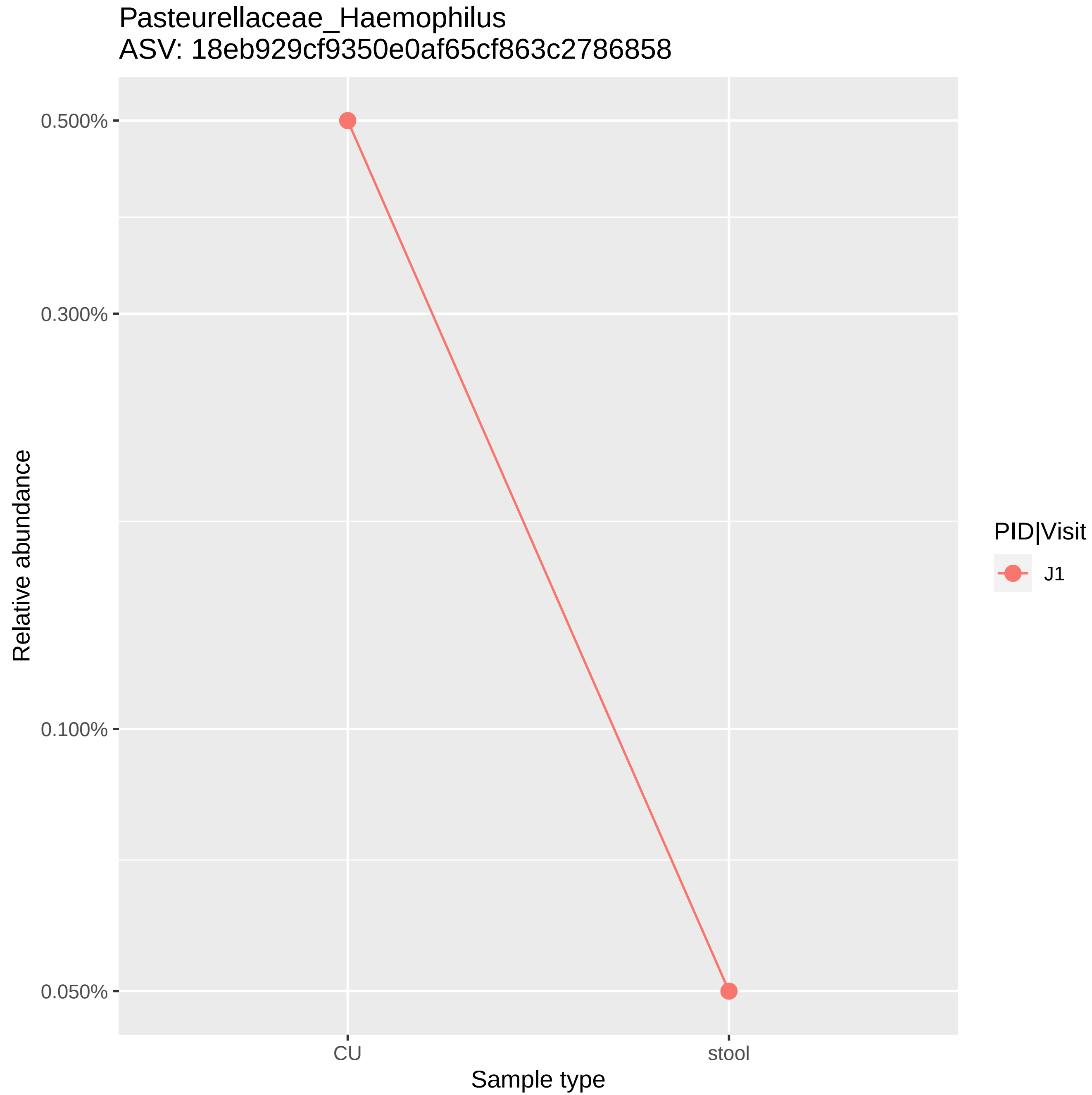

Pasteurellaceae_Haemophilus
ASV: 18eb929cf9350e0af65cf863c2786858
0.500%
0.300%
0.100%
0.050%
Relative abundance
CU
stool
Sample type
PID|Visit
J1

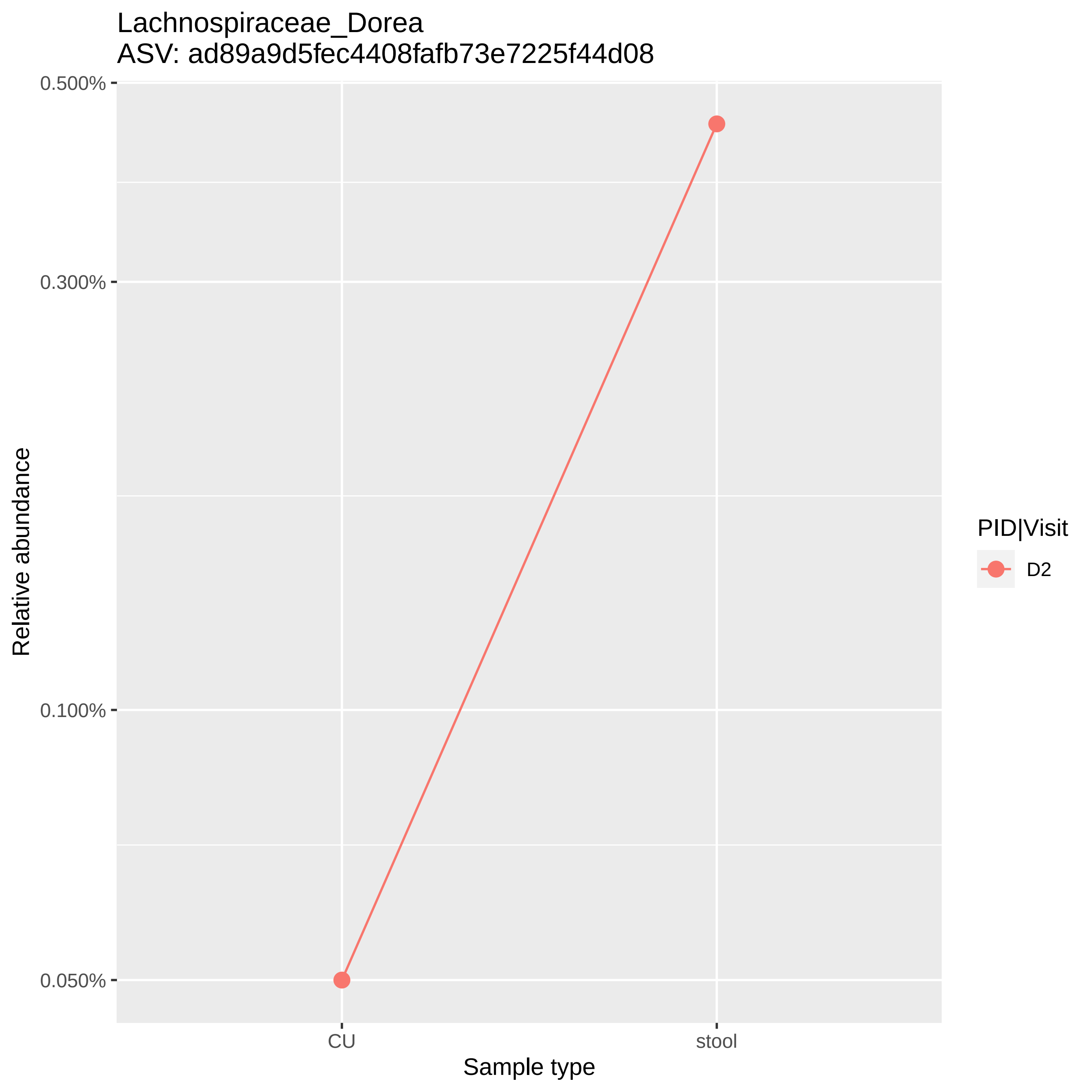

Lachnospiraceae_Dorea
ASV: ad89a9d5fec4408fafb73e7225f44d08
0.500%
0.300%
0.100%
0.050%
Relative abundance
CU
stool
Sample type
PID|Visit
D2

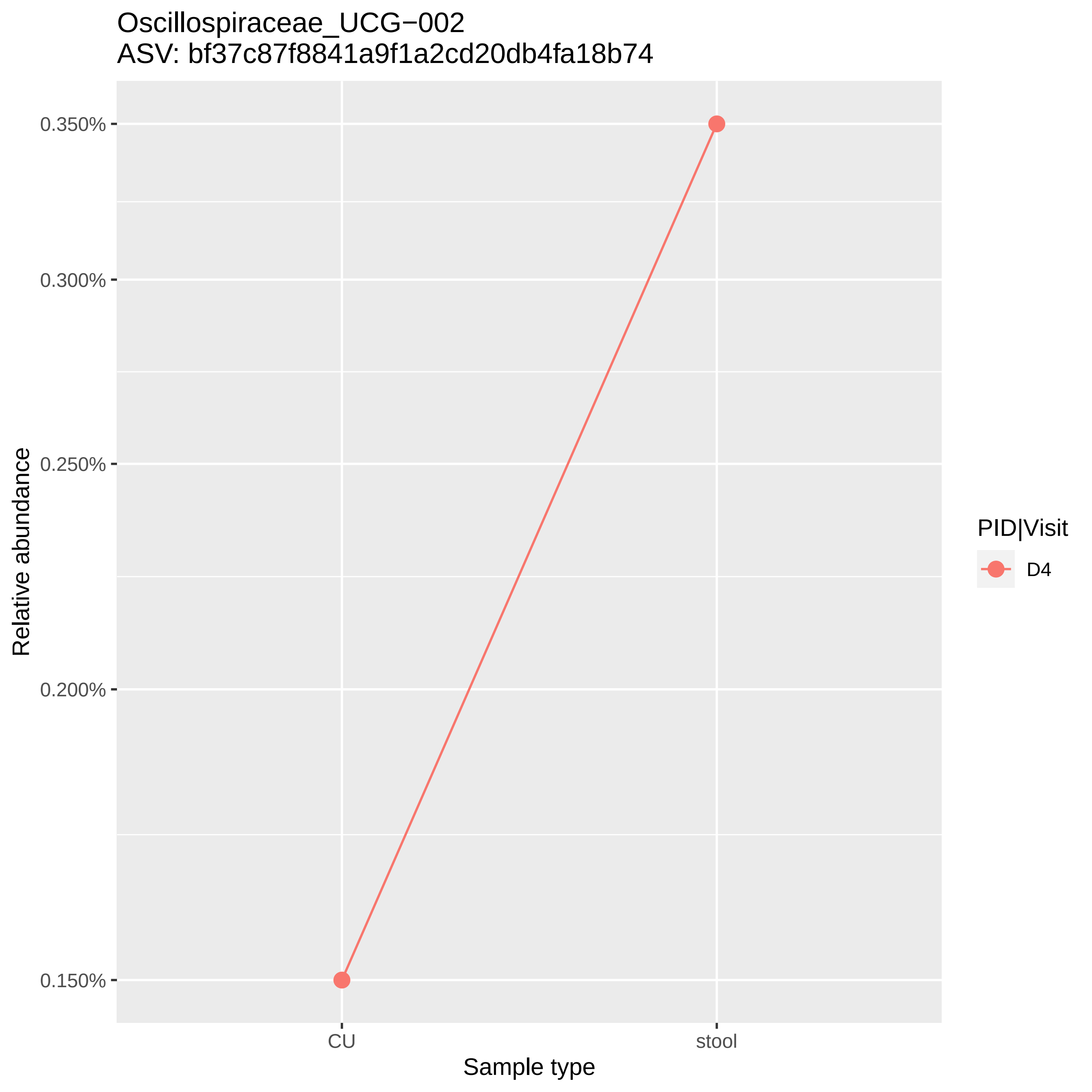
Oscillospiraceae_UCG−002
ASV: bf37c87f8841a9f1a2cd20db4fa18b74
0.350%
0.300%
0.250%
0.200%
0.150%
Relative abundance
CU
stool
Sample type
PID|Visit
D4

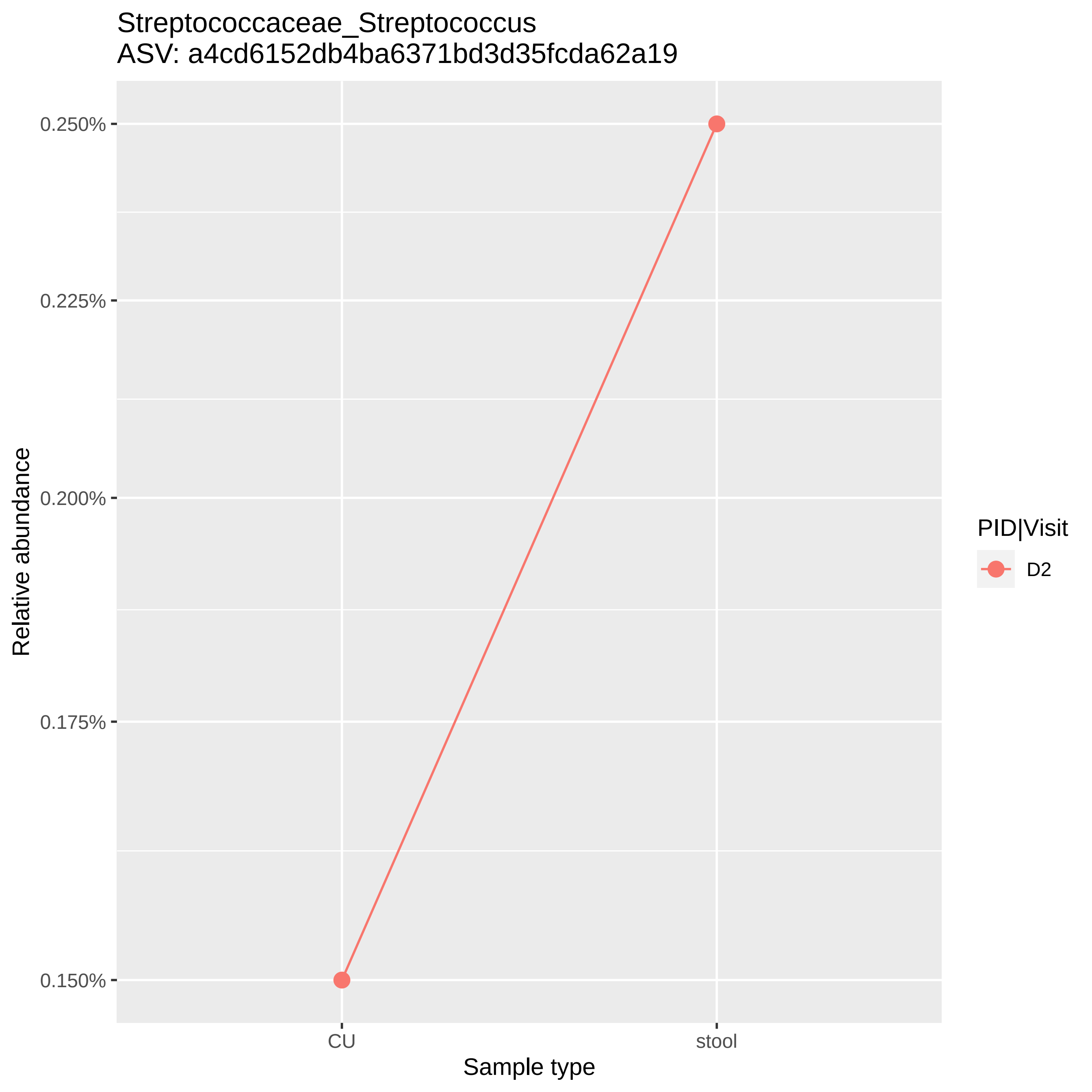
Streptococcaceae_Streptococcus
ASV: a4cd6152db4ba6371bd3d35fcda62a19
0.250%
0.225%
0.200%
0.175%
0.150%
Relative abundance
CU
stool
Sample type
PID|Visit
D2

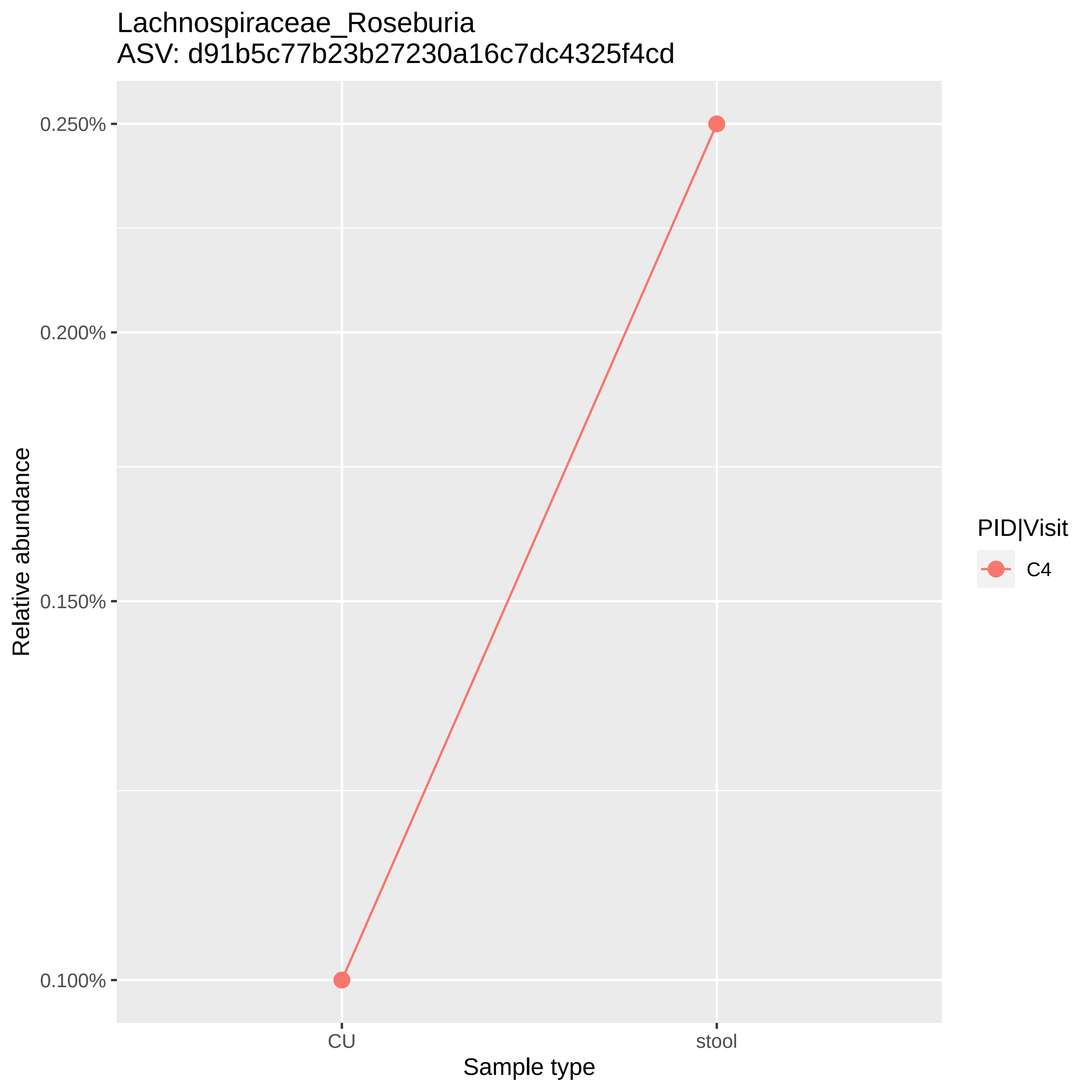
Lachnospiraceae_Roseburia
ASV: d91b5c77b23b27230a16c7dc4325f4cd
0.250%
0.200%
0.150%
0.100%
Relative abundance
CU
stool
Sample type
PID|Visit
C4
