# Supplementary material for: Longitudinal variability in the urinary microbiota of healthy premenopausal women and the relation to neighboring microbial communities: A pilot study
Source: PLoS One. 2022 Jan 14;17(1):e0262095. doi: 10.1371/journal.pone.0262095 (PMC8759677; doi:10.1371/journal.pone.0262095)
Supplement: S2 File — (PDF) [file pone.0262095.s012.pdf]

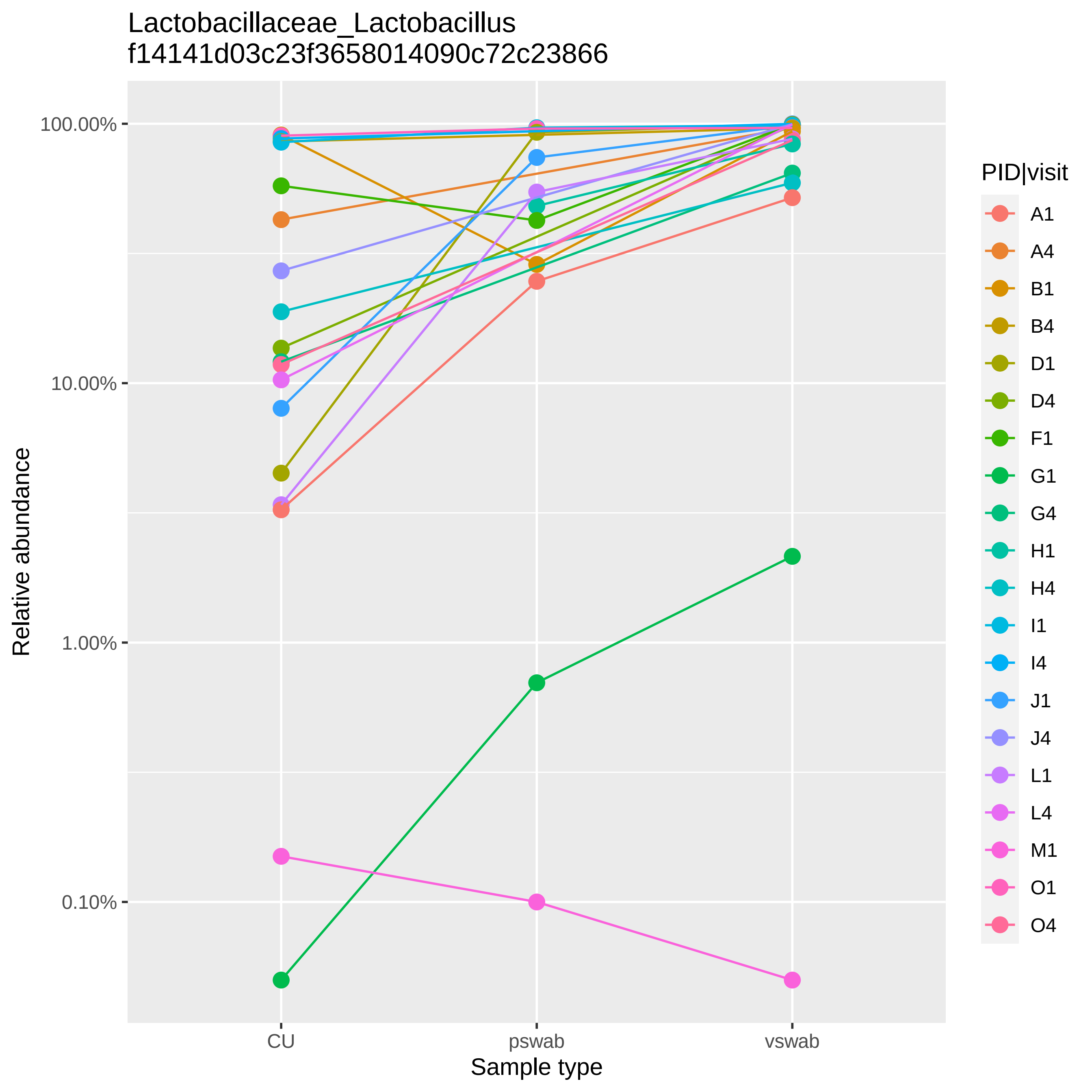

Lactobacillaceae_Lactobacillus
f14141d03c23f3658014090c72c23866
Relative abundance
100.00%
10.00%
1.00%
0.10%
CU
pswab
vswab
Sample type
PID|visit
A1
A4
B1
B4
D1
D4
F1
G1
G4
H1
H4
I1
I4
J1
J4
L1
L4
M1
O1
O4

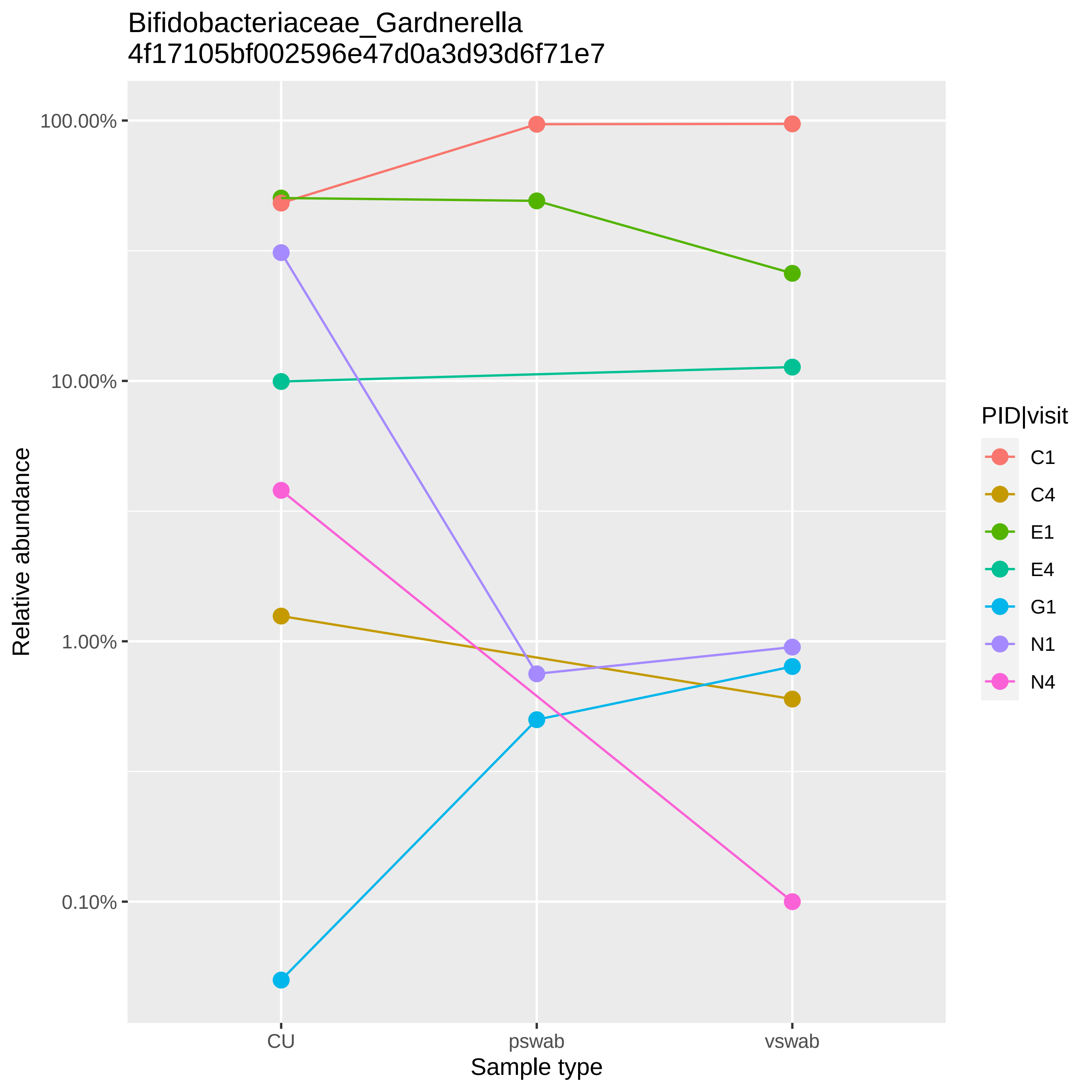
Bifidobacteriaceae_Gardnerella
4f17105bf002596e47d0a3d93d6f71e7
100.00%
10.00%
1.00%
0.10%
Relative abundance
CU
pswab
vswab
Sample type
PID|visit
C1
C4
E1
E4
G1
N1
N4

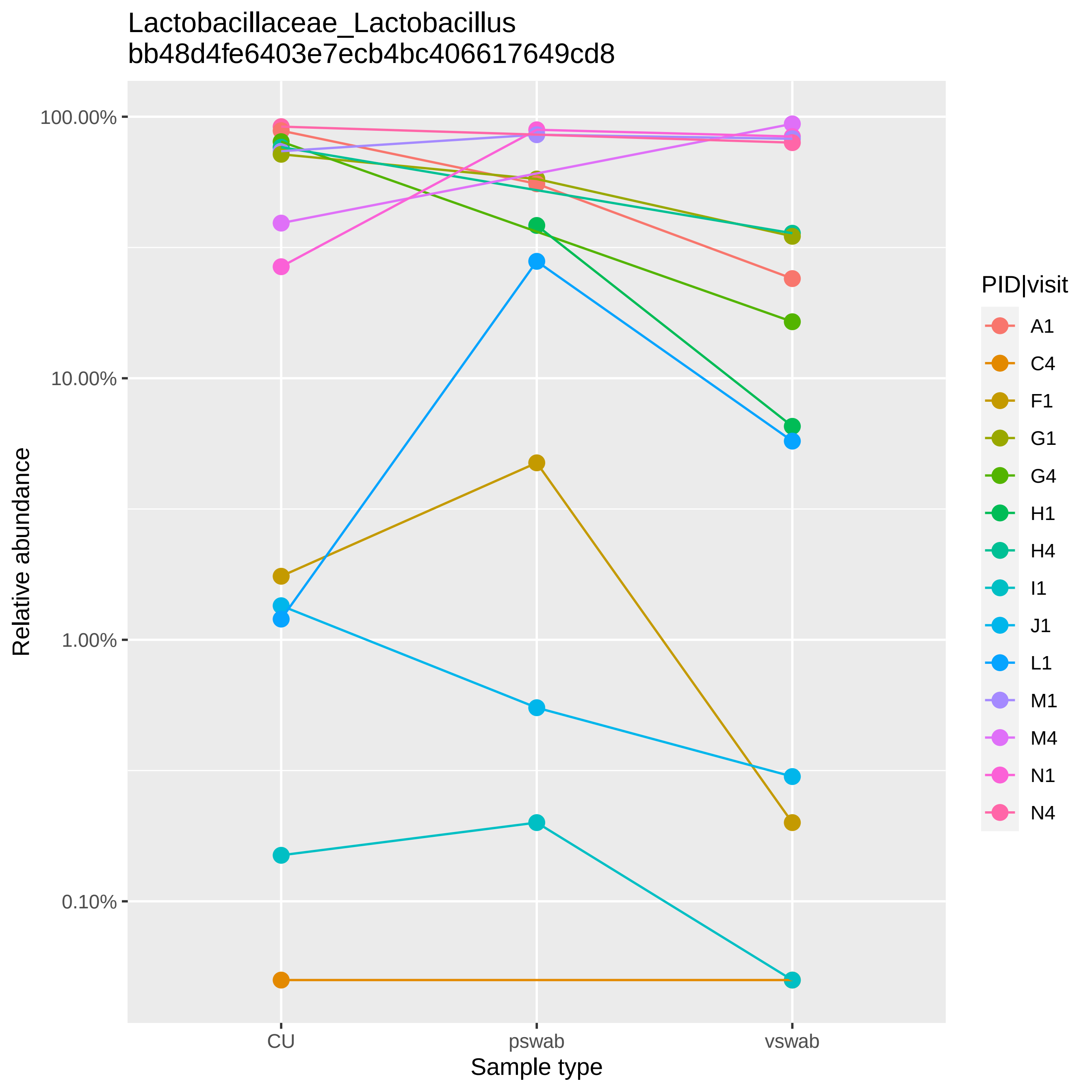
Lactobacillaceae_Lactobacillus
bb48d4fe6403e7ecb4bc406617649cd8
100.00%
10.00%
1.00%
0.10%
Relative abundance
CU
pswab
vswab
Sample type
PID|visit
A1
C4
F1
G1
G4
H1
H4
I1
J1
L1
M1
M4
N1
N4

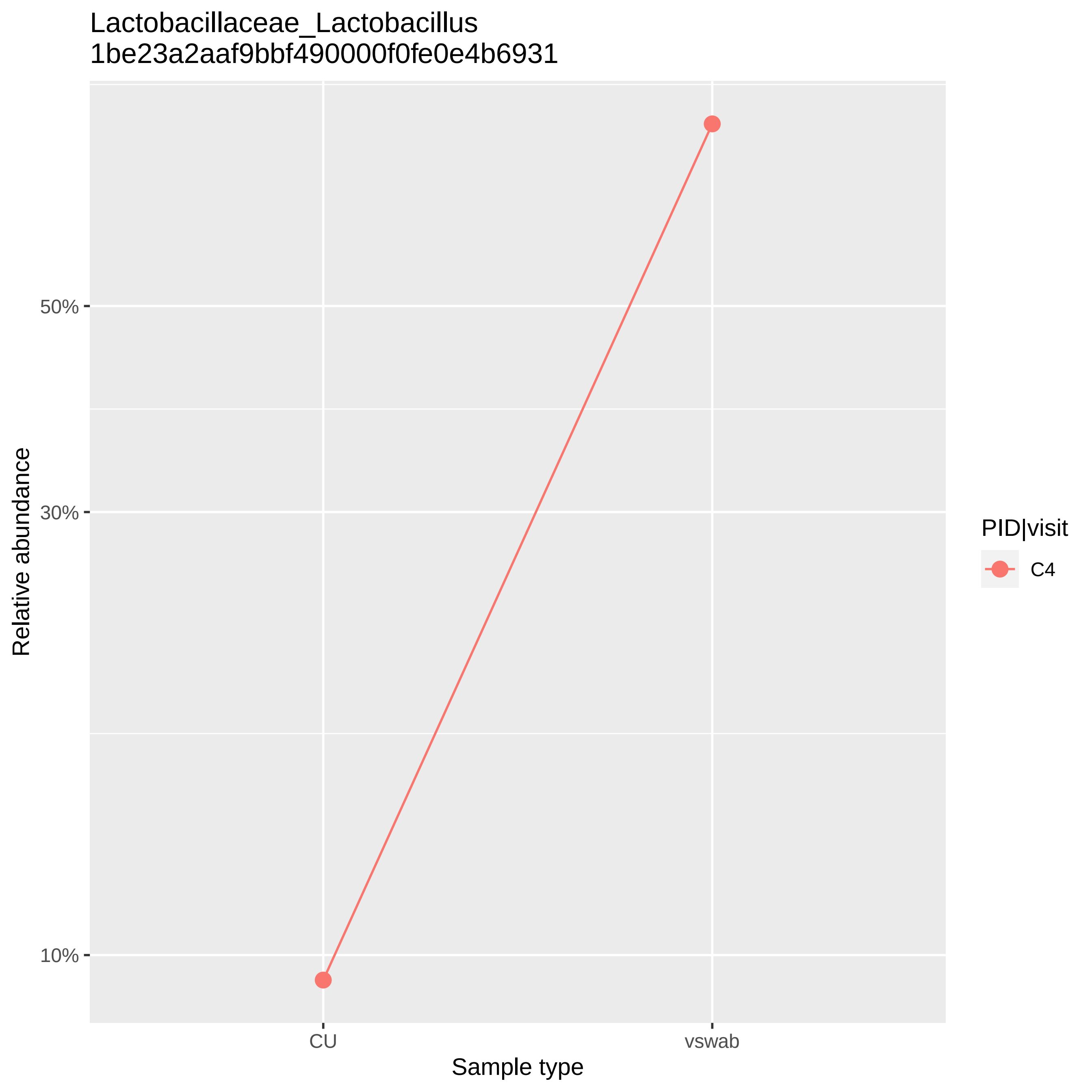

Lactobacillaceae_Lactobacillus
1be23a2aaf9bbf490000f0fe0e4b6931
50%
30%
10%
Relative abundance
CU
vswab
Sample type
PID|visit
C4

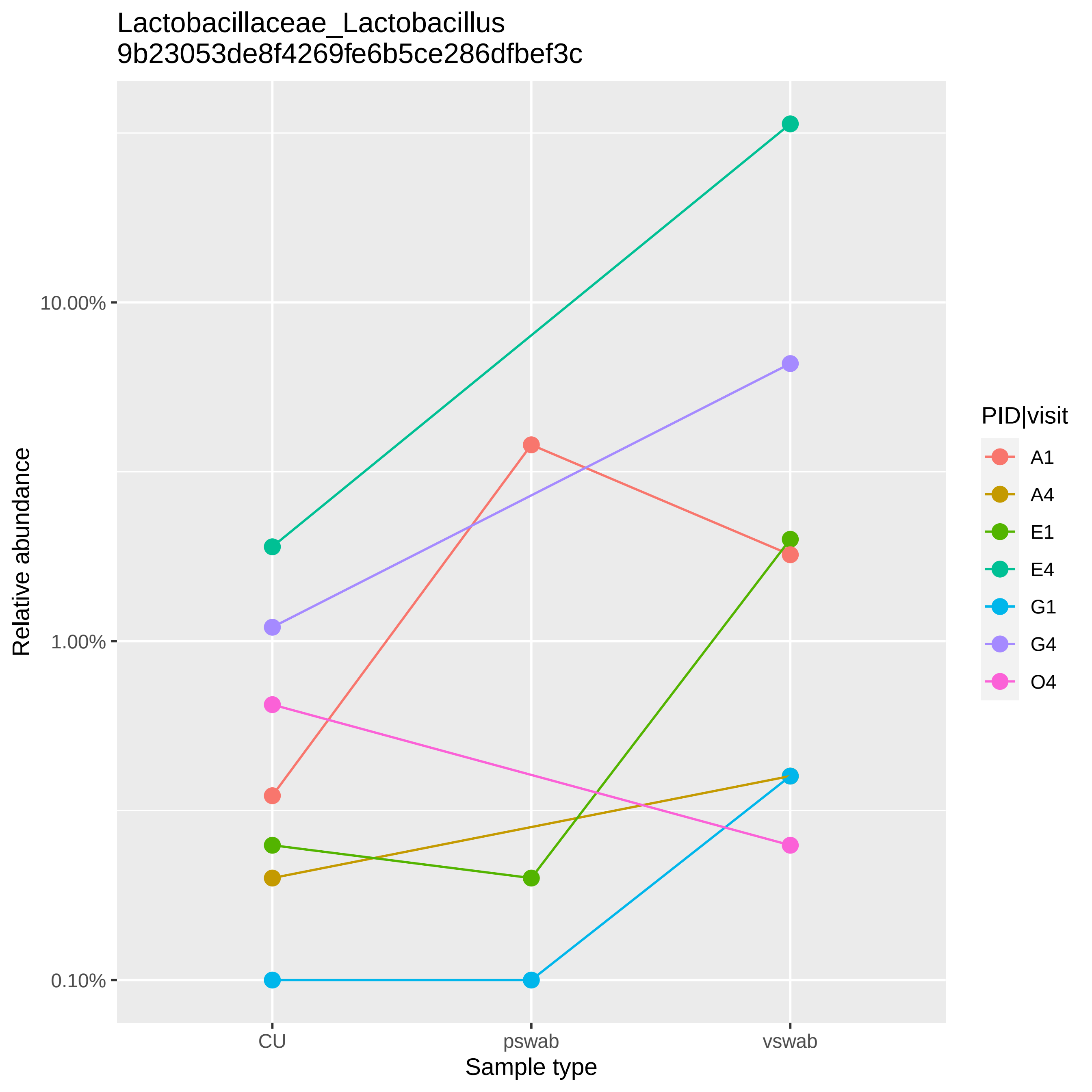

Lactobacillaceae_Lactobacillus
9b23053de8f4269fe6b5ce286dfbef3c
Relative abundance
10.00%
1.00%
0.10%
CU
pswab
vswab
Sample type
PID|visit
A1
A4
E1
E4
G1
G4
O4

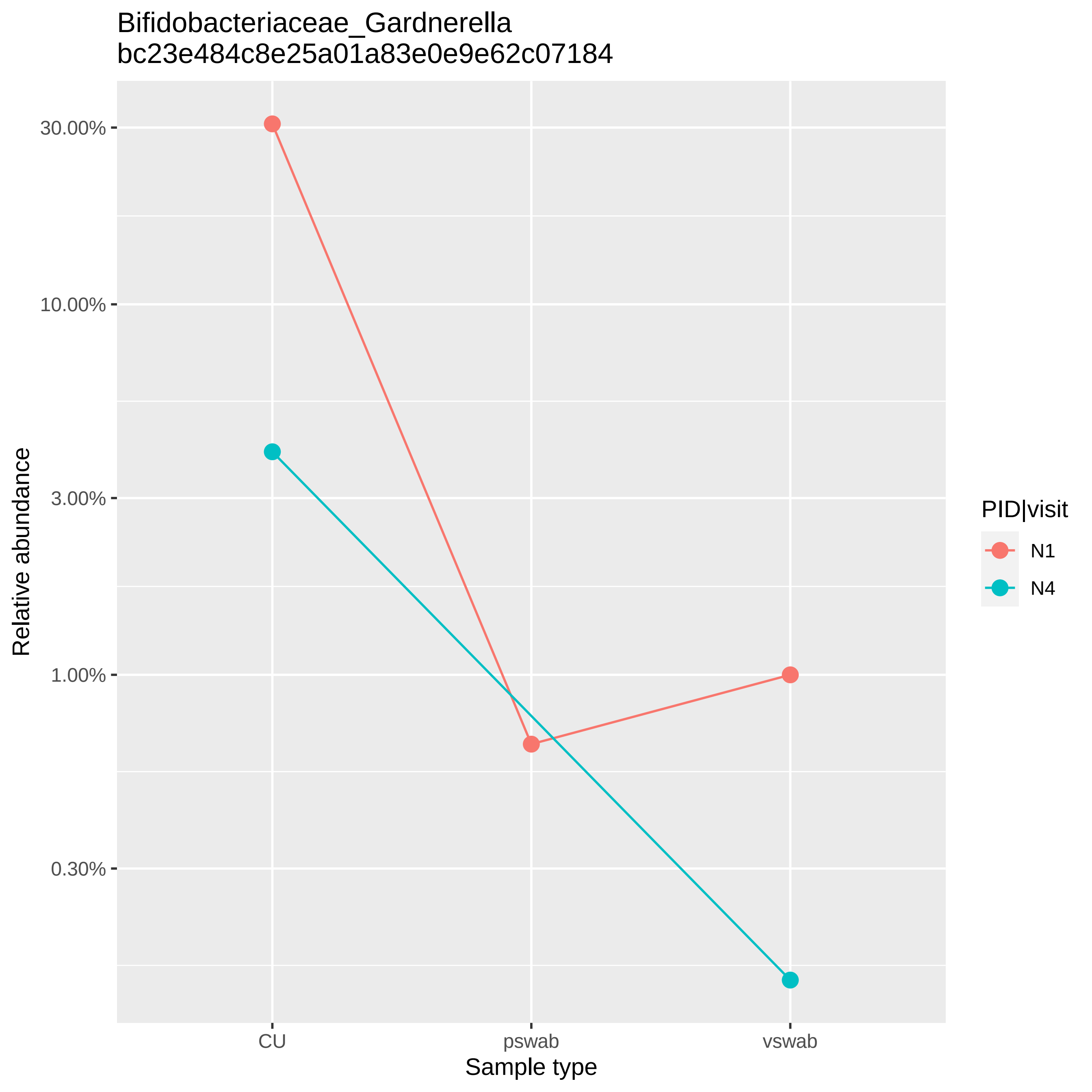

Bifidobacteriaceae_Gardnerella
bc23e484c8e25a01a83e0e9e62c07184
30.00%
10.00%
3.00%
1.00%
0.30%
Relative abundance
CU
pswab
vswab
Sample type
PID|visit
N1
N4

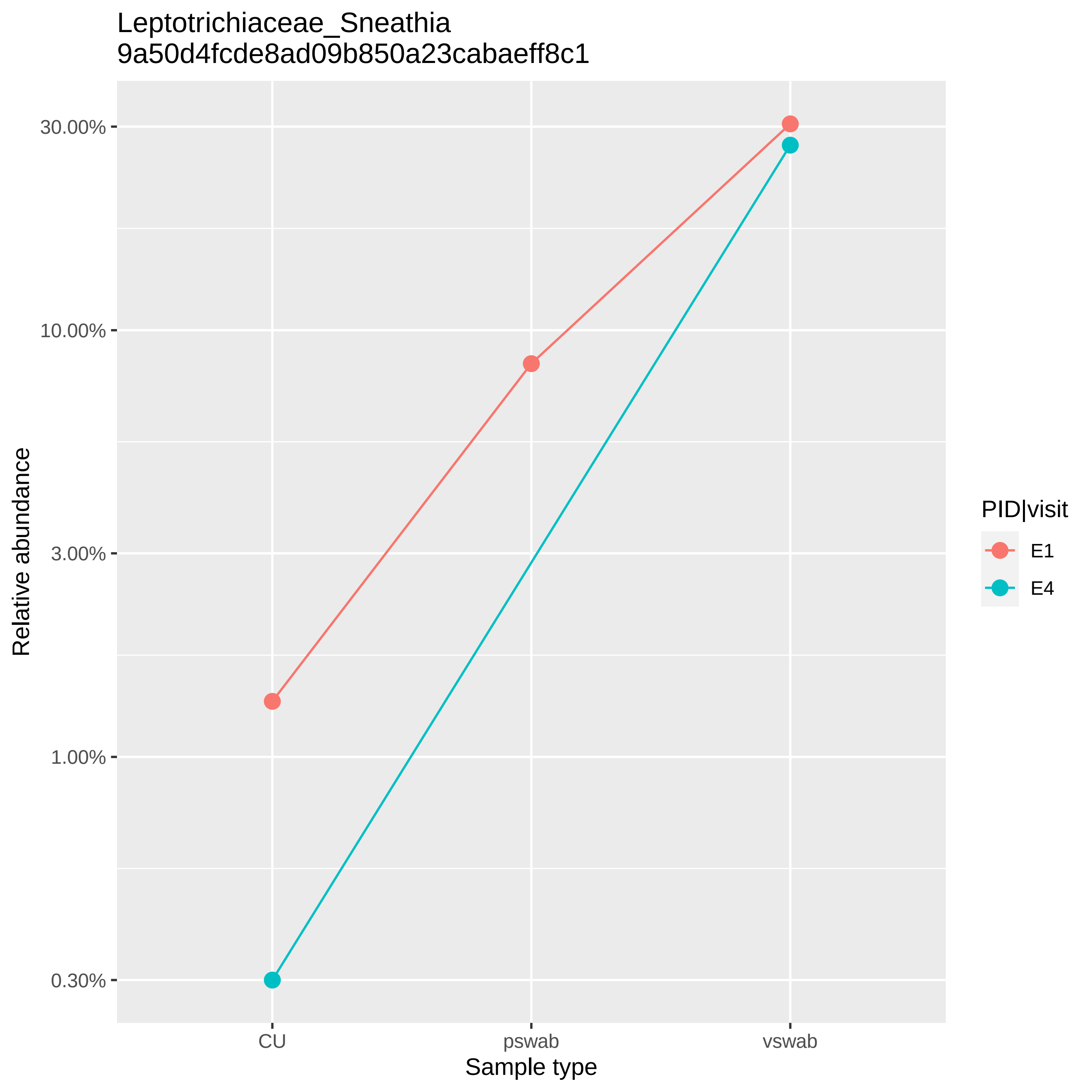
Leptotrichiaceae_Sneathia
9a50d4fcde8ad09b850a23cabaeff8c1
30.00%
10.00%
3.00%
1.00%
0.30%
Relative abundance
CU
pswab
vswab
Sample type
PID|visit
E1
E4

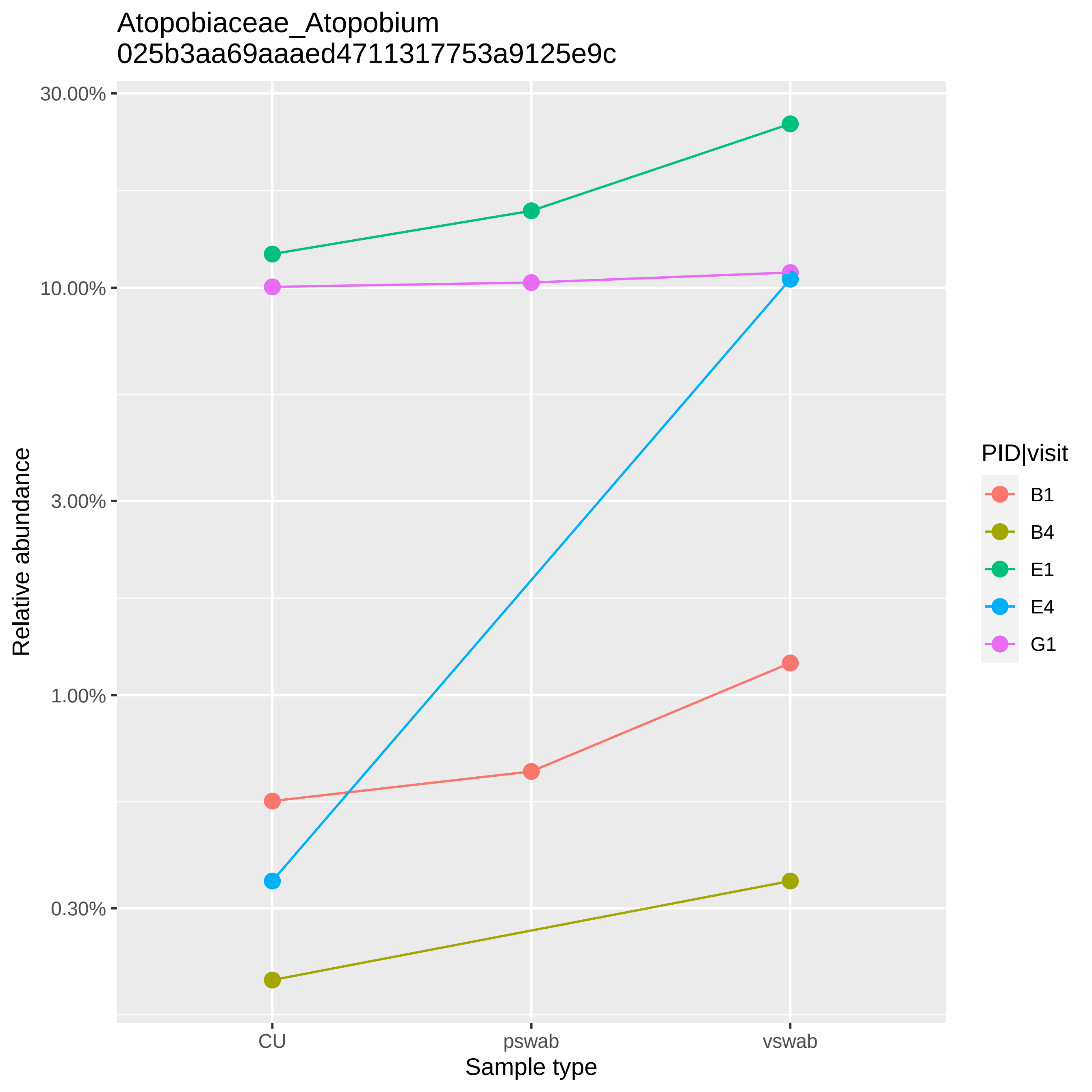
Atopobiaceae_Atopobium
025b3aa69aaaed4711317753a9125e9c
30.00%
10.00%
3.00%
1.00%
0.30%
Relative abundance
CU
pswab
vswab
Sample type
PID|visit
B1
B4
E1
E4
G1

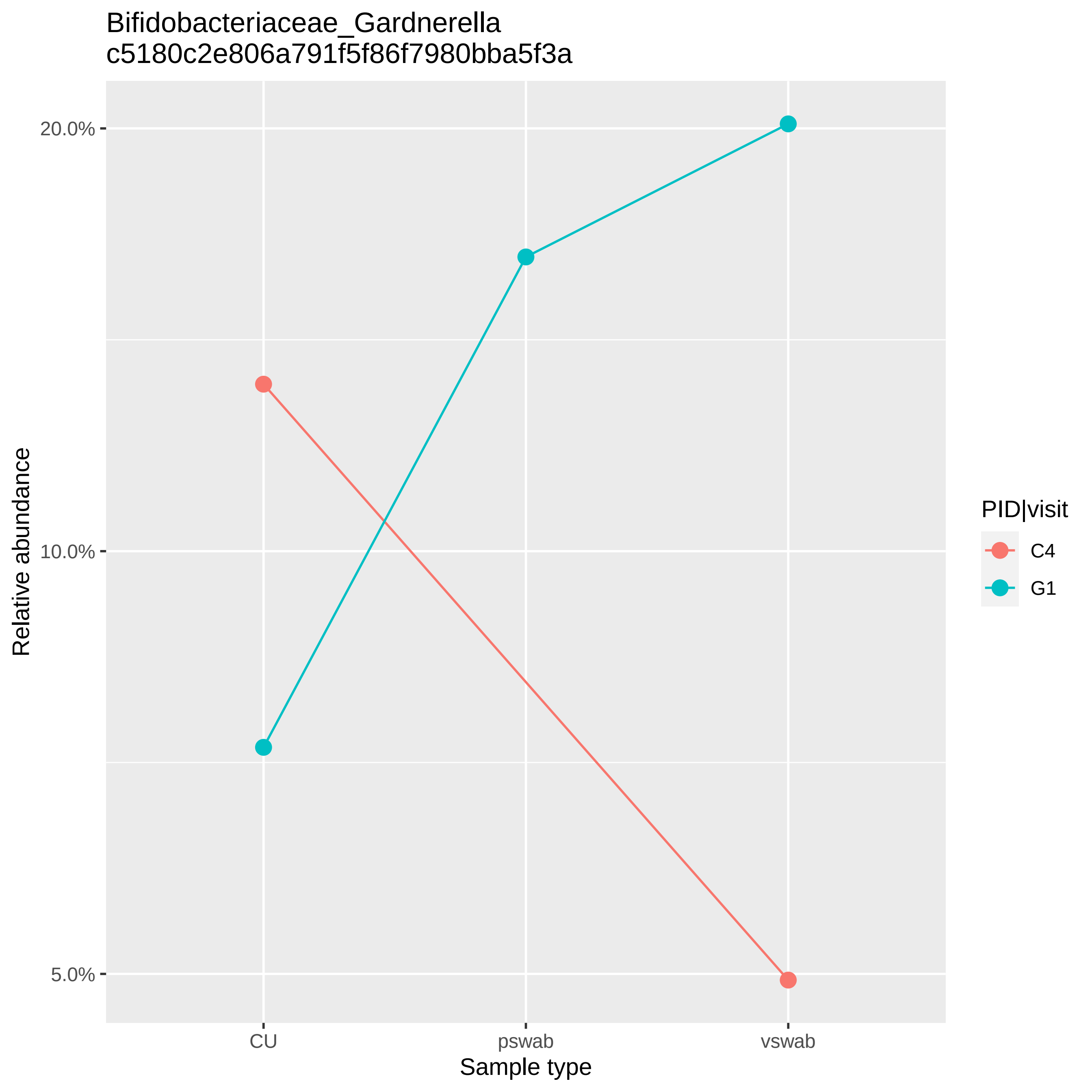
Bifidobacteriaceae_Gardnerella
c5180c2e806a791f5f86f7980bba5f3a
20.0%
10.0%
5.0%
Relative abundance
CU
pswab
vswab
Sample type
PID|visit
C4
G1

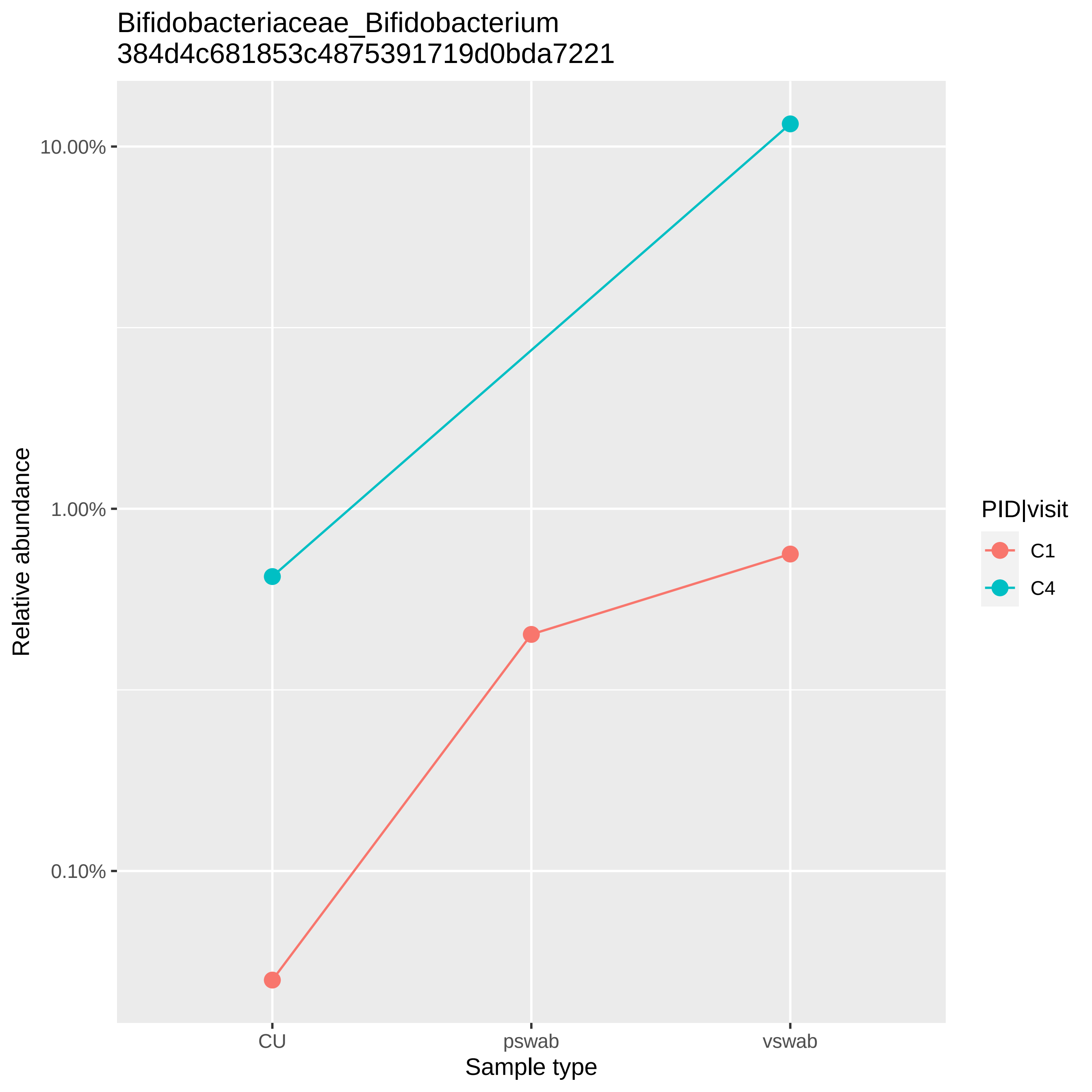

Bifidobacteriaceae_Bifidobacterium
384d4c681853c4875391719d0bda7221
Relative abundance
10.00%
1.00%
0.10%
CU
pswab
vswab
Sample type
PID|visit
C1
C4

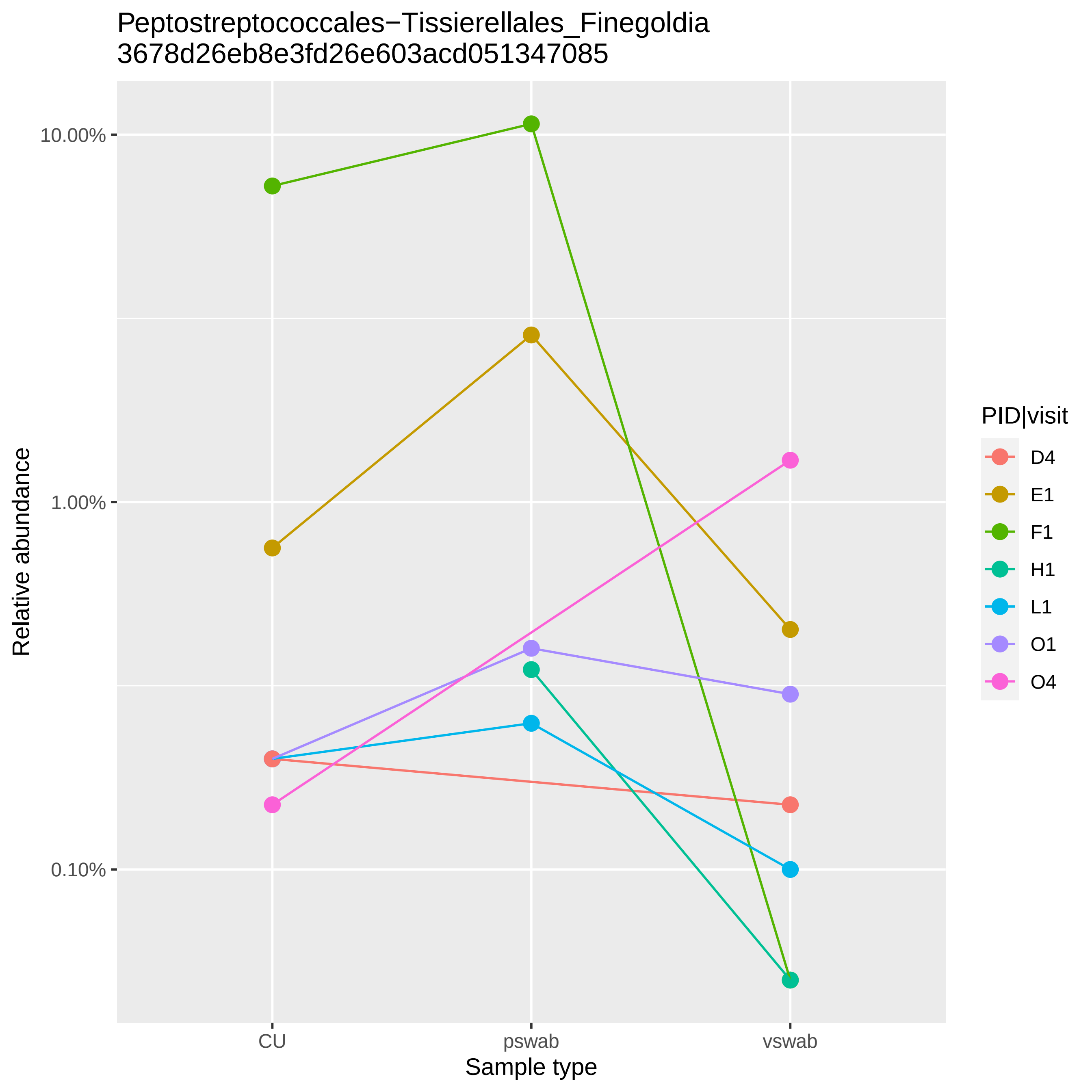

Peptostreptococcales−Tissierellales_Finegoldia
3678d26eb8e3fd26e603acd051347085
10.00%
1.00%
0.10%
Relative abundance
CU
pswab
vswab
Sample type
PID|visit
D4
E1
F1
H1
L1
O1
O4

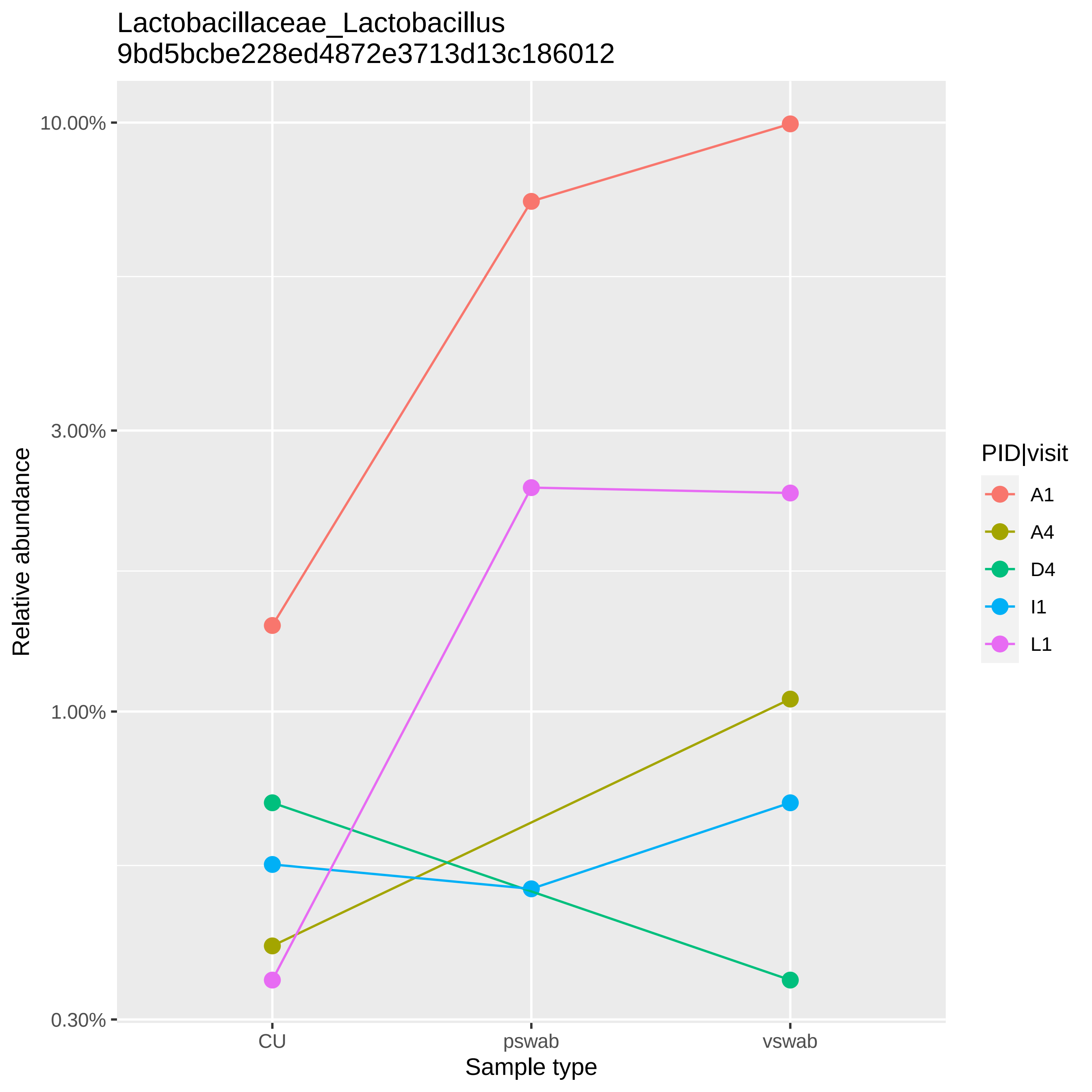
Lactobacillaceae_Lactobacillus
9bd5bcbe228ed4872e3713d13c186012
10.00%
3.00%
1.00%
0.30%
Relative abundance
CU
pswab
vswab
Sample type
PID|visit
A1
A4
D4
I1
L1

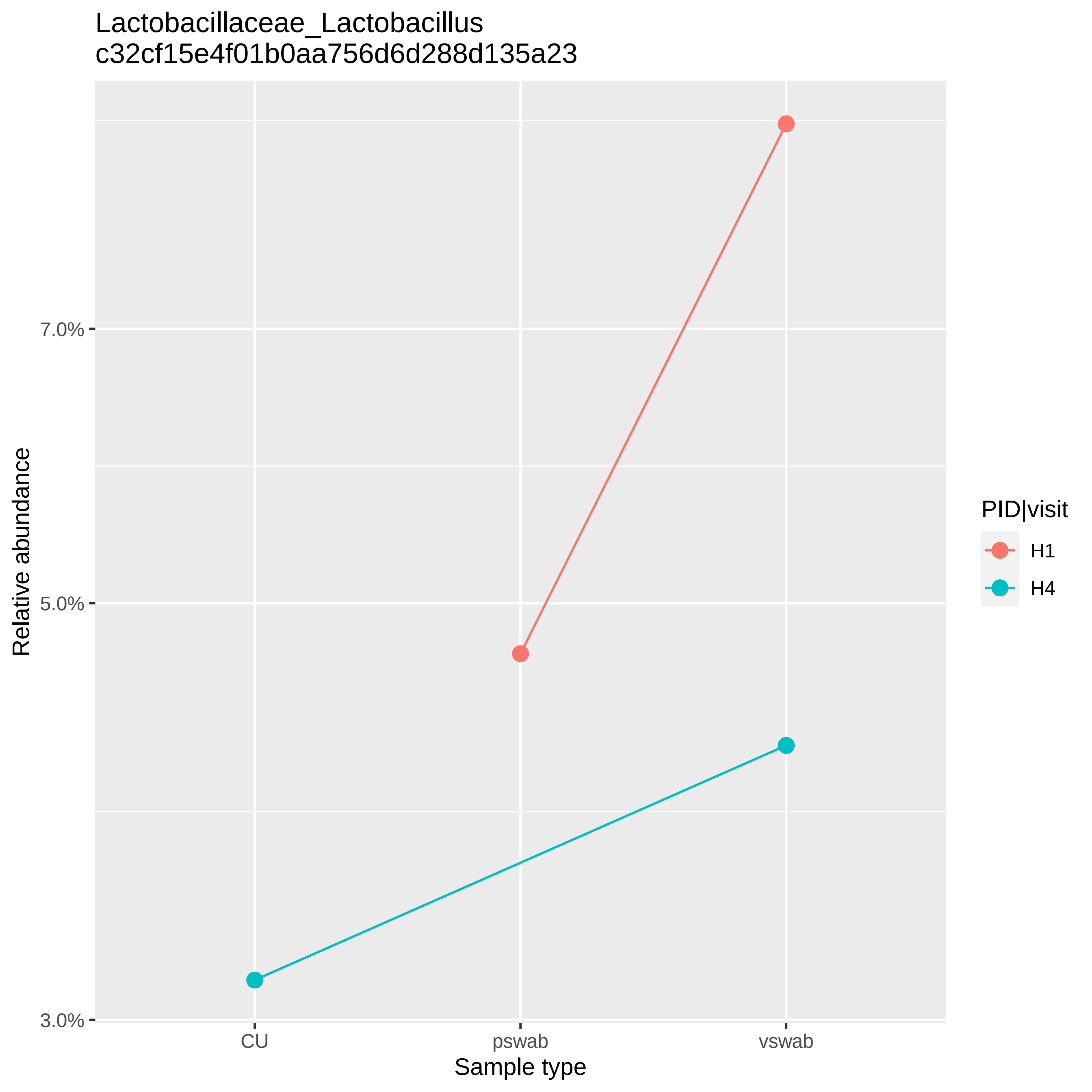
Lactobacillaceae_Lactobacillus
c32cf15e4f01b0aa756d6d288d135a23
7.0%
5.0%
3.0%
Relative abundance
CU
pswab
vswab
Sample type
PID|visit
H1
H4

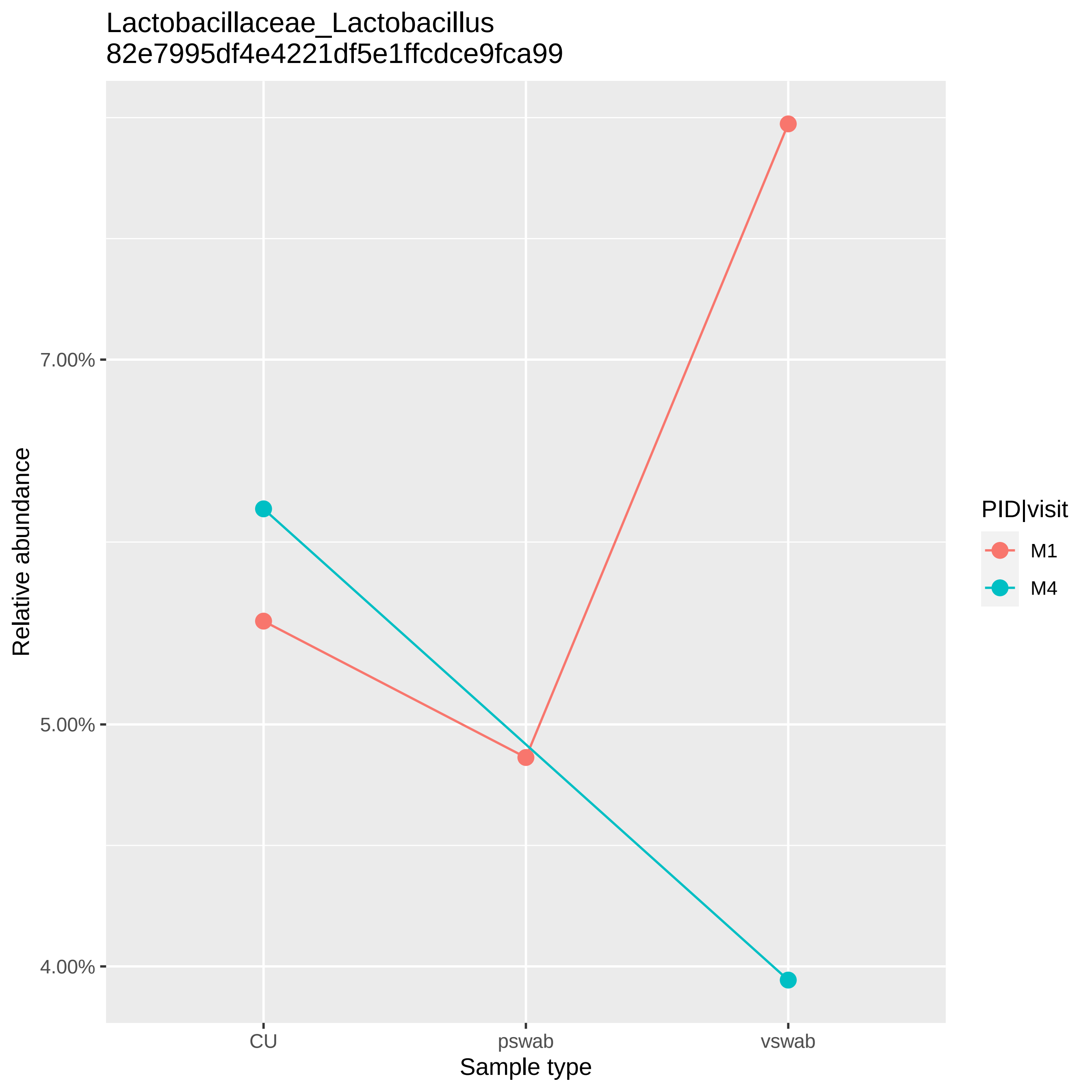

Lactobacillaceae_Lactobacillus
82e7995df4e4221df5e1ffcdce9fca99
7.00%
5.00%
4.00%
Relative abundance
CU
pswab
vswab
Sample type
PID|visit
M1
M4

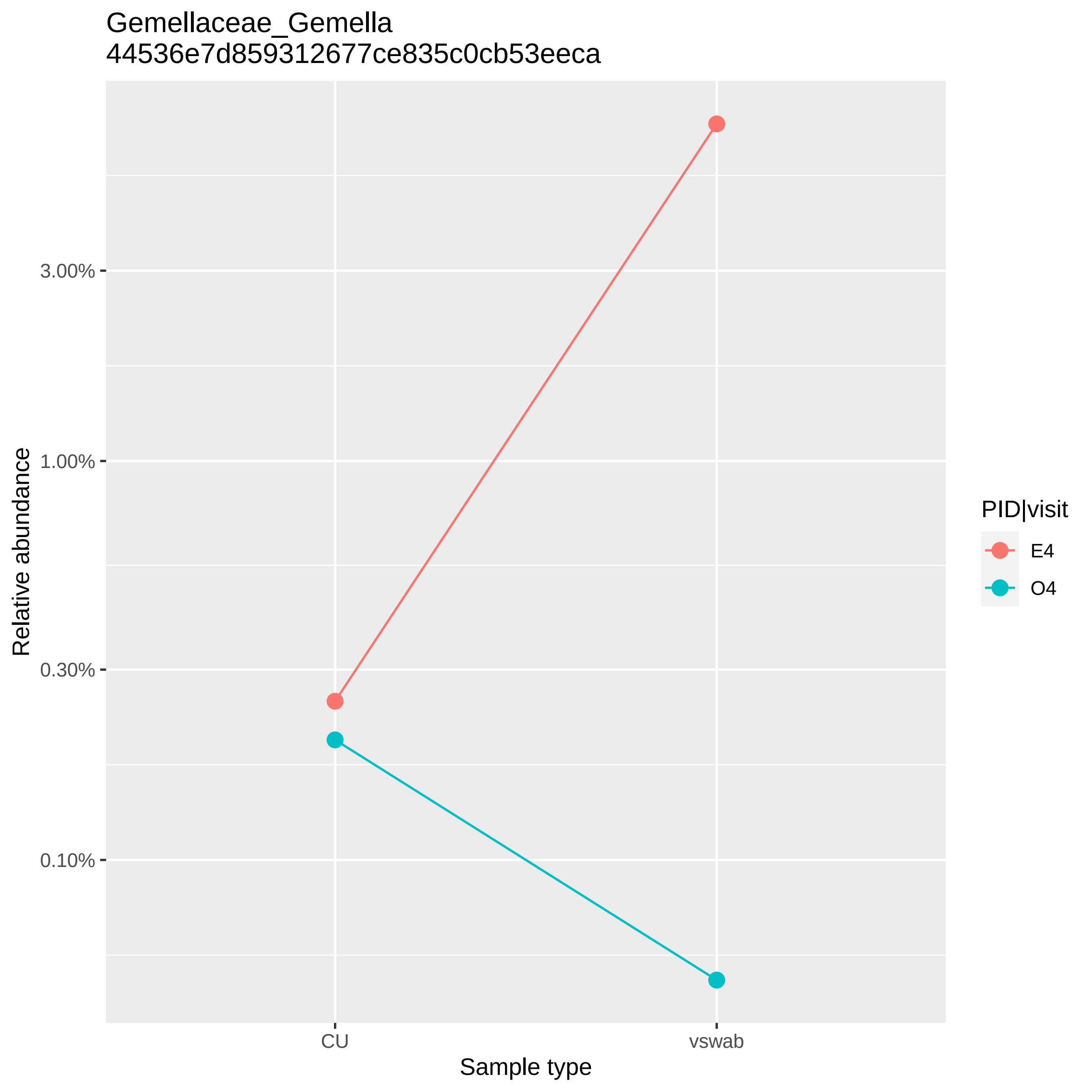

Gemellaceae_Gemella
44536e7d859312677ce835c0cb53eeca
3.00%
1.00%
0.30%
0.10%
Relative abundance
CU
vswab
Sample type
PID|visit
E4
O4

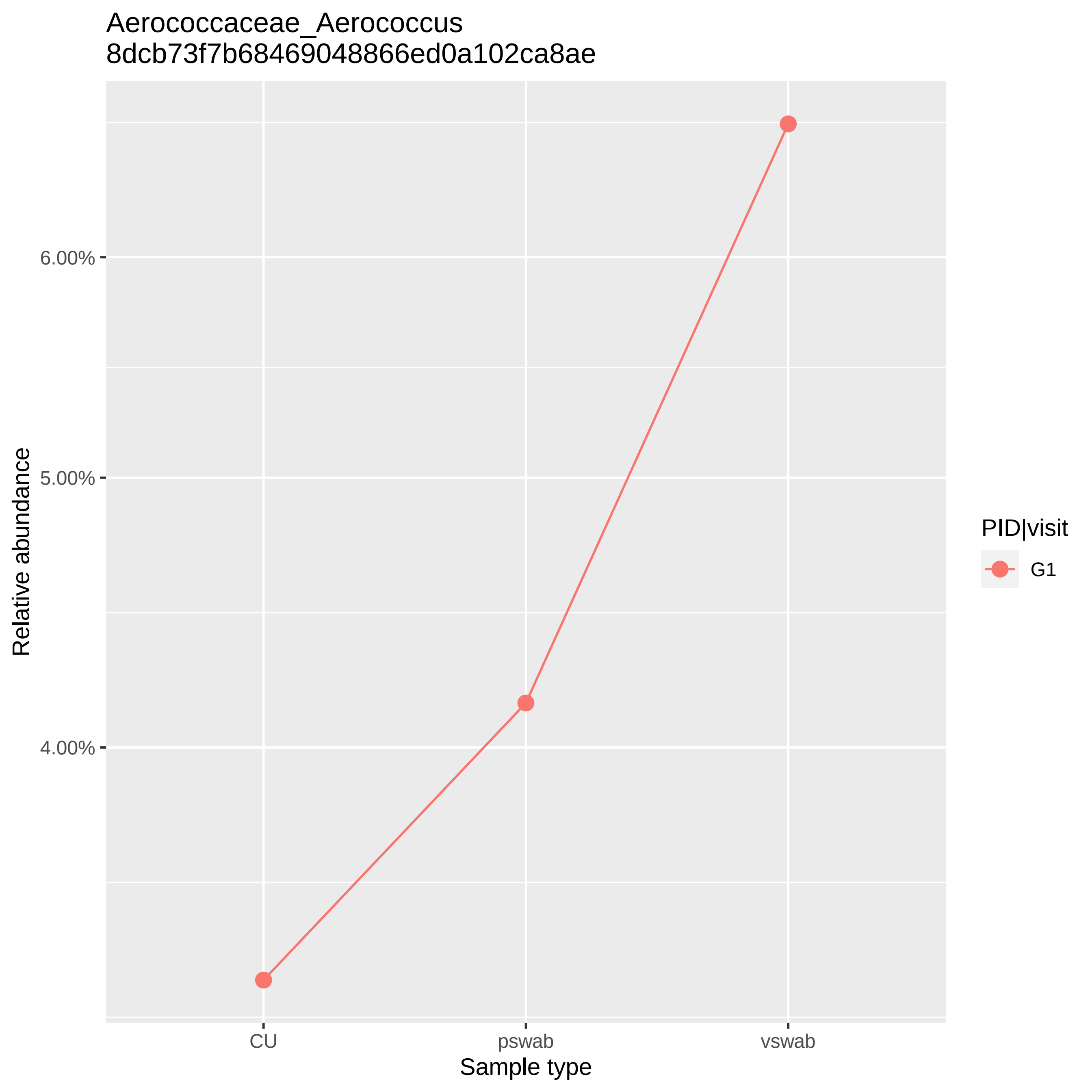

Aerococcaceae_Aerococcus
8dcb73f7b68469048866ed0a102ca8ae
6.00%
5.00%
4.00%
Relative abundance
CU
pswab
vswab
Sample type
PID|visit
G1

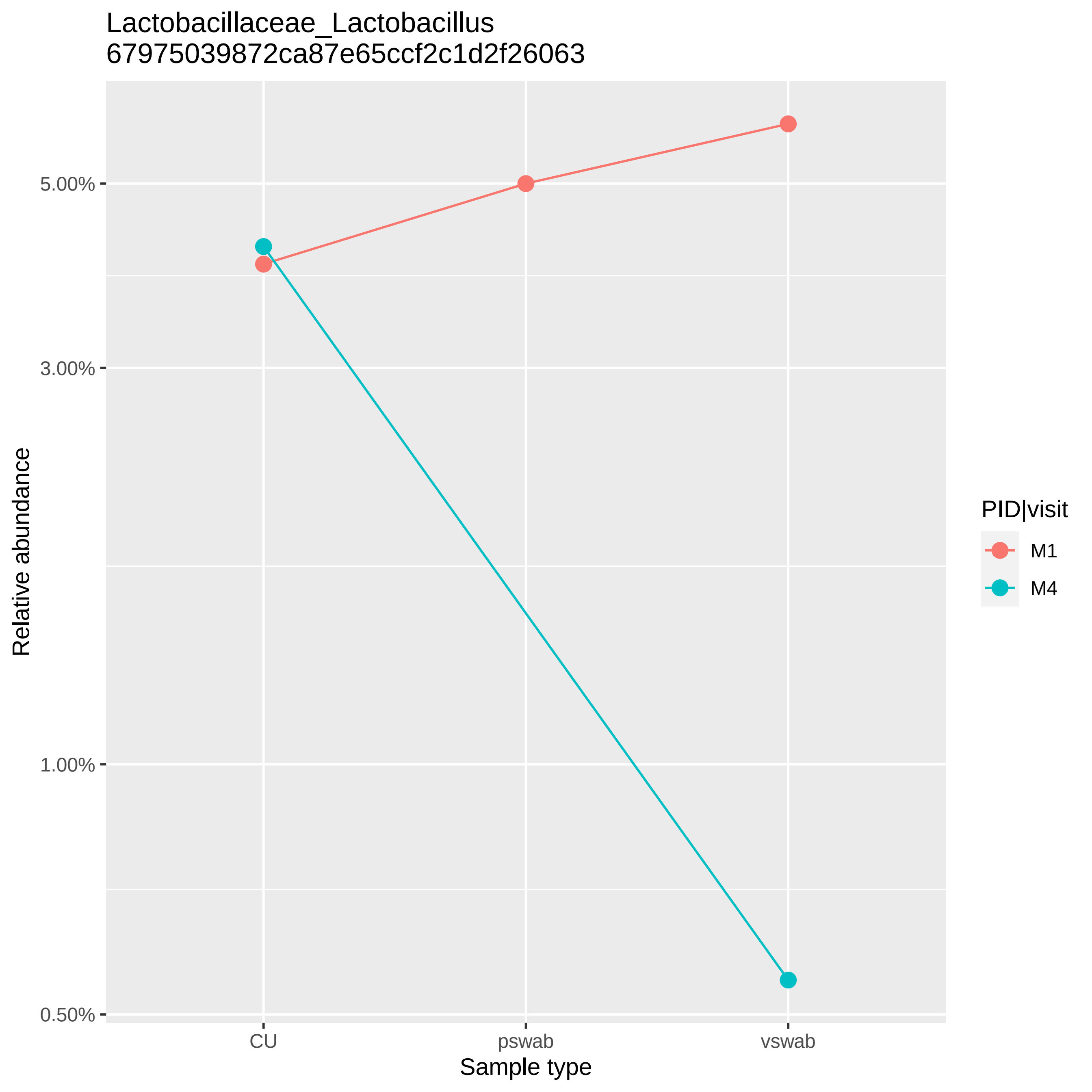

Lactobacillaceae_Lactobacillus
67975039872ca87e65ccf2c1d2f26063
5.00%
3.00%
1.00%
0.50%
Relative abundance
CU
pswab
vswab
Sample type
PID|visit
M1
M4

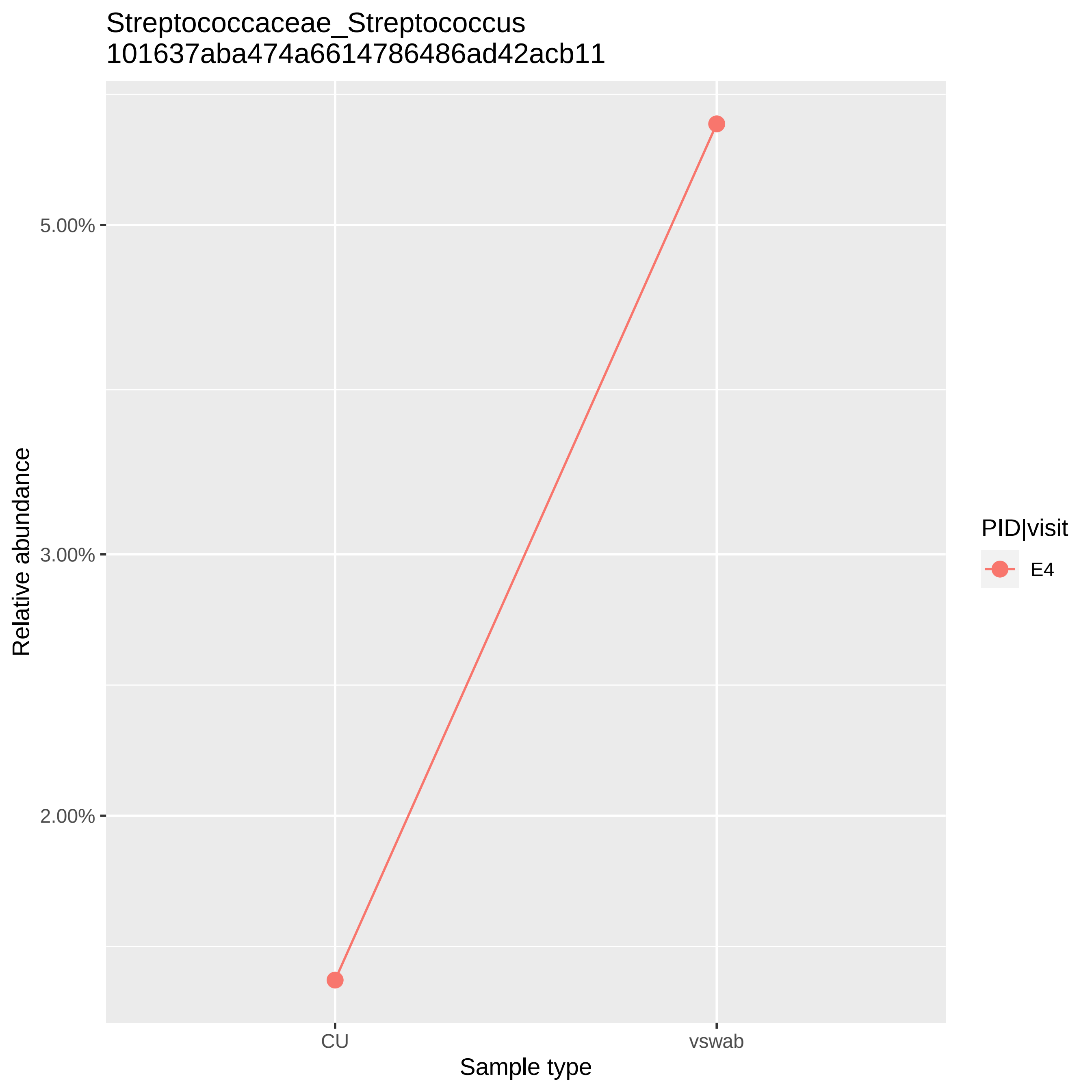

Streptococcaceae_Streptococcus
101637aba474a6614786486ad42acb11
Relative abundance
5.00%
3.00%
2.00%
CU
vswab
Sample type
PID|visit
E4

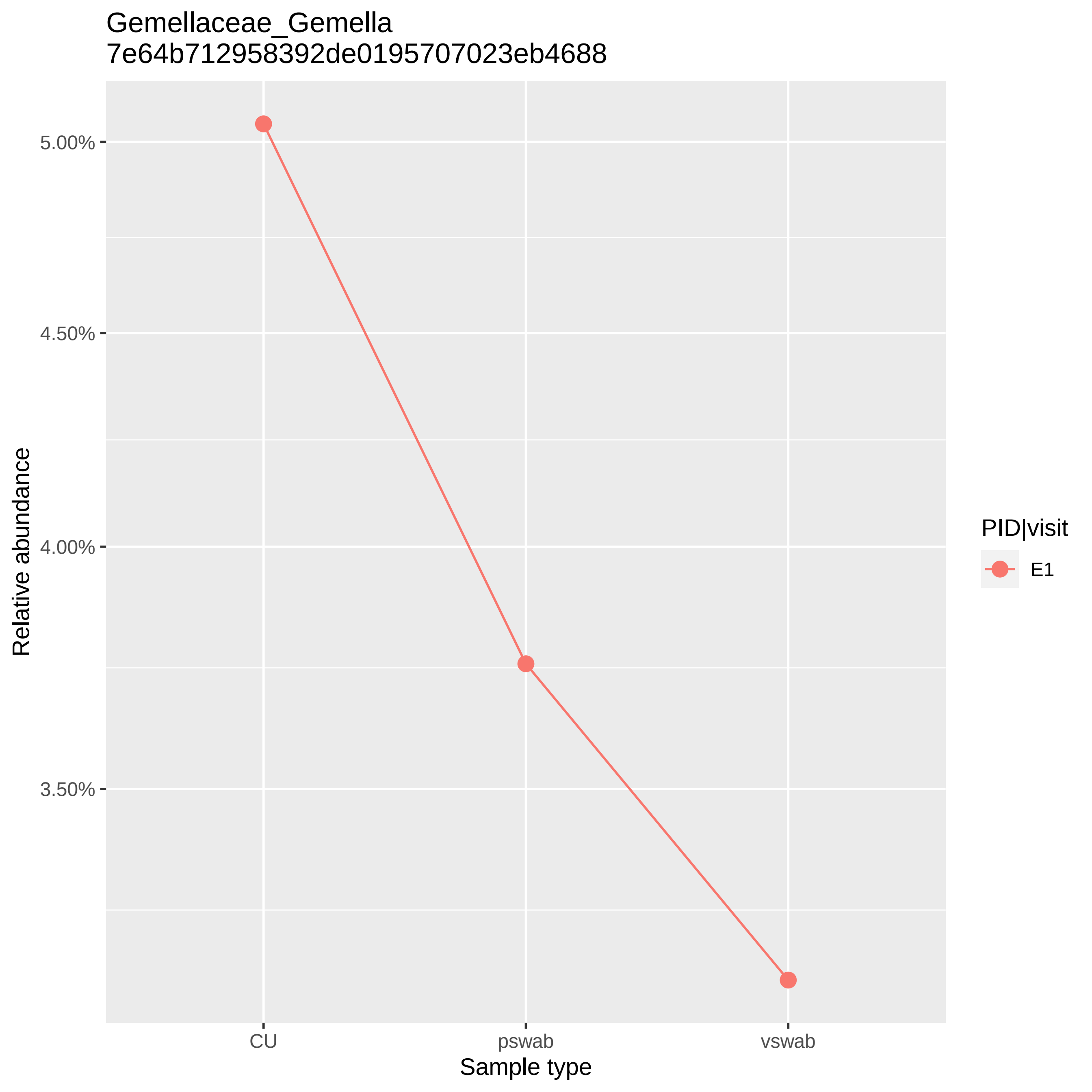
Gemellaceae_Gemella
7e64b712958392de0195707023eb4688
Relative abundance
5.00%
4.50%
4.00%
3.50%
CU
pswab
vswab
Sample type
PID|visit
E1

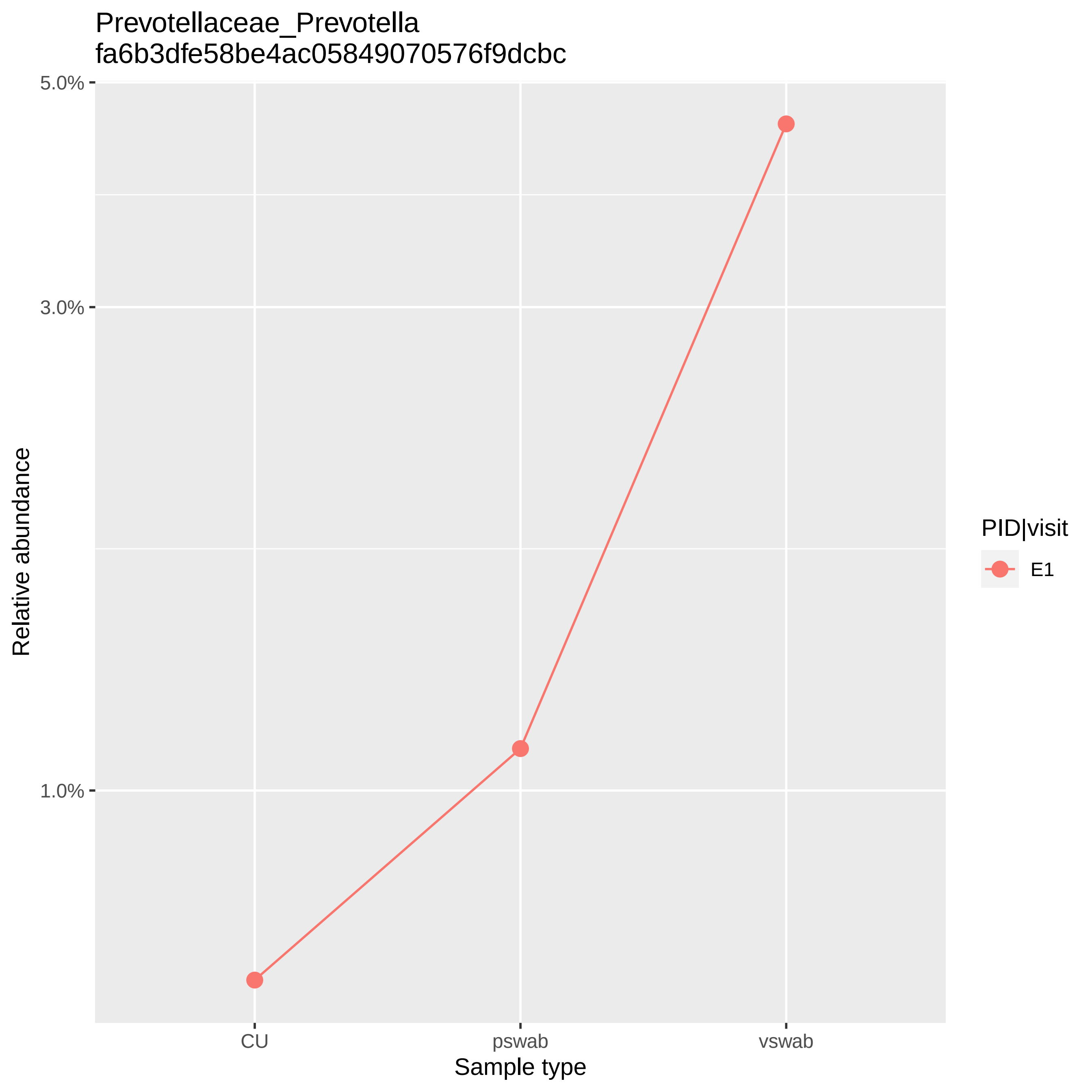
Prevotellaceae_Prevotella
fa6b3dfe58be4ac05849070576f9dcbc
5.0%
3.0%
1.0%
Relative abundance
CU
pswab
vswab
Sample type
PID|visit
E1

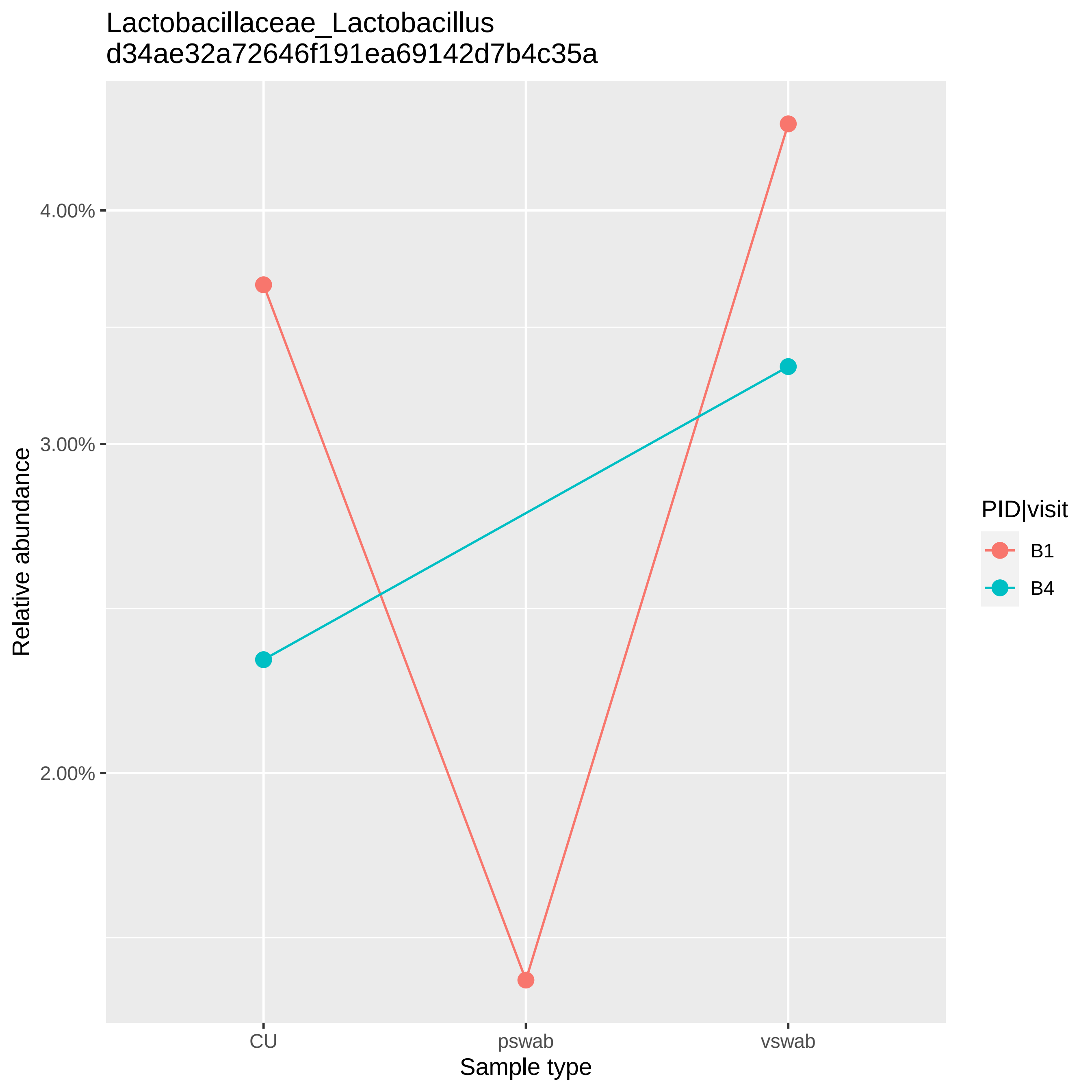

Lactobacillaceae_Lactobacillus
d34ae32a72646f191ea69142d7b4c35a
4.00%
3.00%
2.00%
Relative abundance
CU
pswab
vswab
Sample type
PID|visit
B1
B4

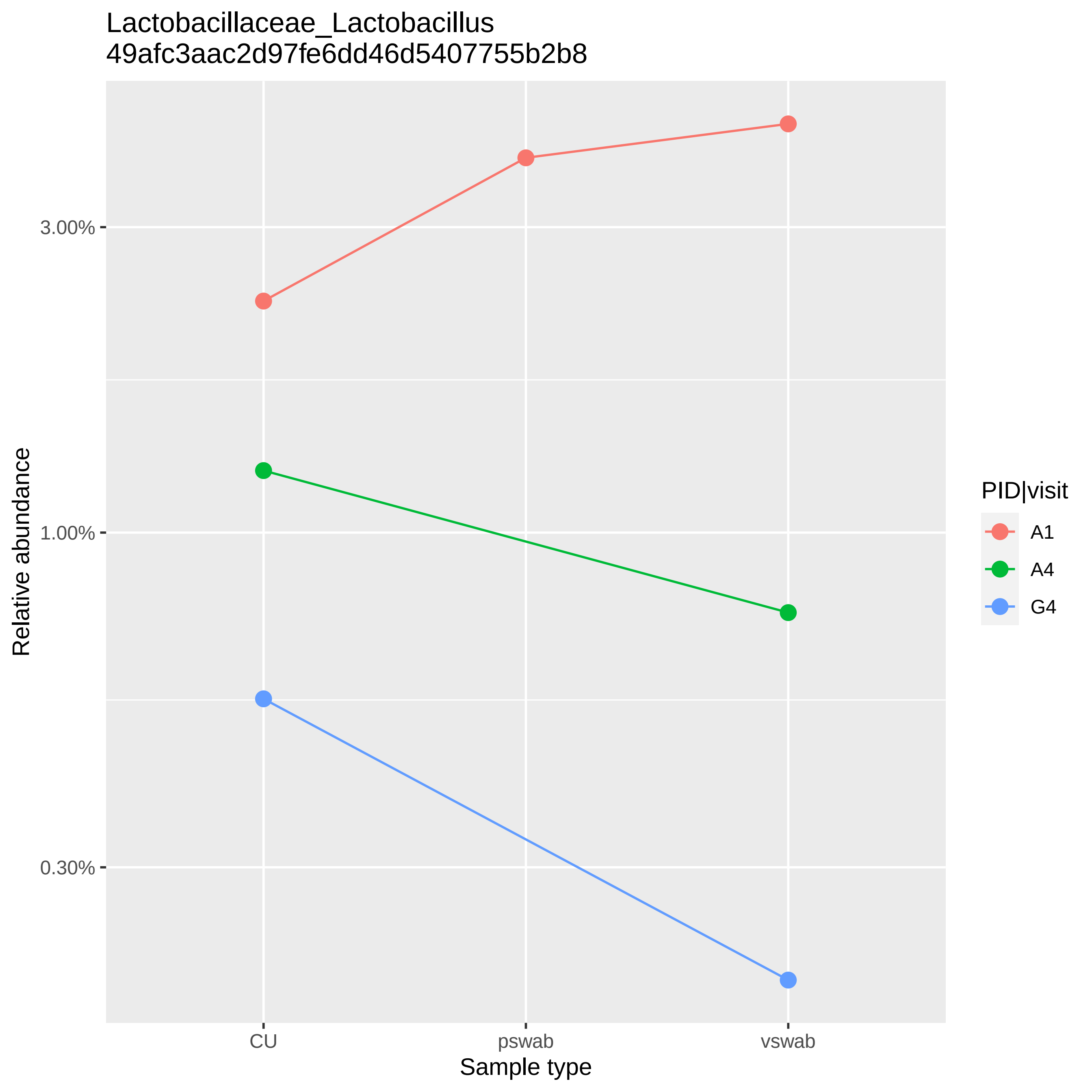

Lactobacillaceae_Lactobacillus
49afc3aac2d97fe6dd46d5407755b2b8
3.00%
1.00%
0.30%
Relative abundance
CU
pswab
vswab
Sample type
PID|visit
A1
A4
G4

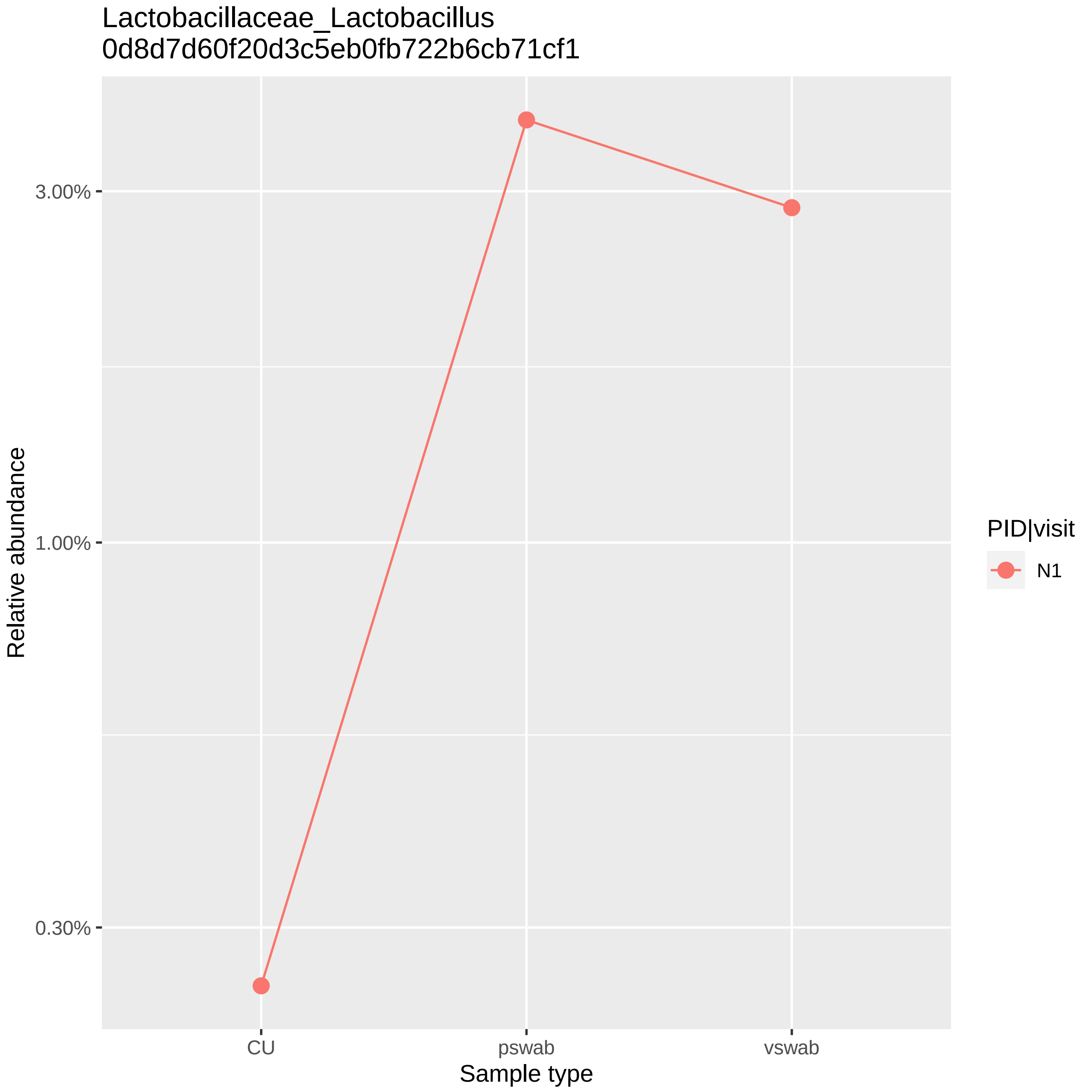
Lactobacillaceae_Lactobacillus
0d8d7d60f20d3c5eb0fb722b6cb71cf1
Relative abundance
3.00%
1.00%
0.30%
CU
pswab
vswab
Sample type
PID|visit
N1

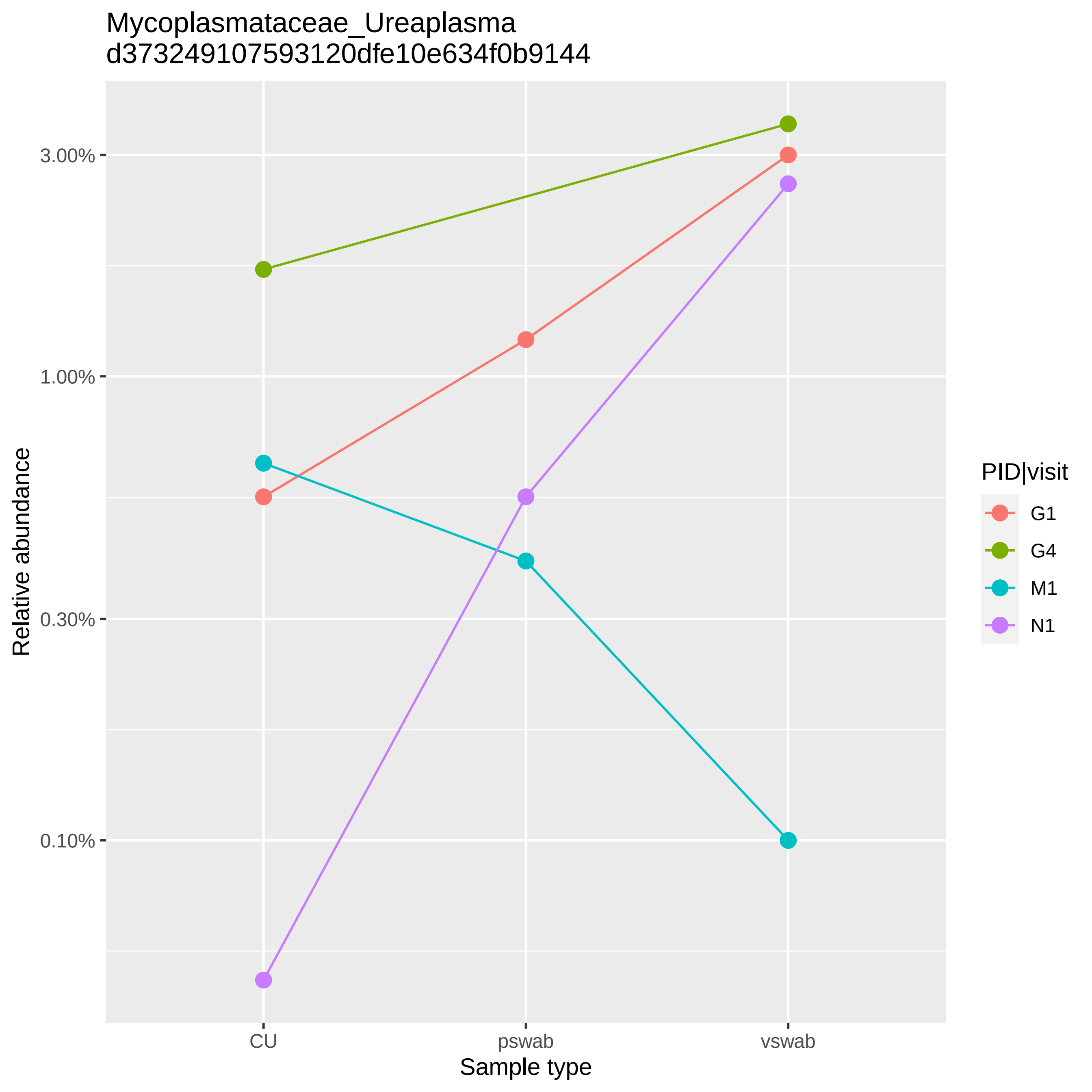
Mycoplasmataceae_Ureaplasma
d37324910759312Odfe10e634f0b9144
3.00%
1.00%
0.30%
0.10%
Relative abundance
CU
pswab
vswab
Sample type
PID|visit
G1
G4
M1
N1

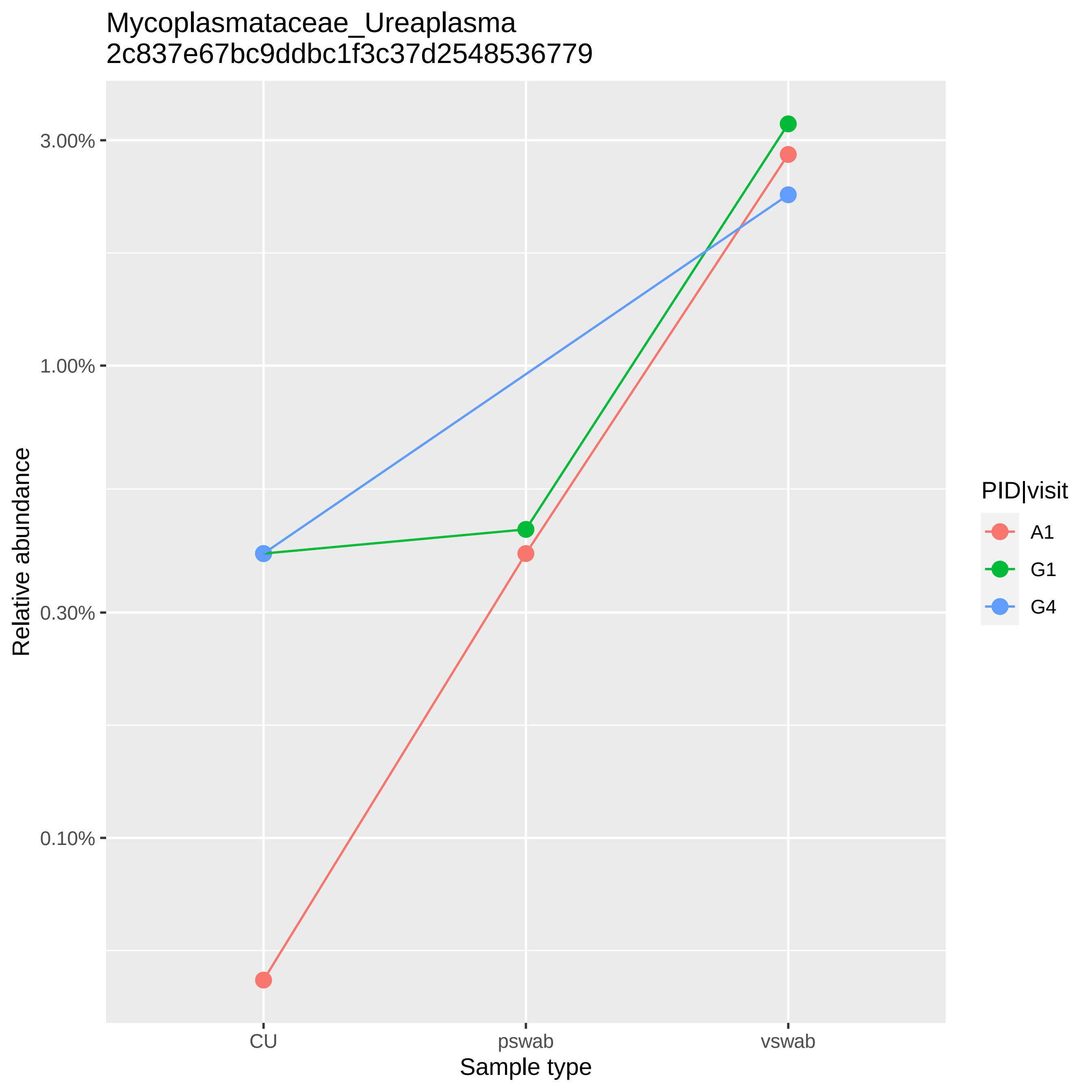

Mycoplasmataceae_Ureaplasma
2c837e67bc9ddbc1f3c37d2548536779
3.00%
1.00%
0.30%
0.10%
Relative abundance
CU
pswab
vswab
Sample type
PID|visit
A1
G1
G4

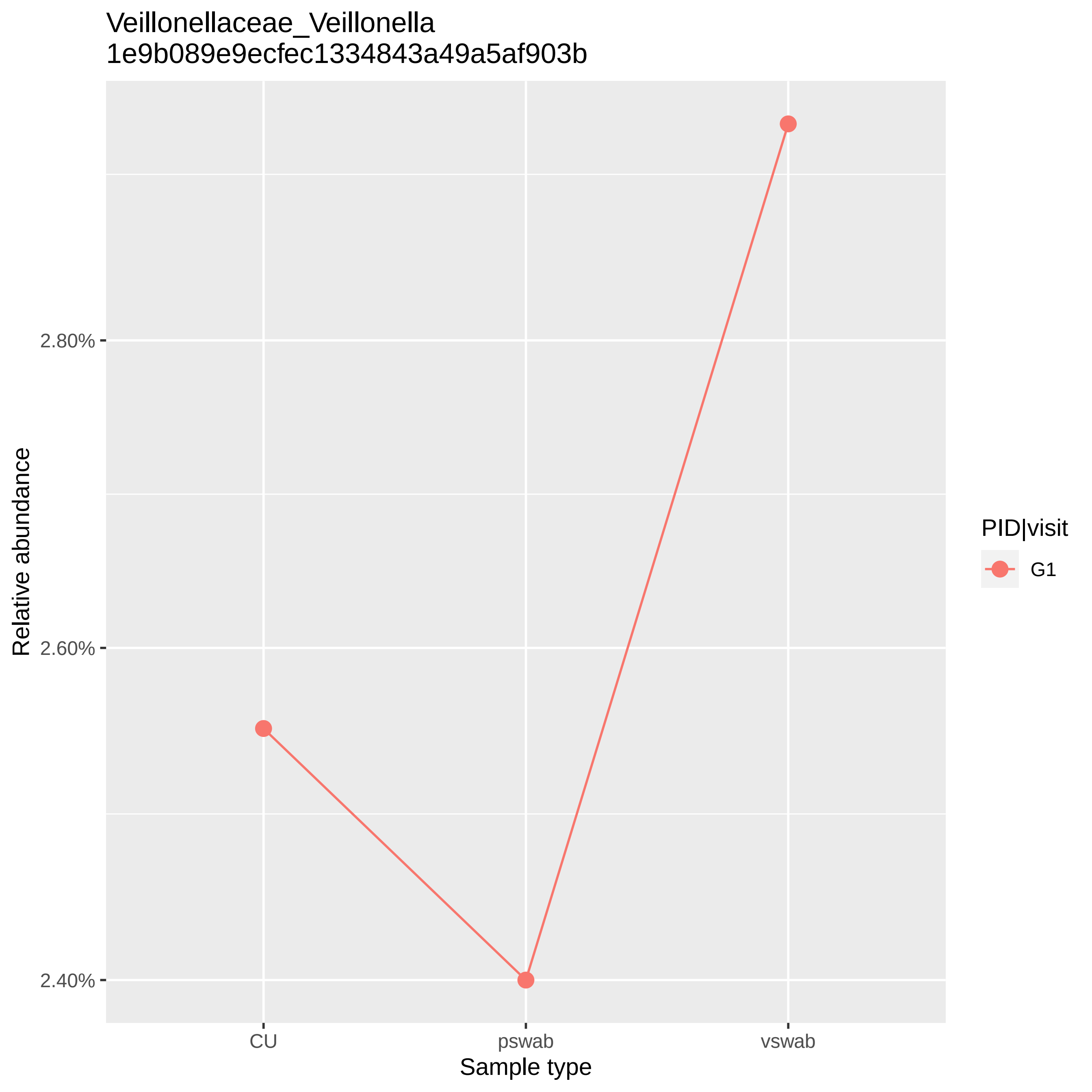

Veillonellaceae_Veillonella
1e9b089e9ecfec1334843a49a5af903b
Relative abundance
2.80%
2.60%
2.40%
CU
pswab
vswab
Sample type
PID|visit
G1

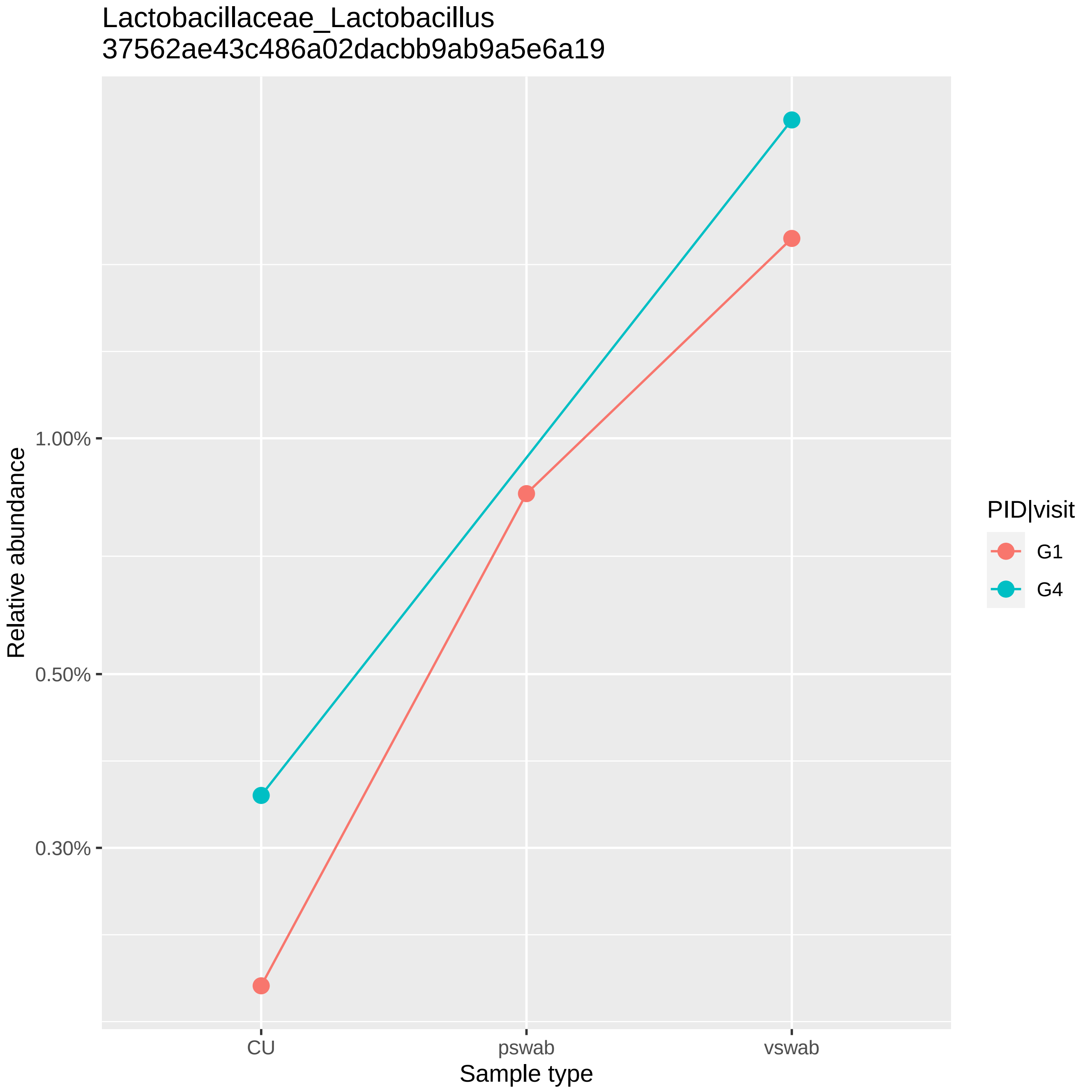

Lactobacillaceae_Lactobacillus
37562ae43c486a02dacbb9ab9a5e6a19
Relative abundance
1.00%
0.50%
0.30%
CU
pswab
vswab
Sample type
PID|visit
G1
G4

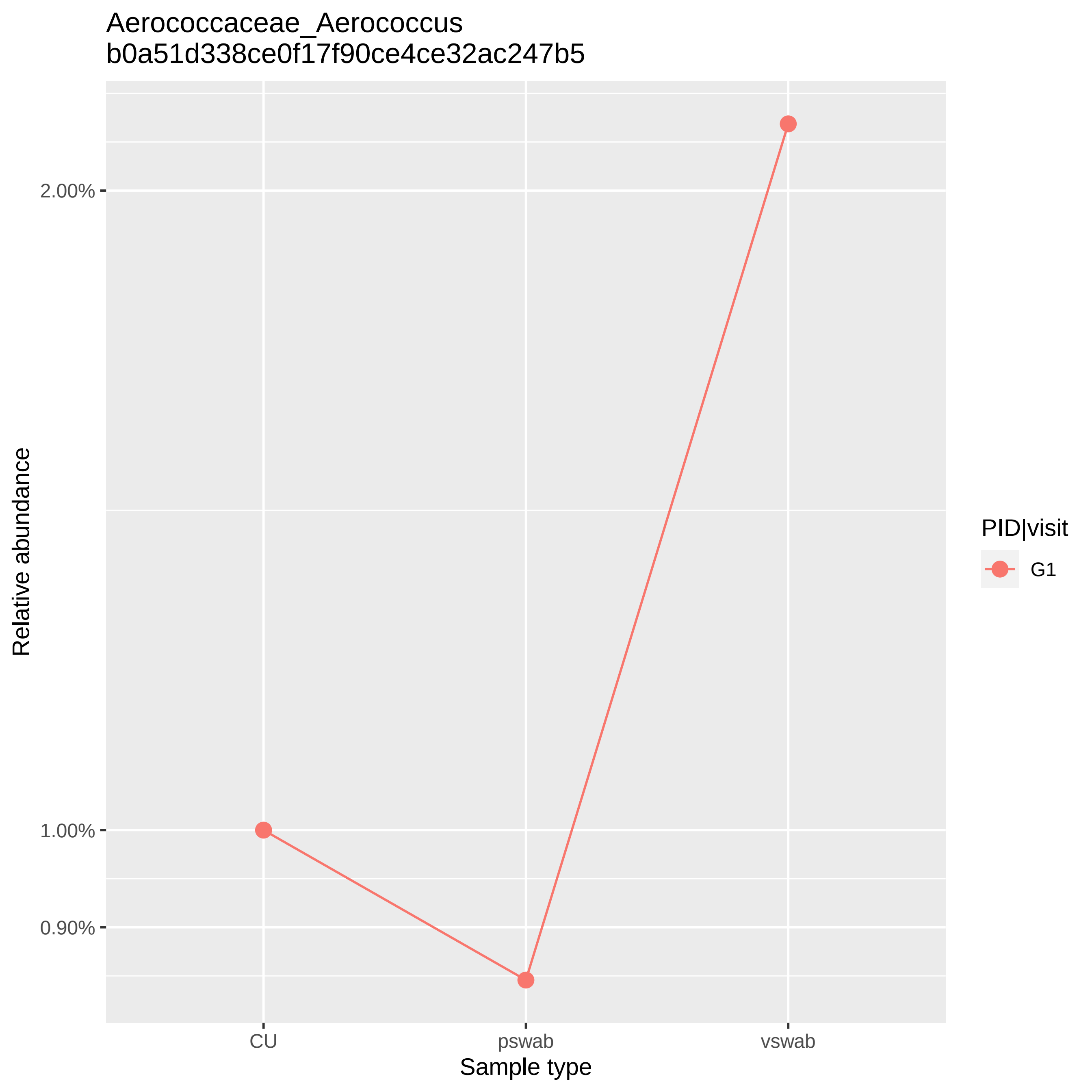
Aerococcaceae_Aerococcus
b0a51d338ce0f17f90ce4ce32ac247b5
2.00%
1.00%
0.90%
Relative abundance
CU
pswab
vswab
Sample type
PID|visit
G1

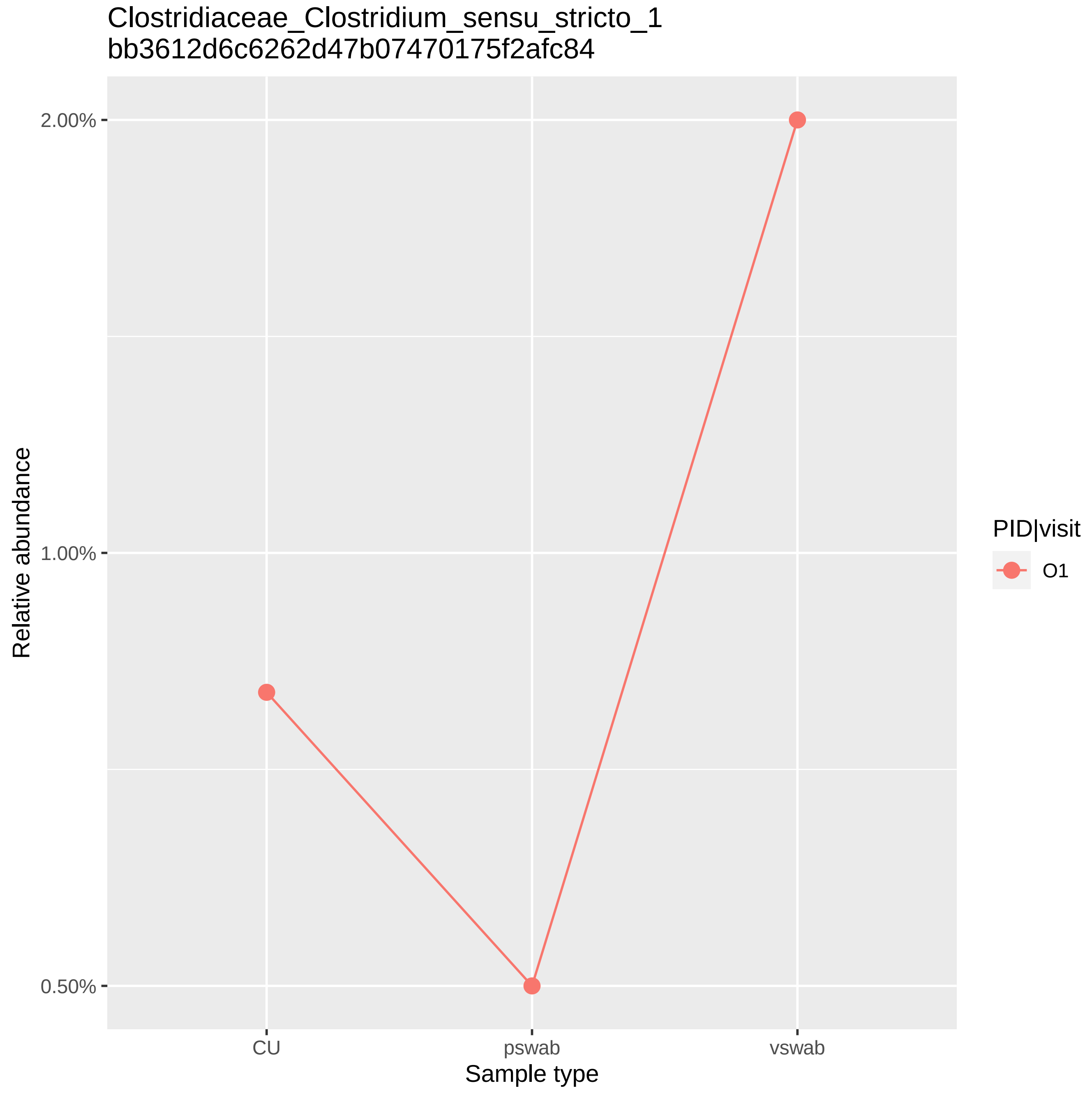
Clostridiaceae_Clostridium_sensu_stricto_1
bb3612d6c6262d47b07470175f2afc84
Relative abundance
2.00%
1.00%
0.50%
CU
pswab
vswab
Sample type
PID|visit
O1

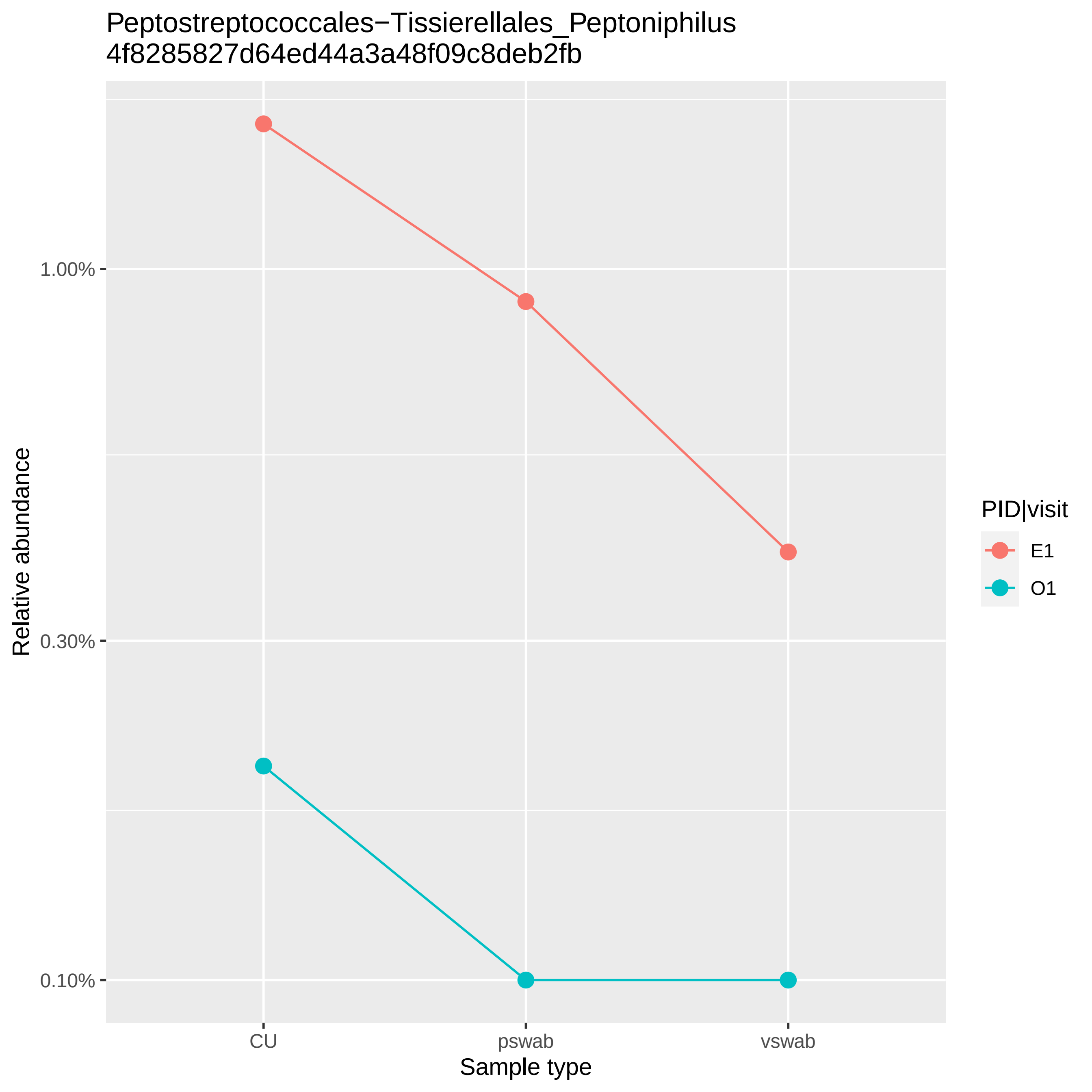

Peptostreptococcales−Tissierellales_Peptoniphilus
4f8285827d64ed44a3a48f09c8deb2fb
Relative abundance
1.00%
0.30%
0.10%
CU
pswab
vswab
Sample type
PID|visit
E1
O1

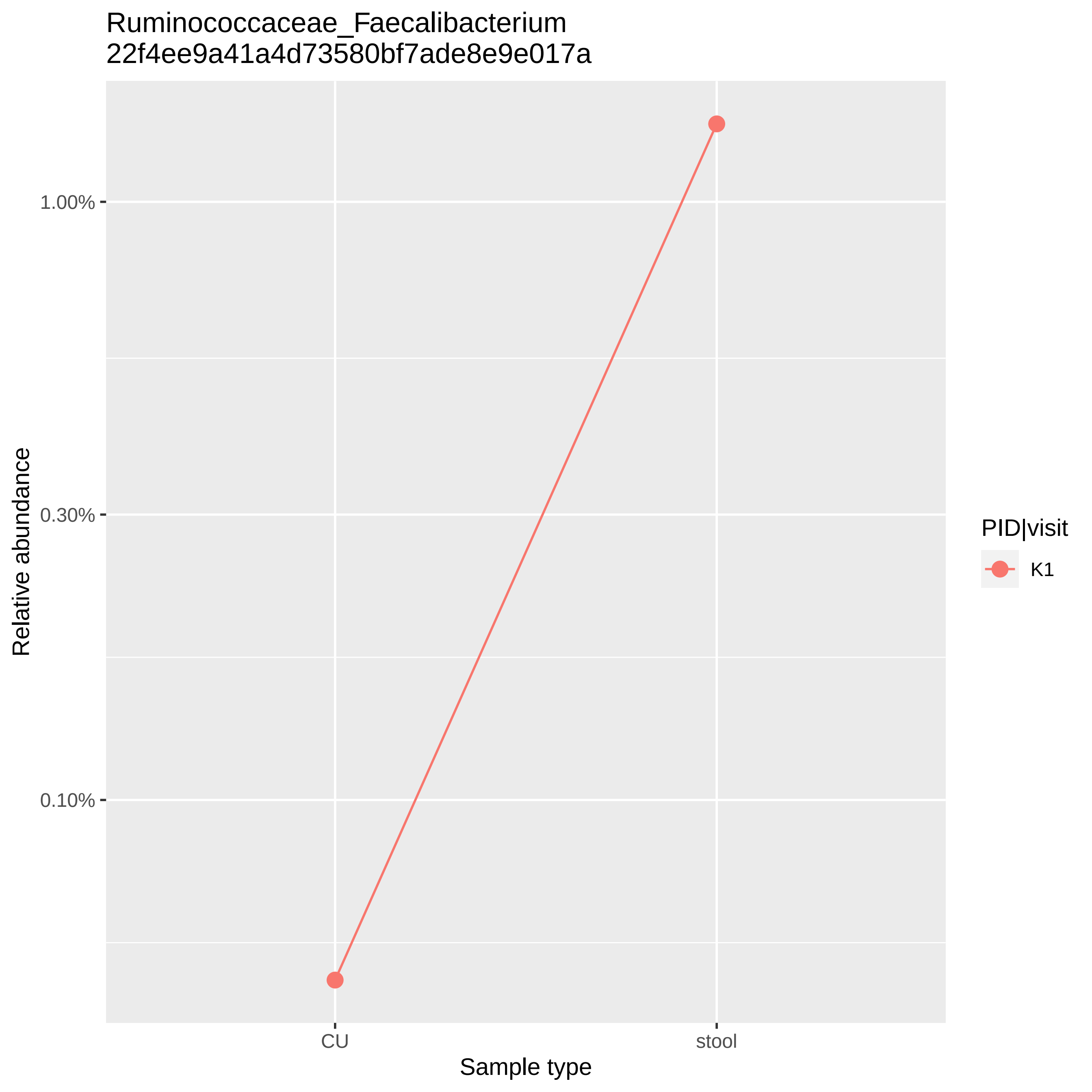
Ruminococcaceae_Faecalibacterium
22f4ee9a41a4d73580bf7ade8e9e017a
Relative abundance
1.00%
0.30%
0.10%
CU
stool
Sample type
PID|visit
K1

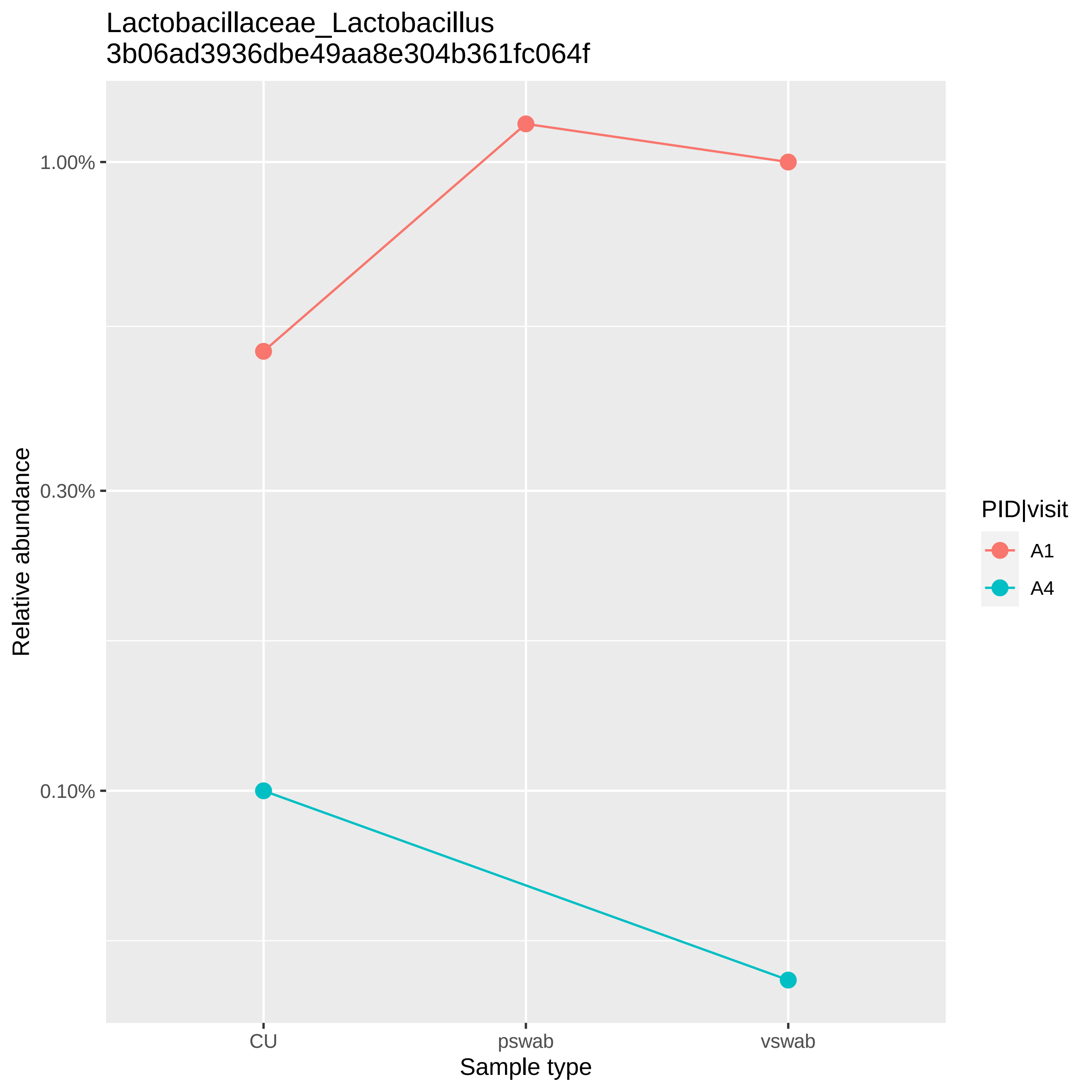

Lactobacillaceae_Lactobacillus
3b06ad3936dbe49aa8e304b361fc064f
1.00%
0.30%
0.10%
Relative abundance
CU
pswab
vswab
Sample type
PID|visit
A1
A4

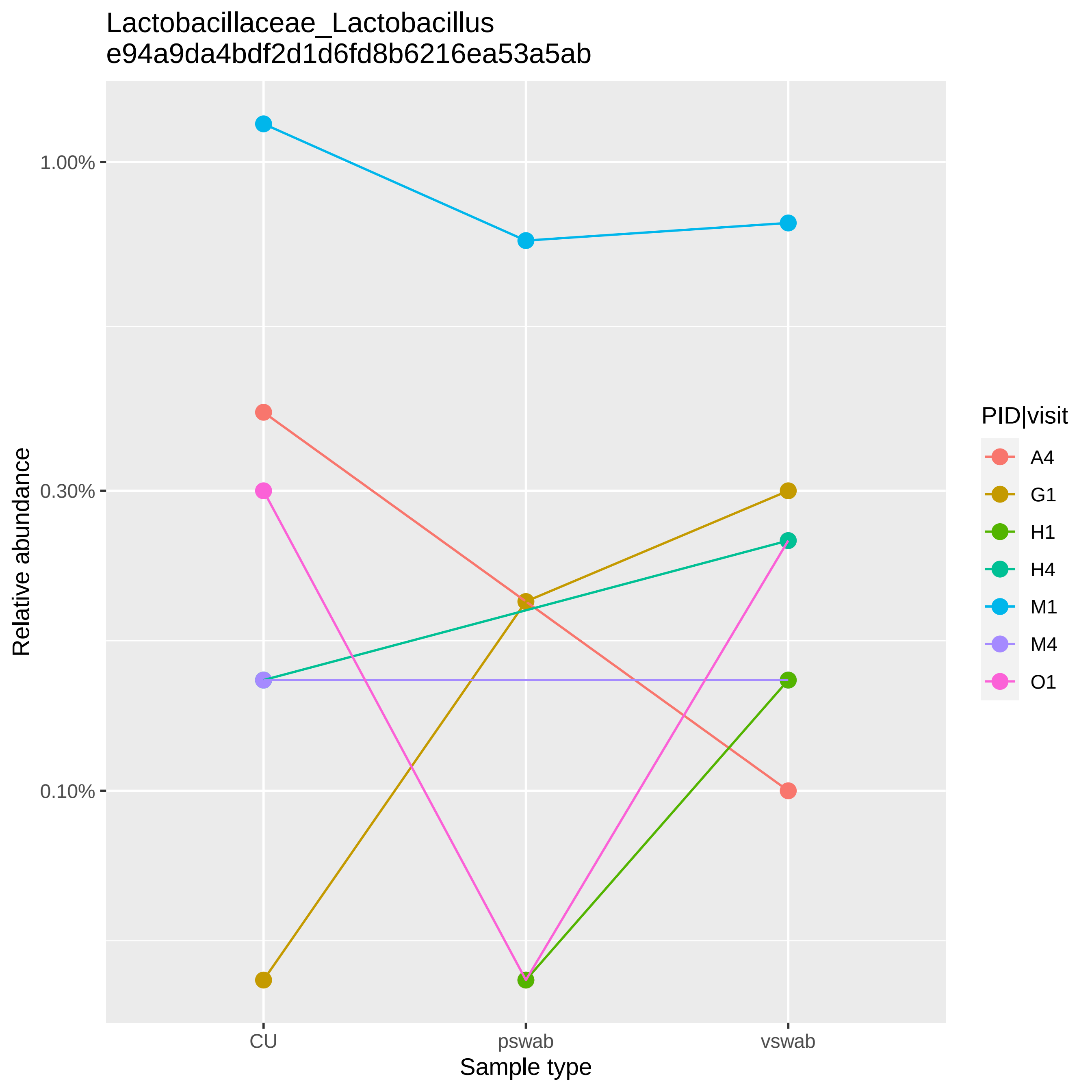
Lactobacillaceae_Lactobacillus
e94a9da4bdf2d1d6fd8b6216ea53a5ab
Relative abundance
1.00%
0.30%
0.10%
CU
pswab
vswab
Sample type
PID|visit
A4
G1
H1
H4
M1
M4
O1

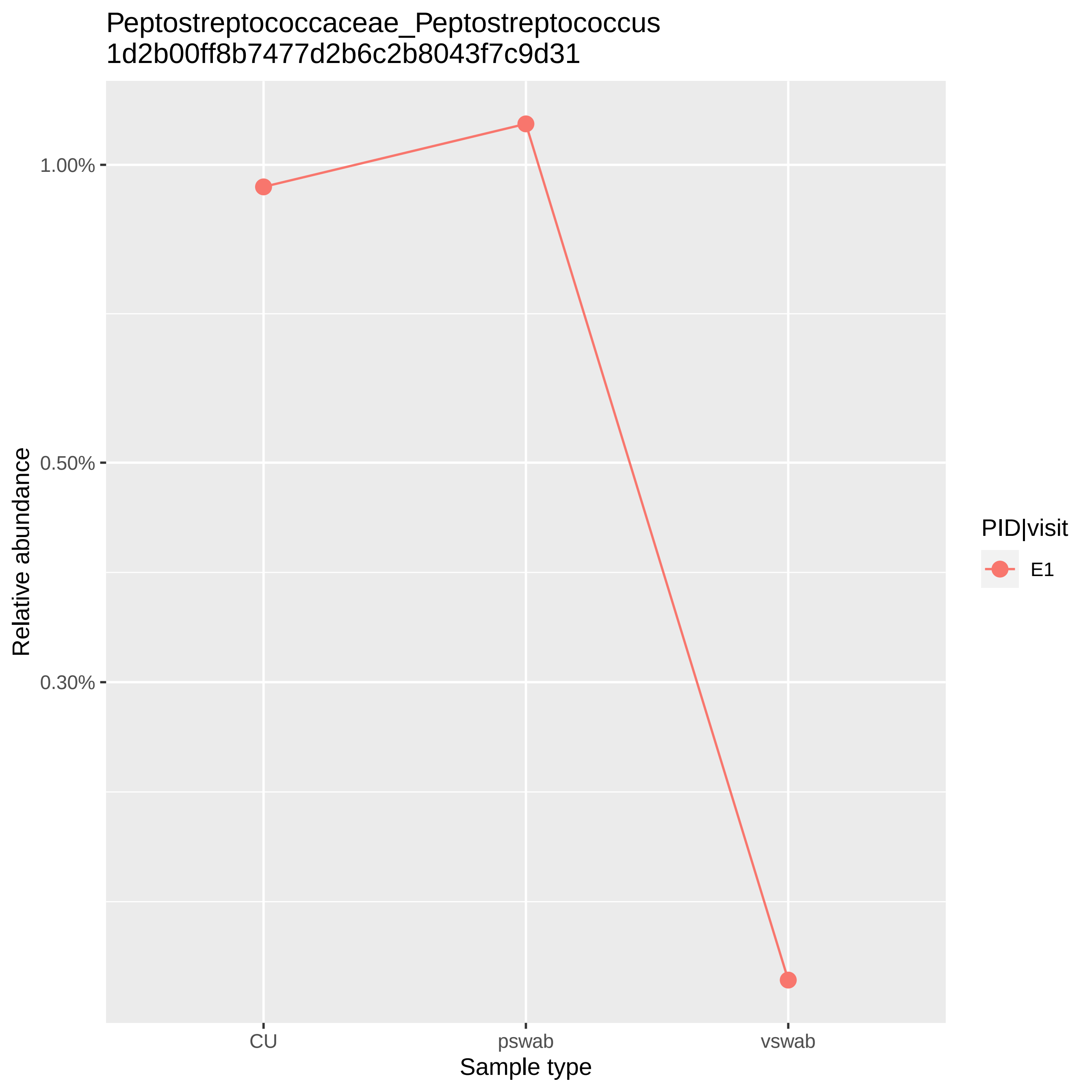

Peptostreptococcaceae_Peptostreptococcus
1d2b00ff8b7477d2b6c2b8043f7c9d31
1.00%
0.50%
0.30%
Relative abundance
CU
pswab
vswab
Sample type
PID|visit
E1

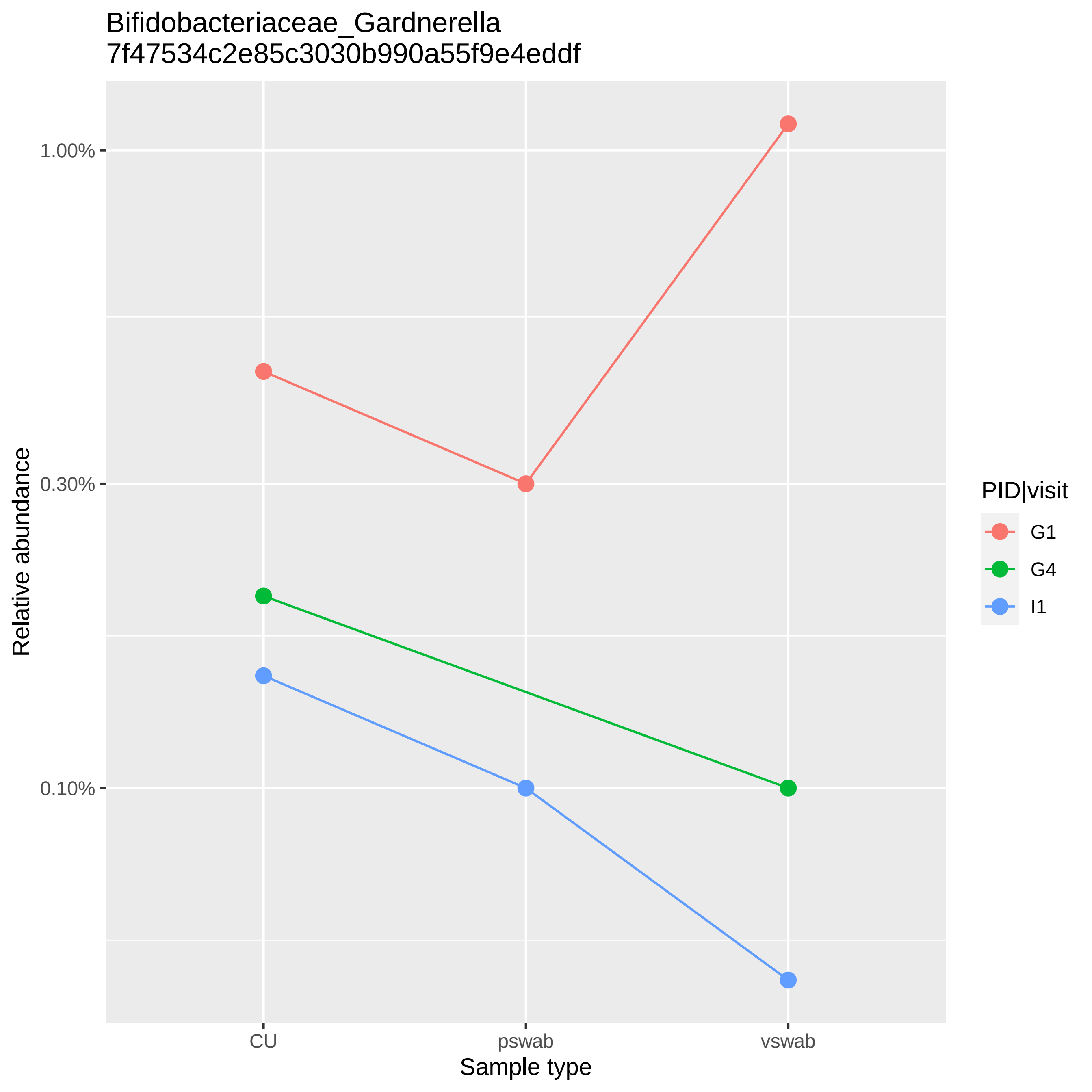
Bifidobacteriaceae_Gardnerella
7f47534c2e85c3030b990a55f9e4eddf
Relative abundance
1.00%
0.30%
0.10%
CU
pswab
vswab
Sample type
PID|visit
G1
G4
I1

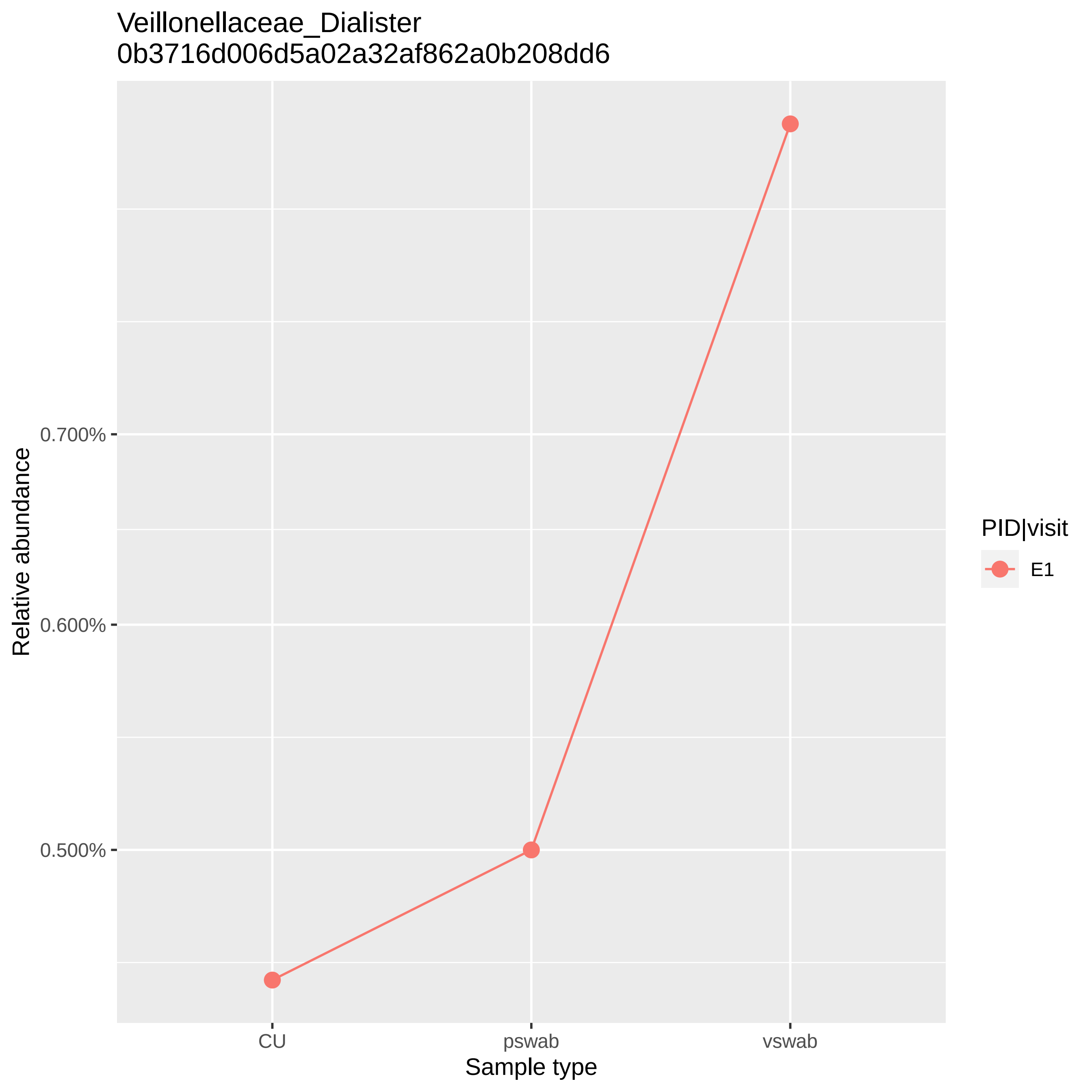

Veillonellaceae_Dialister
0b3716d006d5a02a32af862a0b208dd6
Relative abundance
0.700%
0.600%
0.500%
CU
pswab
vswab
Sample type
PID|visit
E1

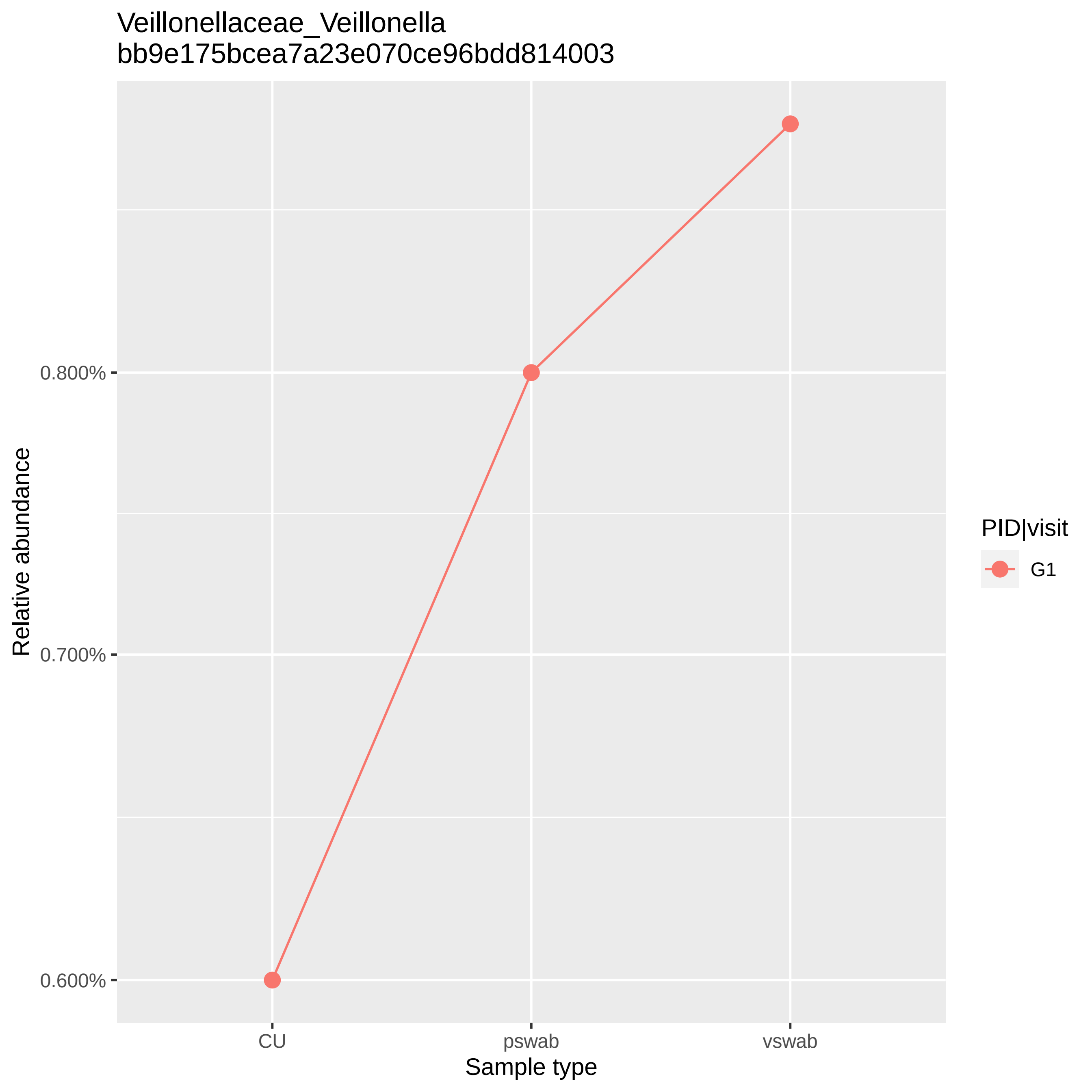

Veillonellaceae_Veillonella
bb9e175bcea7a23e070ce96bdd814003
0.800%
0.700%
0.600%
Relative abundance
CU
pswab
vswab
Sample type
PID|visit
G1

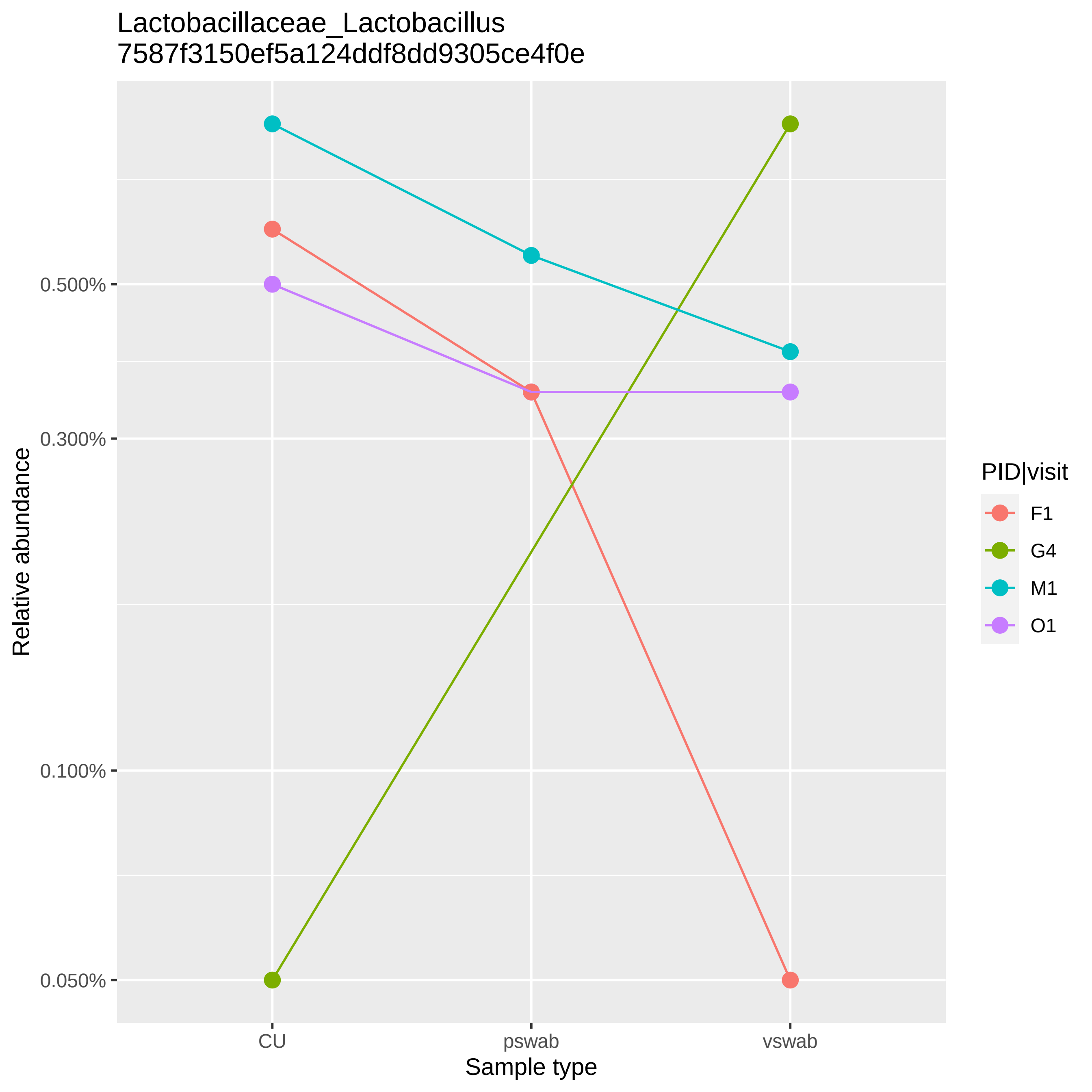

Lactobacillaceae_Lactobacillus
7587f3150ef5a124ddf8dd9305ce4f0e
0.500%
0.300%
0.100%
0.050%
Relative abundance
CU
pswab
vswab
Sample type
PID|visit
F1
G4
M1
O1

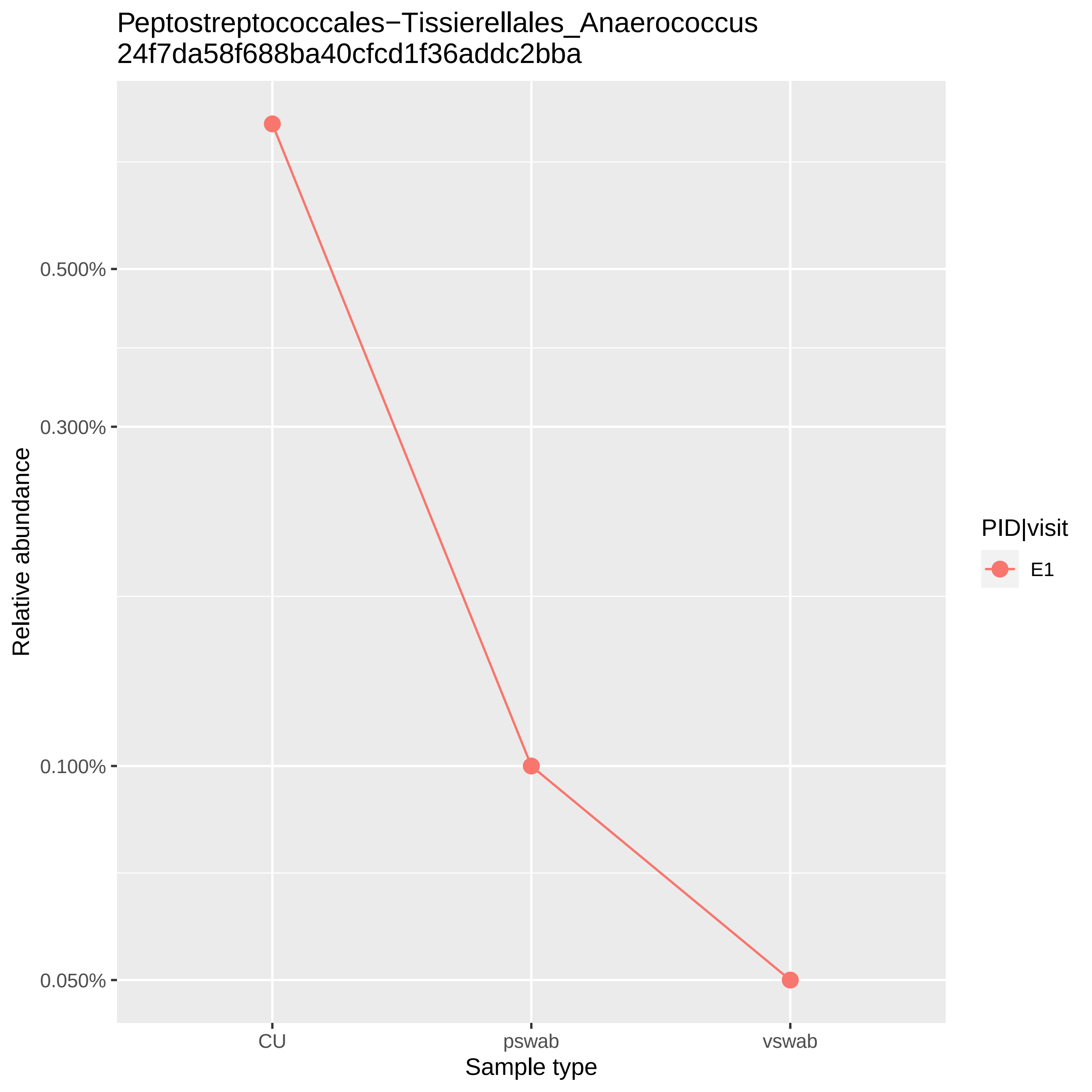

Peptostreptococcales−Tissierellales_Anaerococcus
24f7da58f688ba40cfcd1f36addc2bba
0.500%
0.300%
0.100%
0.050%
Relative abundance
CU
pswab
vswab
Sample type
PID|visit
E1

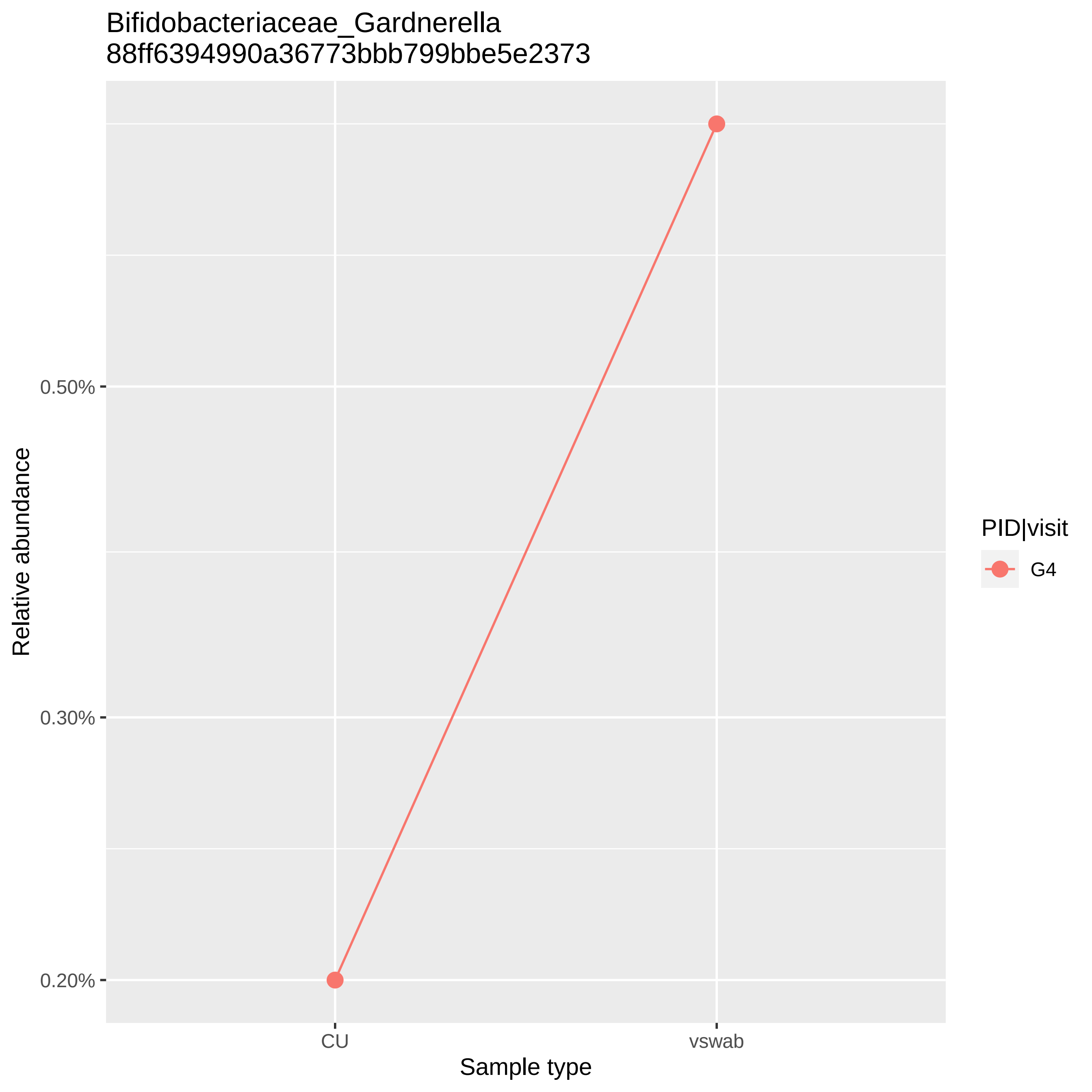

Bifidobacteriaceae_Gardnerella
88ff6394990a36773bbb799bbe5e2373
0.50%
0.30%
0.20%
Relative abundance
CU
vswab
Sample type
PID|visit
G4

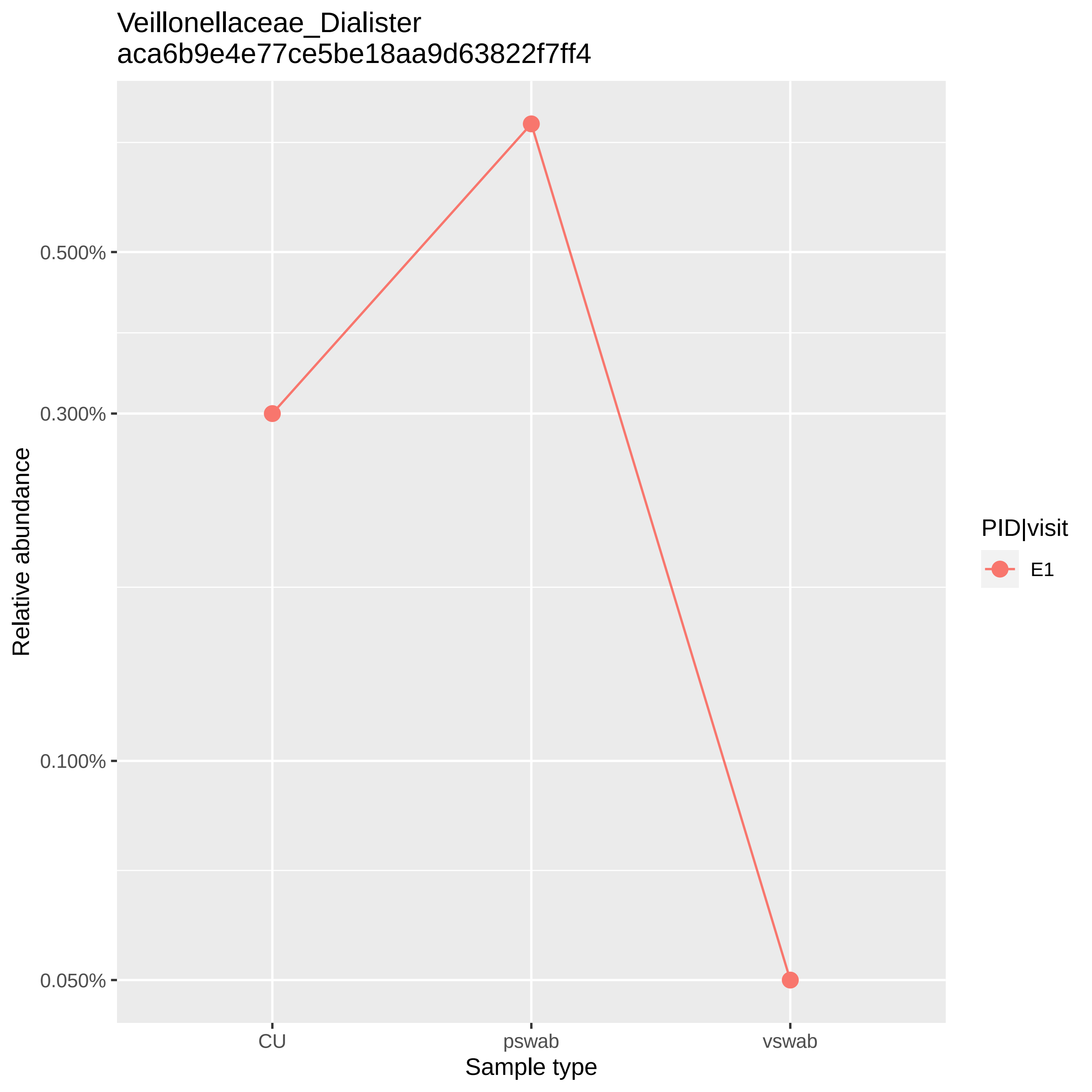

Veillonellaceae_Dialister
aca6b9e4e77ce5be18aa9d63822f7ff4
0.500%
0.300%
0.100%
0.050%
Relative abundance
CU
pswab
vswab
Sample type
PID|visit
E1

Eggerthellaceae_DNF00809
5ca5046fc62298fc019349227d53ac5a

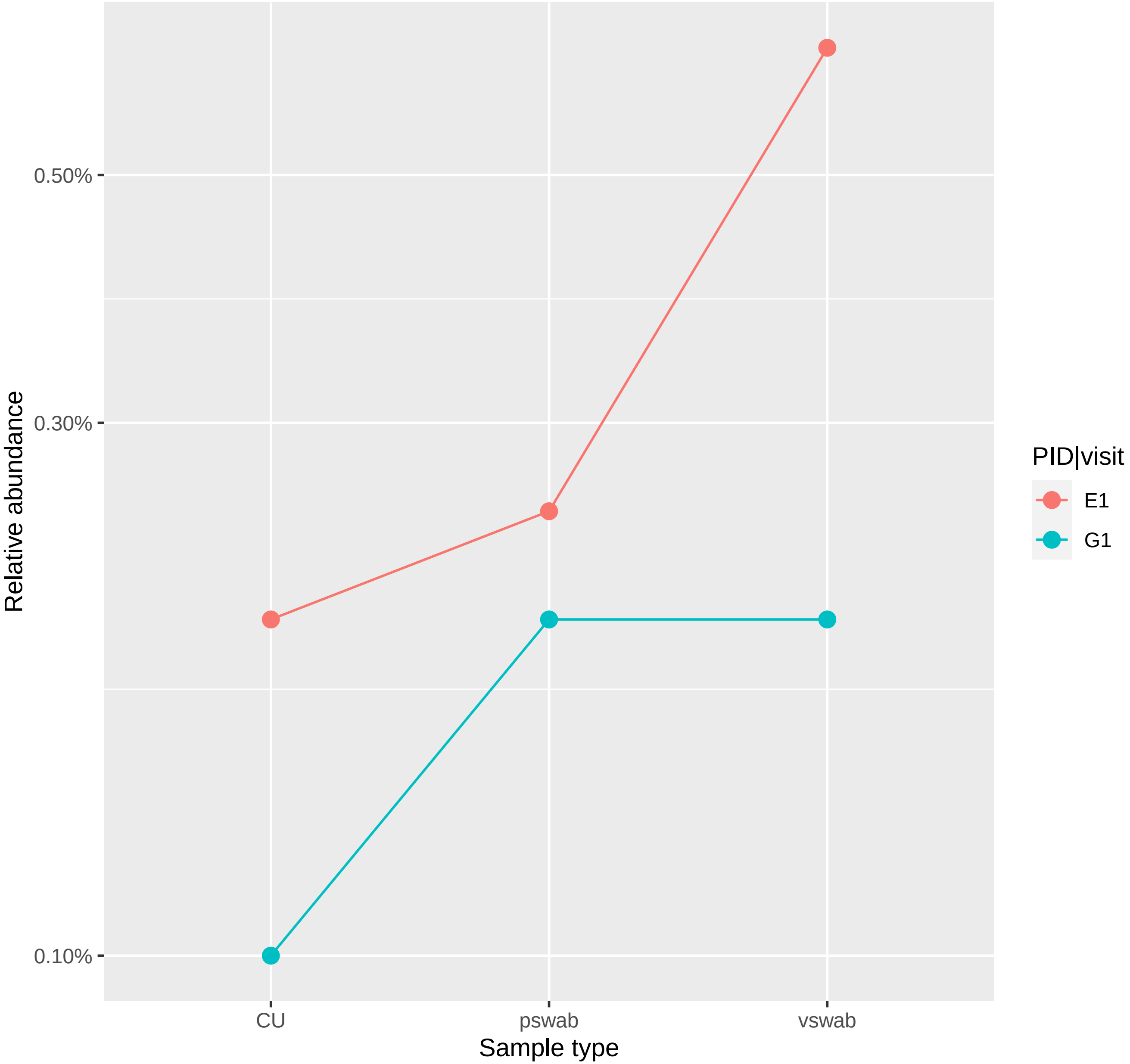

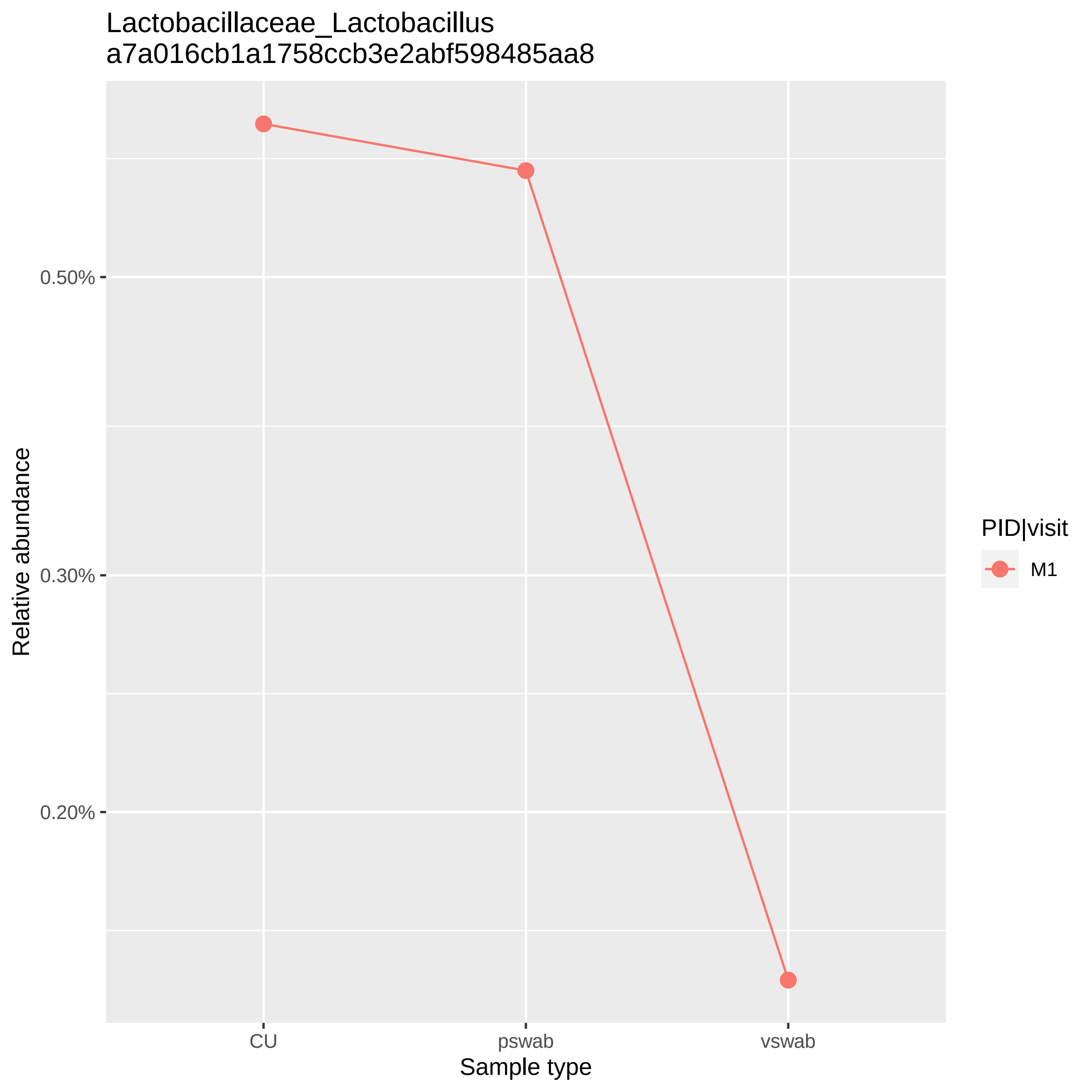
Lactobacillaceae_Lactobacillus
a7a016cb1a1758ccb3e2abf598485aa8
0.50%
0.30%
0.20%
Relative abundance
CU
pswab
vswab
Sample type
PID|visit
M1

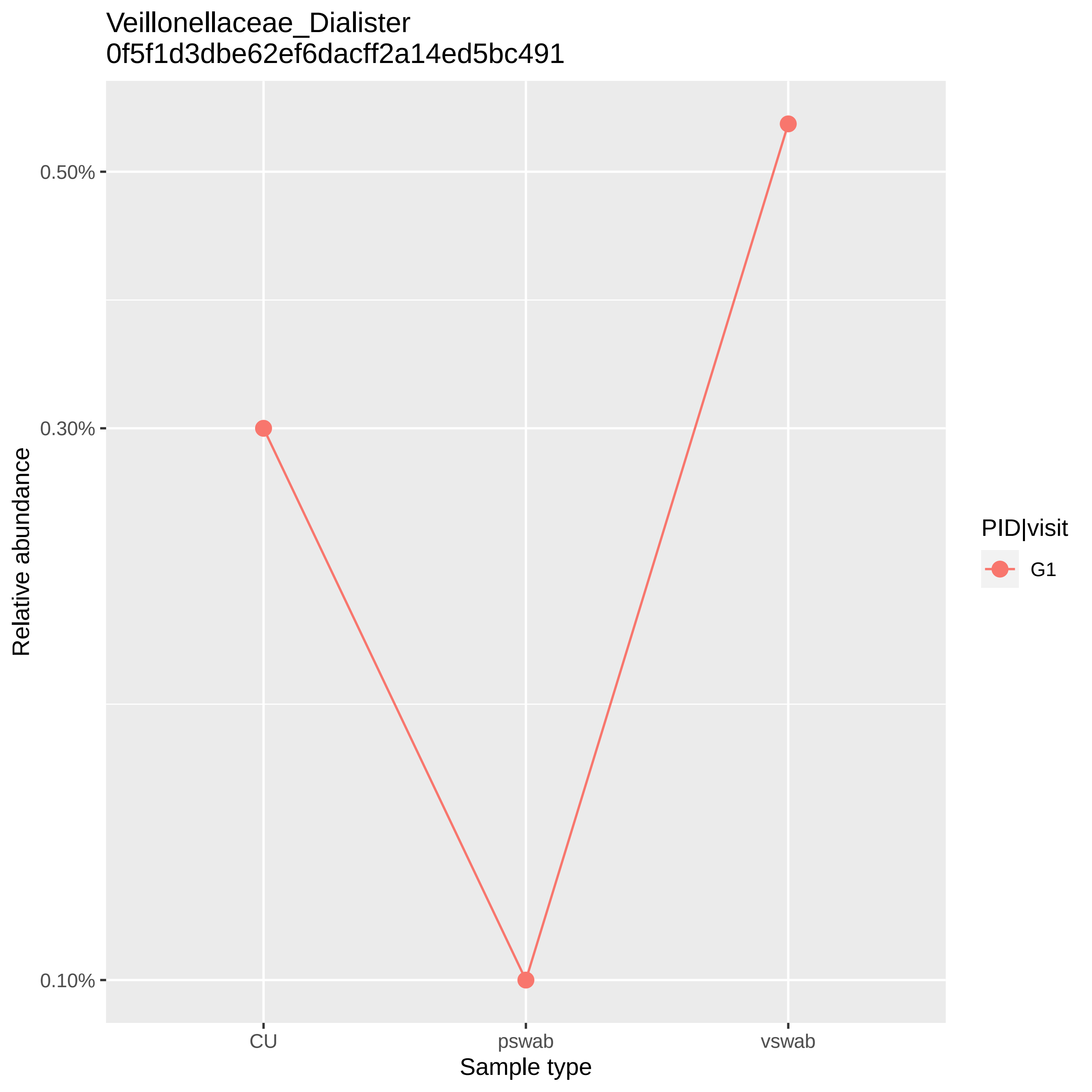
Veillonellaceae_Dialister
0f5f1d3dbe62ef6dacff2a14ed5bc491
0.50%
0.30%
0.10%
Relative abundance
CU
pswab
vswab
Sample type
PID|visit
G1

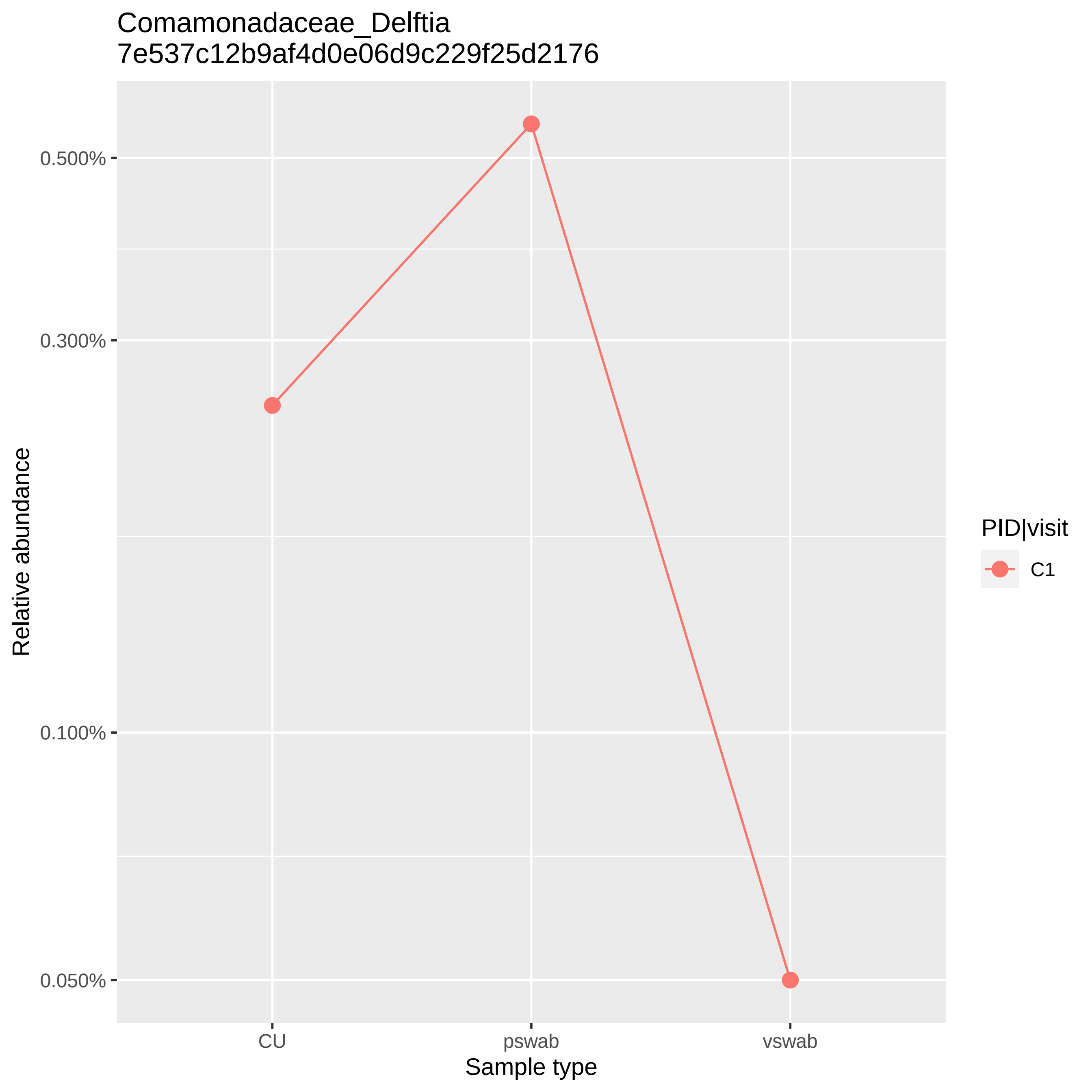
Comamonadaceae_Delftia
7e537c12b9af4d0e06d9c229f25d2176
0.500%
0.300%
0.100%
0.050%
Relative abundance
CU
pswab
vswab
Sample type
PID|visit
C1

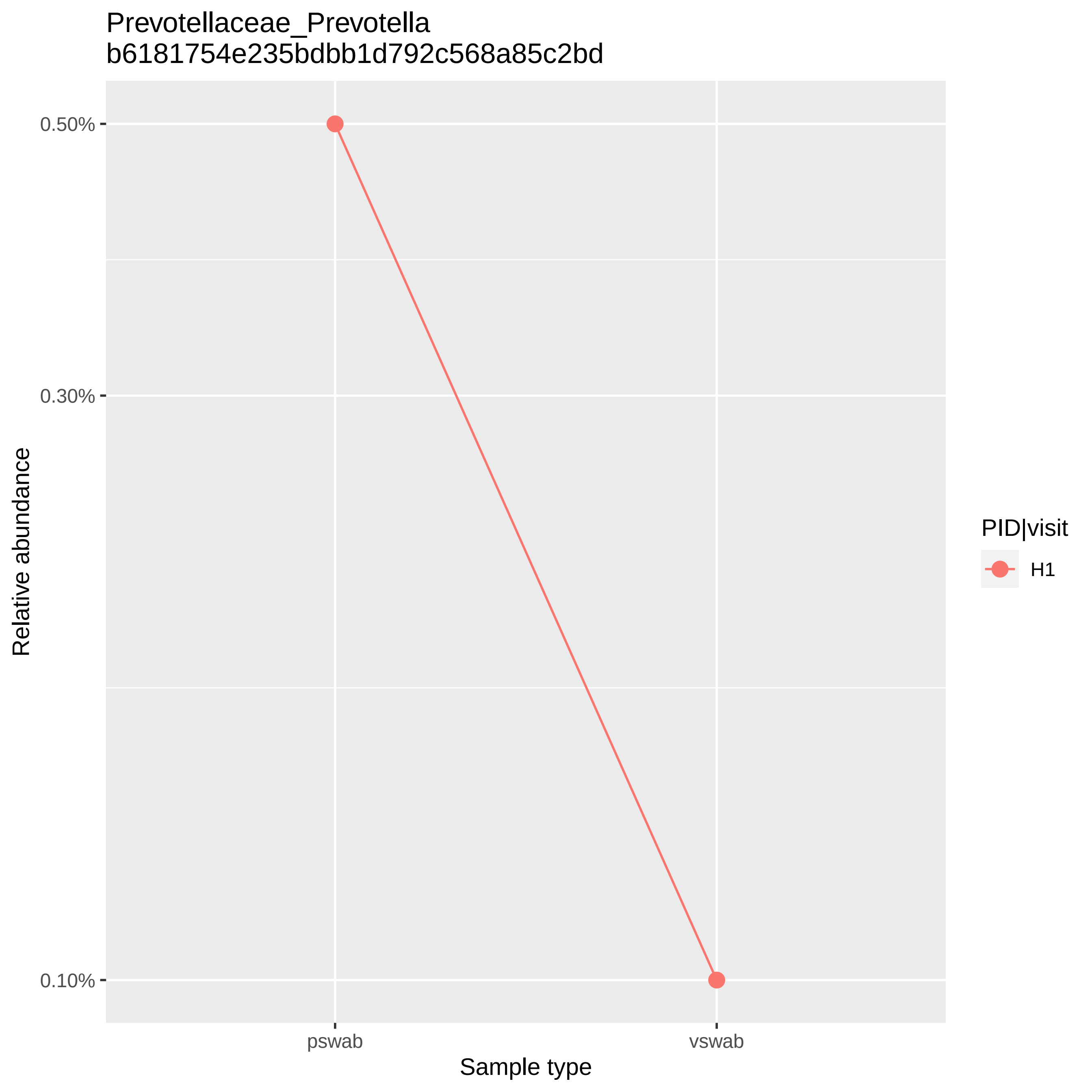
Prevotellaceae_Prevotella
b6181754e235bdbb1d792c568a85c2bd
0.50%
0.30%
0.10%
Relative abundance
pswab
vswab
Sample type
PID|visit
H1

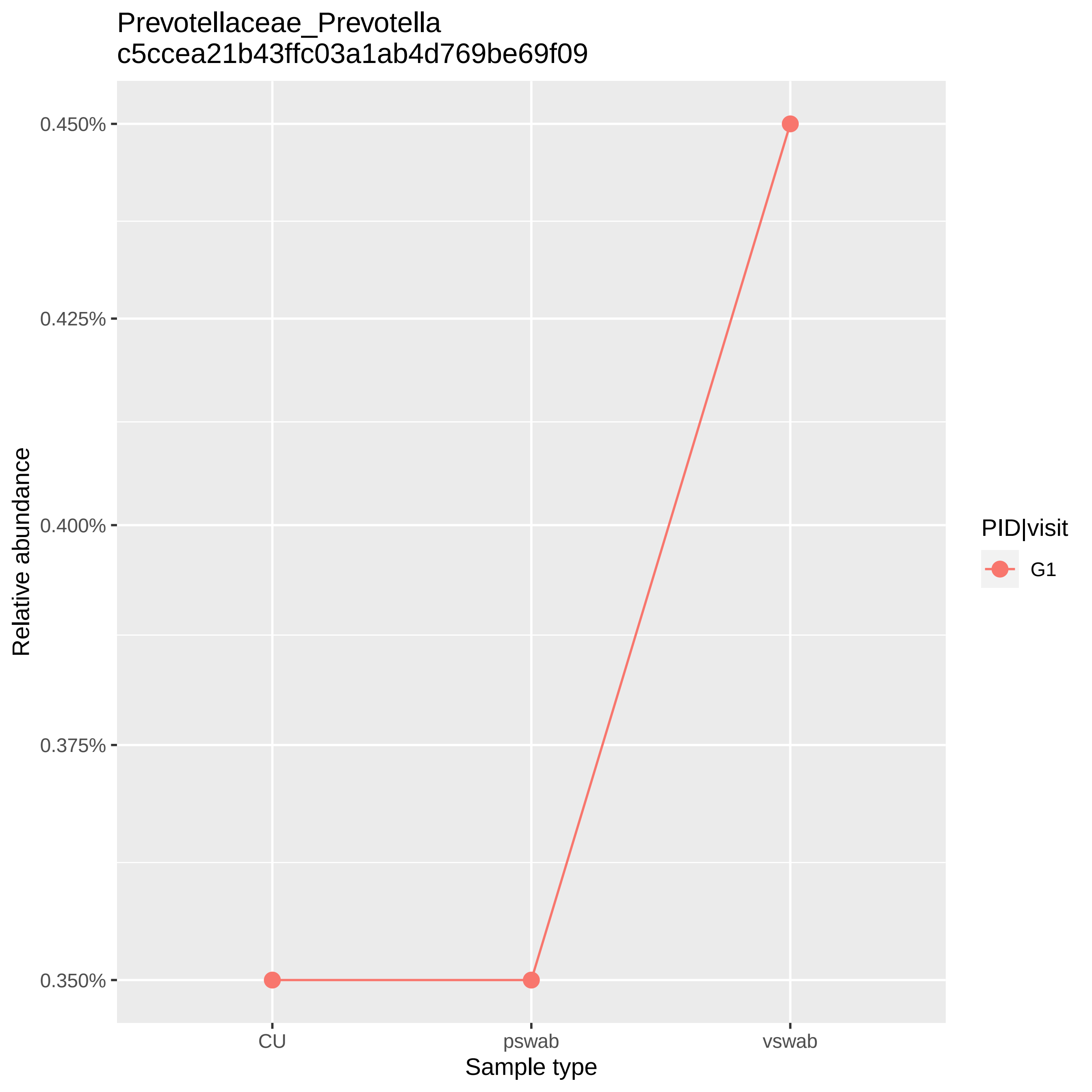
Prevotellaceae_Prevotella
c5ccea21b43ffc03a1ab4d769be69f09
Relative abundance
0.450%
0.425%
0.400%
0.375%
0.350%
CU
pswab
vswab
Sample type
PID|visit
G1

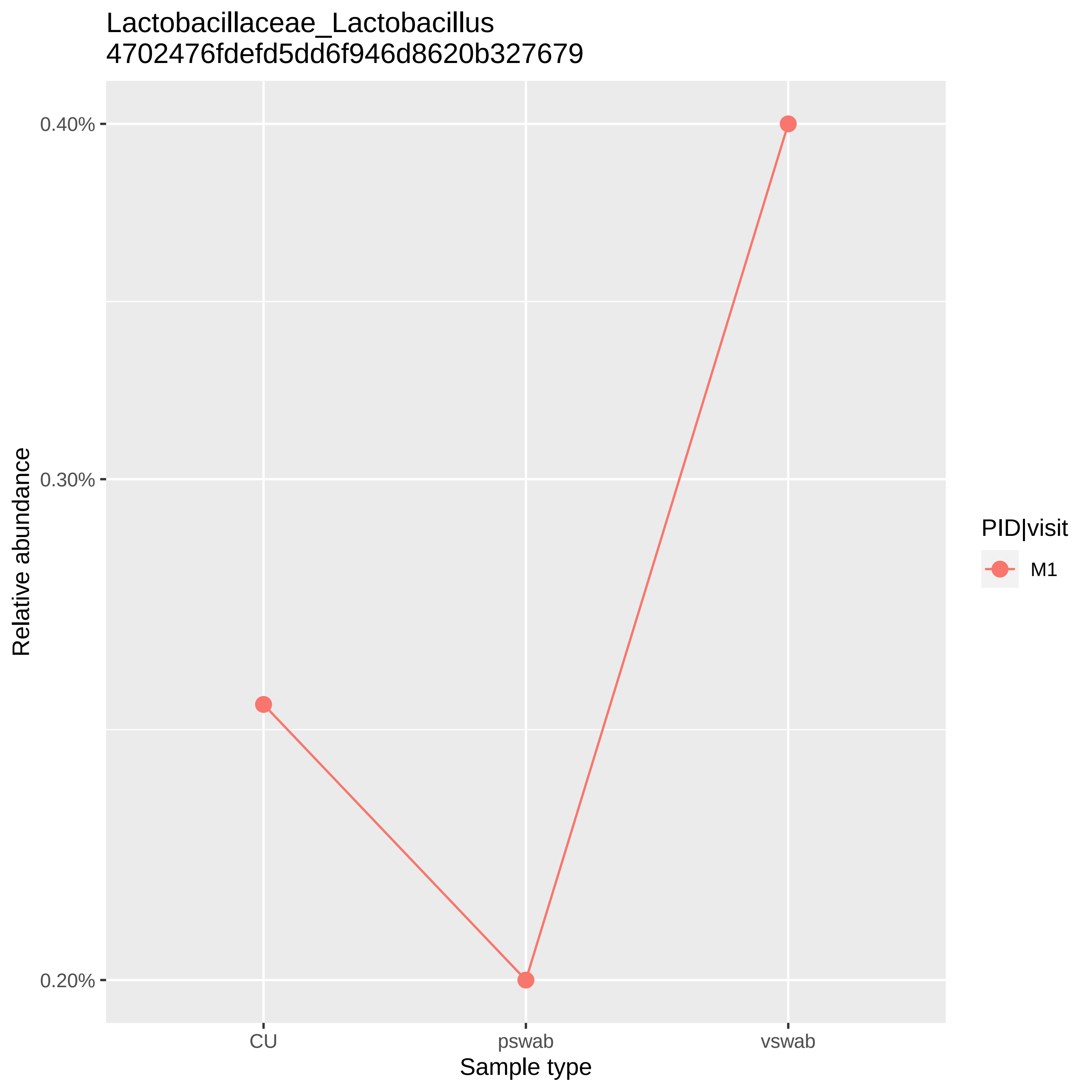
Lactobacillaceae_Lactobacillus
4702476fdefd5dd6f946d8620b327679
0.40%
0.30%
0.20%
Relative abundance
CU
pswab
vswab
Sample type
PID|visit
M1

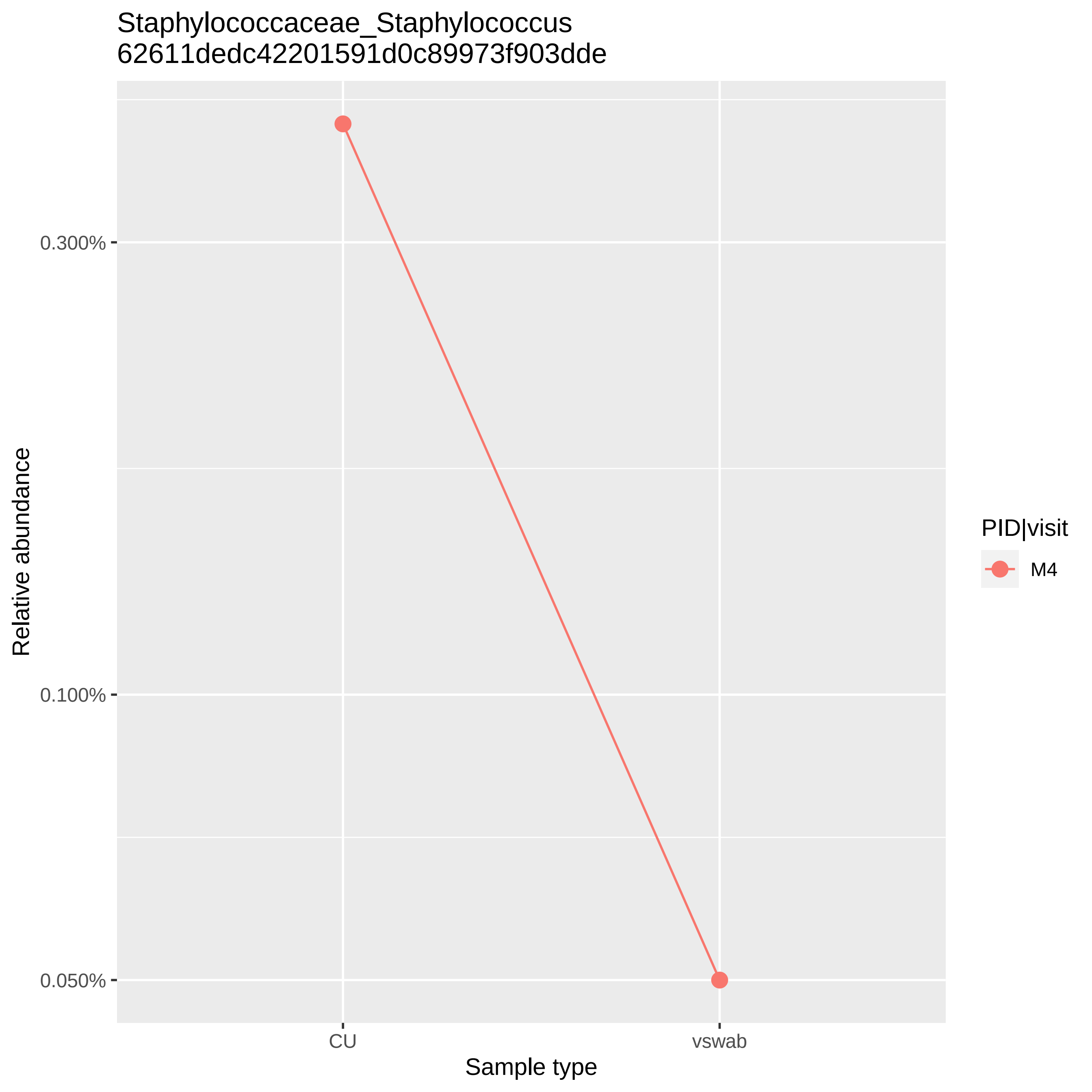
Staphylococcaceae_Staphylococcus
62611dedc42201591d0c89973f903dde
0.300%
0.100%
0.050%
Relative abundance
CU
vswab
Sample type
PID|visit
M4

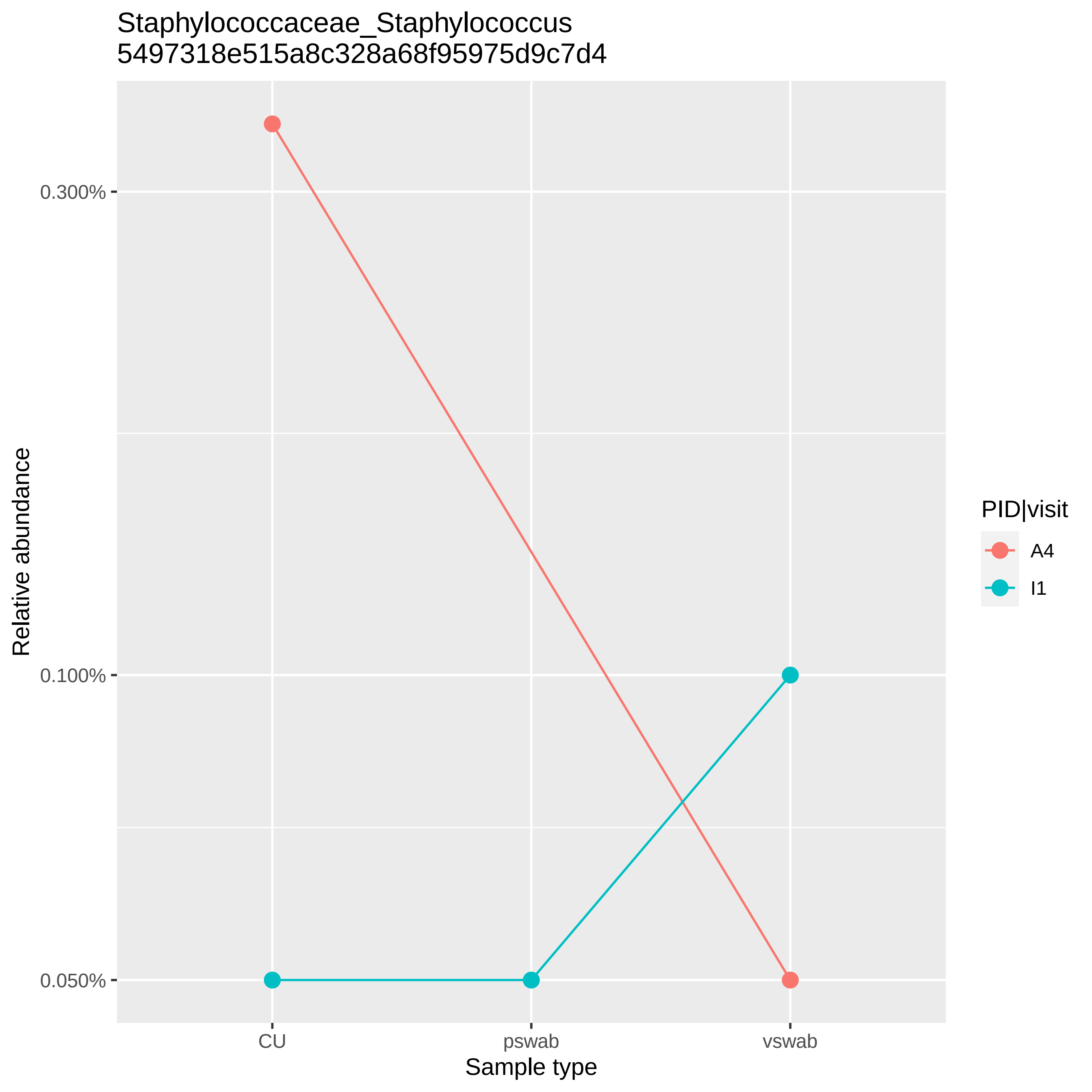
Staphylococcaceae_Staphylococcus
5497318e515a8c328a68f95975d9c7d4
0.300%
0.100%
0.050%
Relative abundance
CU
pswab
vswab
Sample type
PID|visit
A4
I1

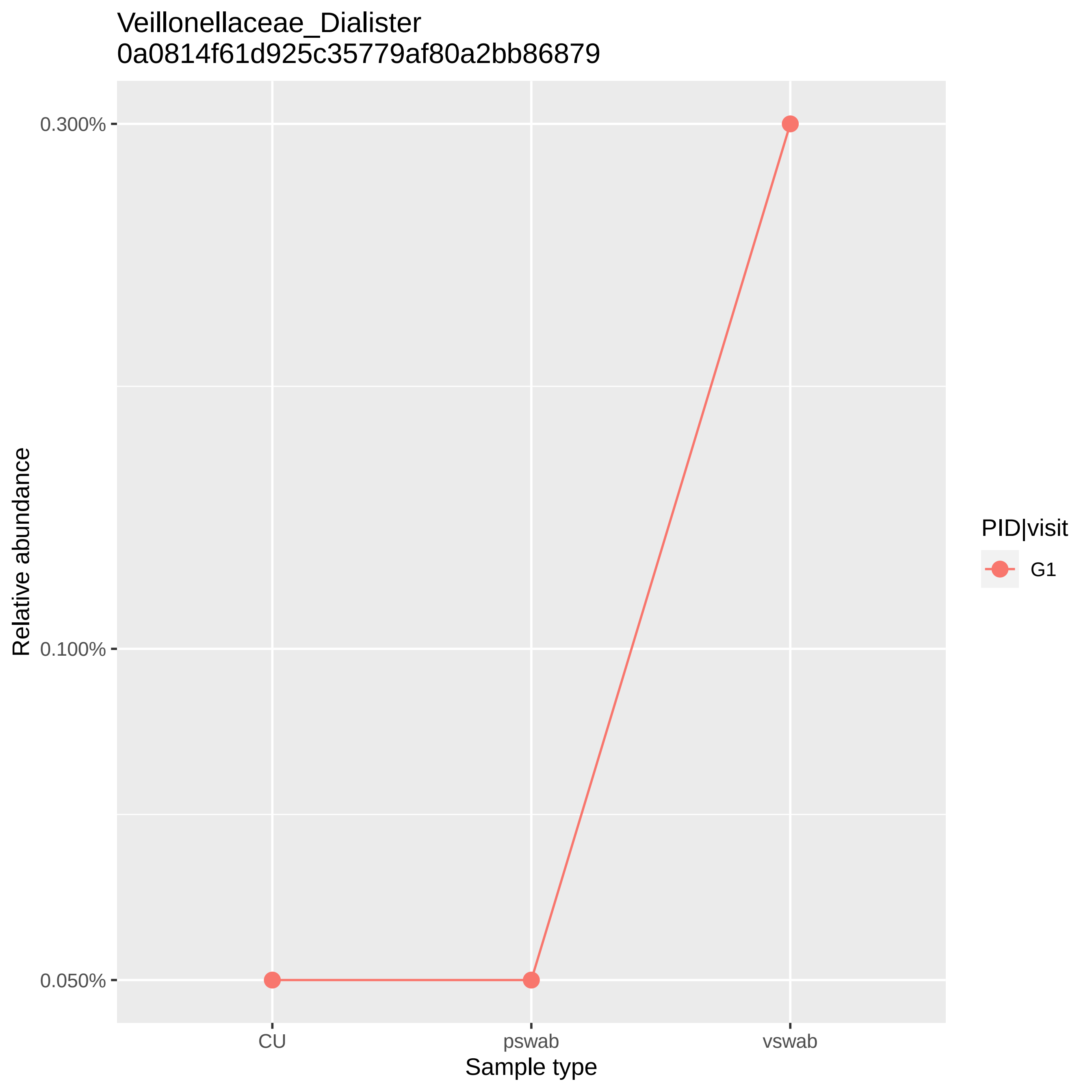

Veillonellaceae_Dialister
0a0814f61d925c35779af80a2bb86879
Relative abundance
0.300%
0.100%
0.050%
CU
pswab
vswab
Sample type
PID|visit
G1

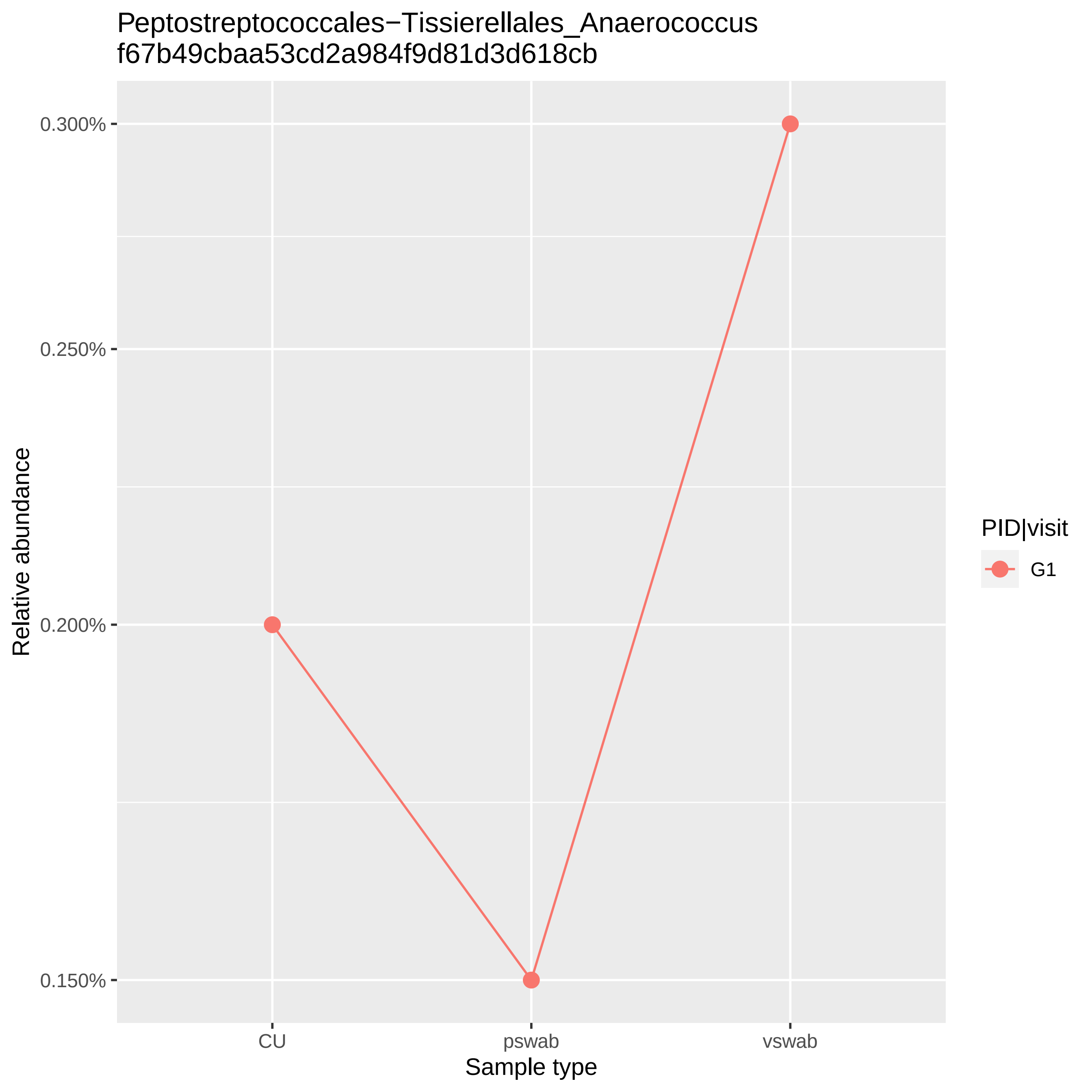
Peptostreptococcales−Tissierellales_Anaerococcus
f67b49cbaa53cd2a984f9d81d3d618cb
0.300%
0.250%
0.200%
0.150%
Relative abundance
CU
pswab
vswab
Sample type
PID|visit
G1

Lactobacillaceae_Lactobacillus
62f5c24b0711ea91ea9eb44fdbe3e550

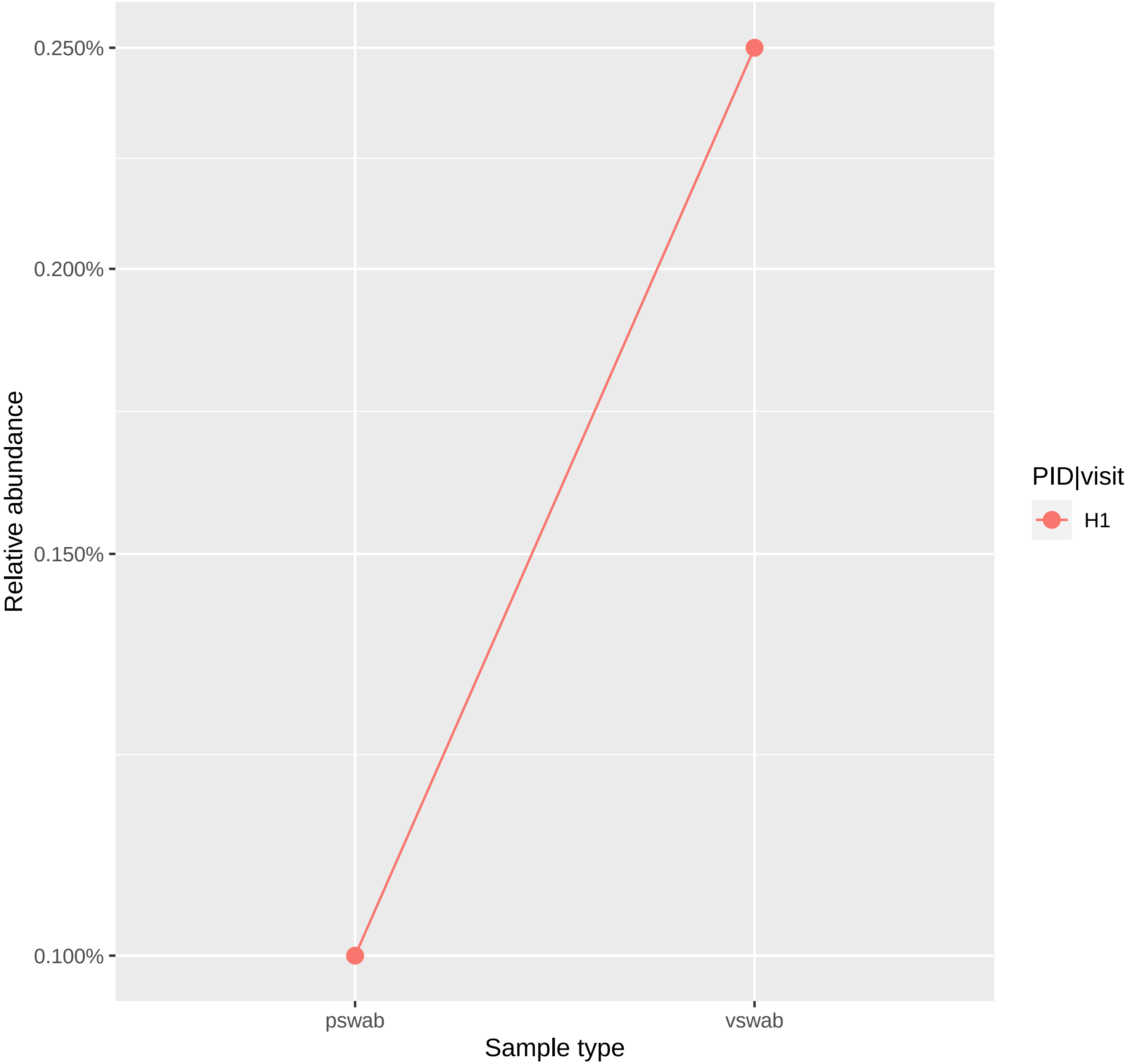

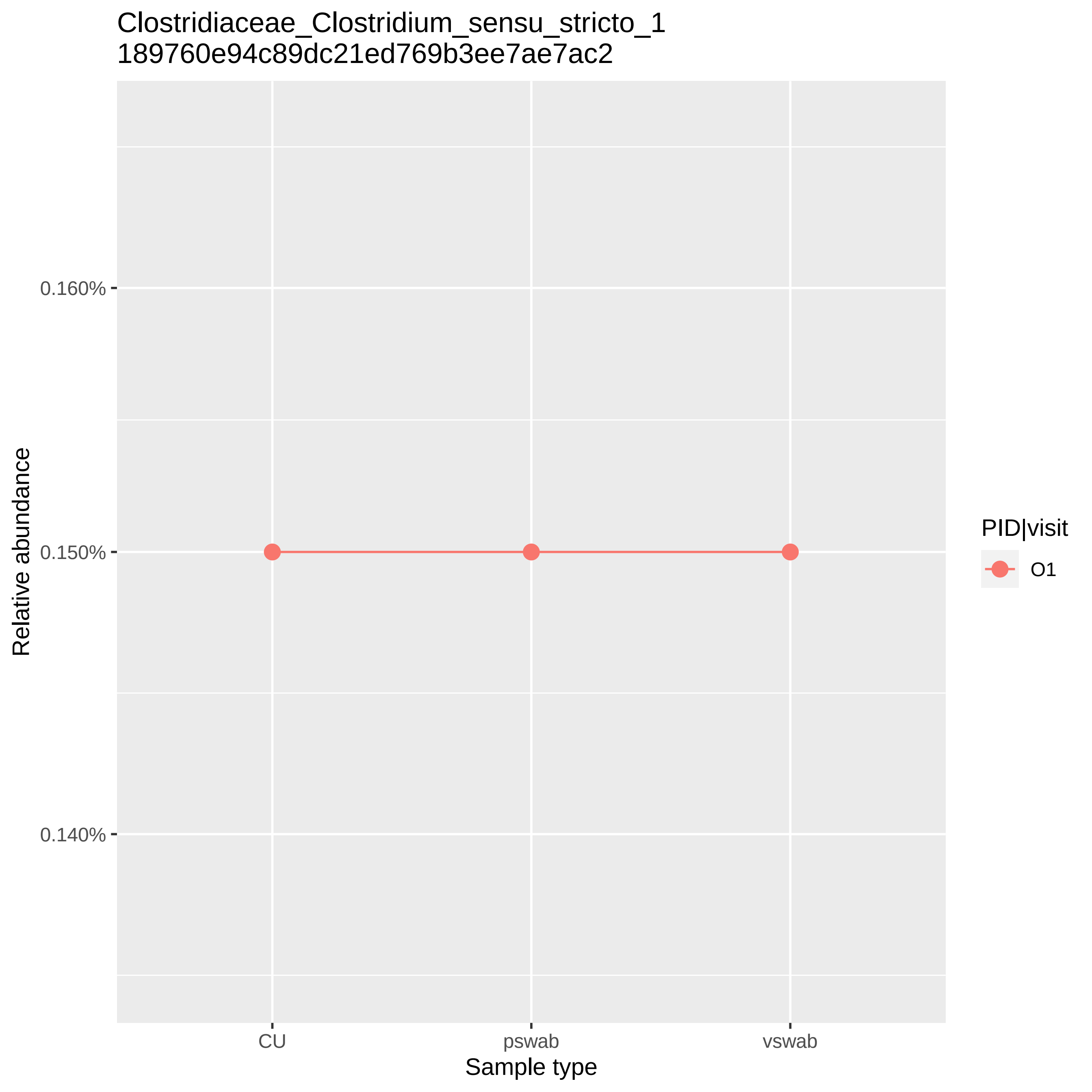
Clostridiaceae_Clostridium_sensu_stricto_1
189760e94c89dc21ed769b3ee7ae7ac2
0.160%
0.150%
0.140%
Relative abundance
CU
pswab
vswab
Sample type
PID|visit
O1

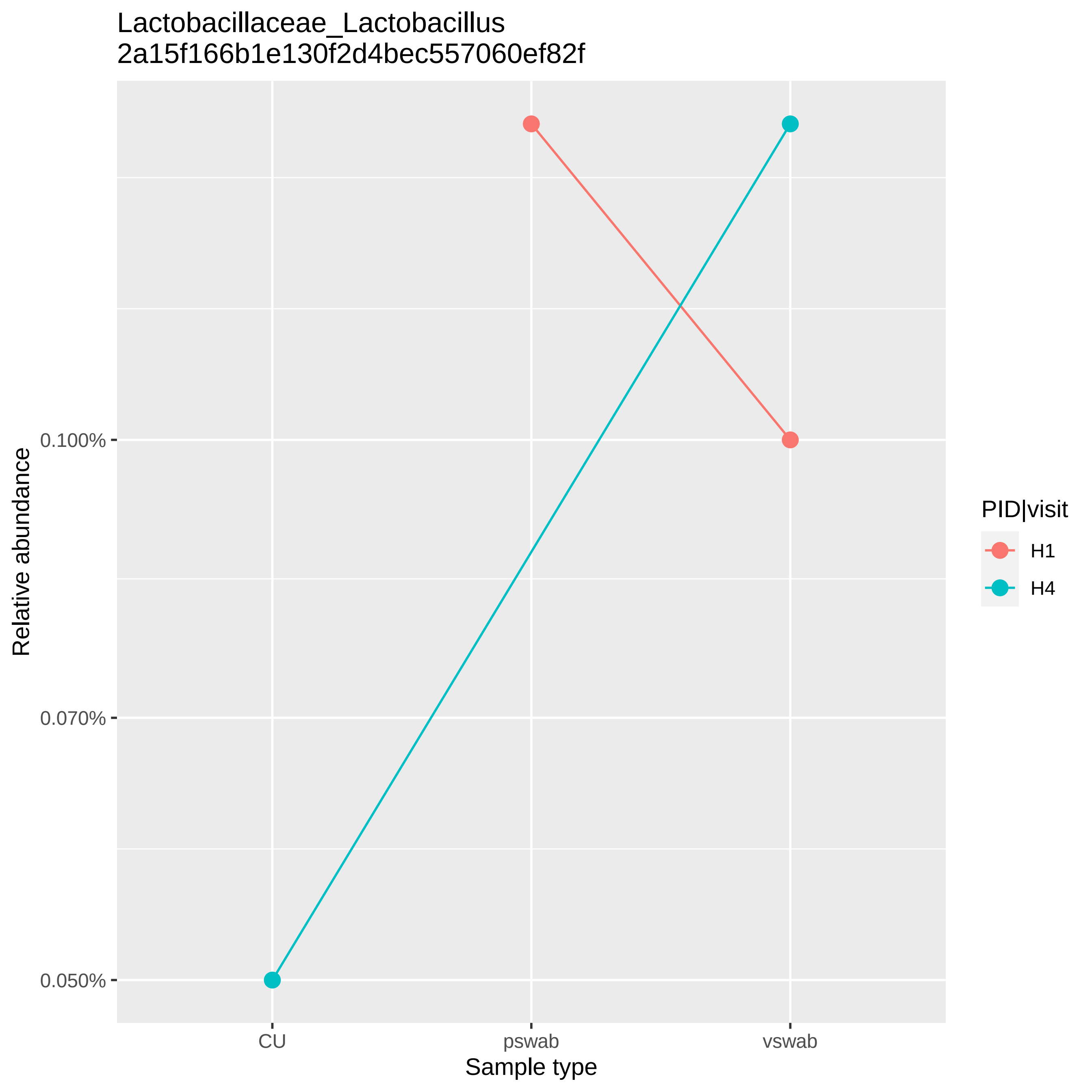

Lactobacillaceae_Lactobacillus
2a15f166b1e130f2d4bec557060ef82f
Relative abundance
0.100%
0.070%
0.050%
CU
pswab
vswab
Sample type
PID|visit
H1
H4

Lactobacillaceae_Lactobacillus
79462ab1bf468975d337f4e03fe8500b

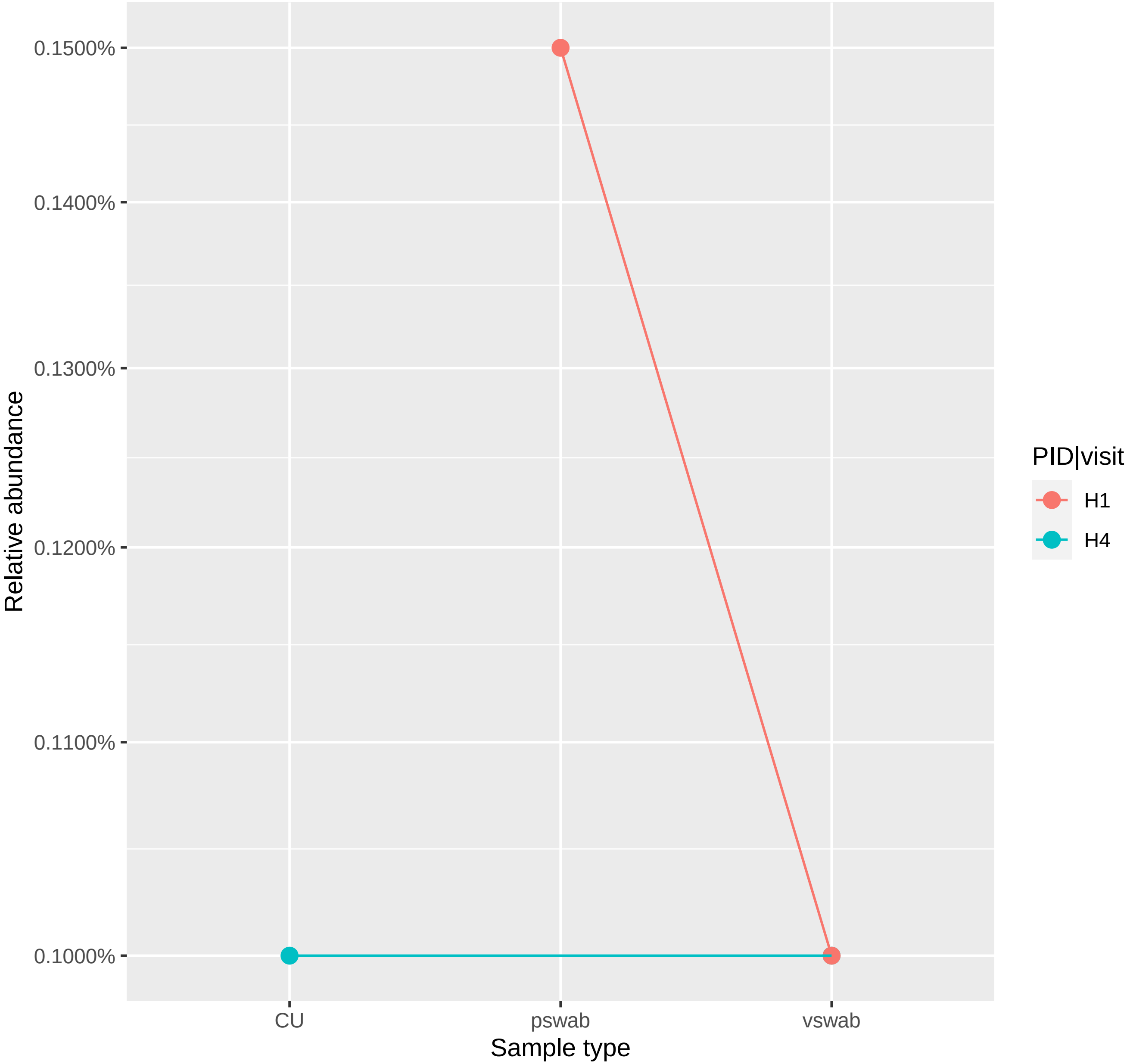

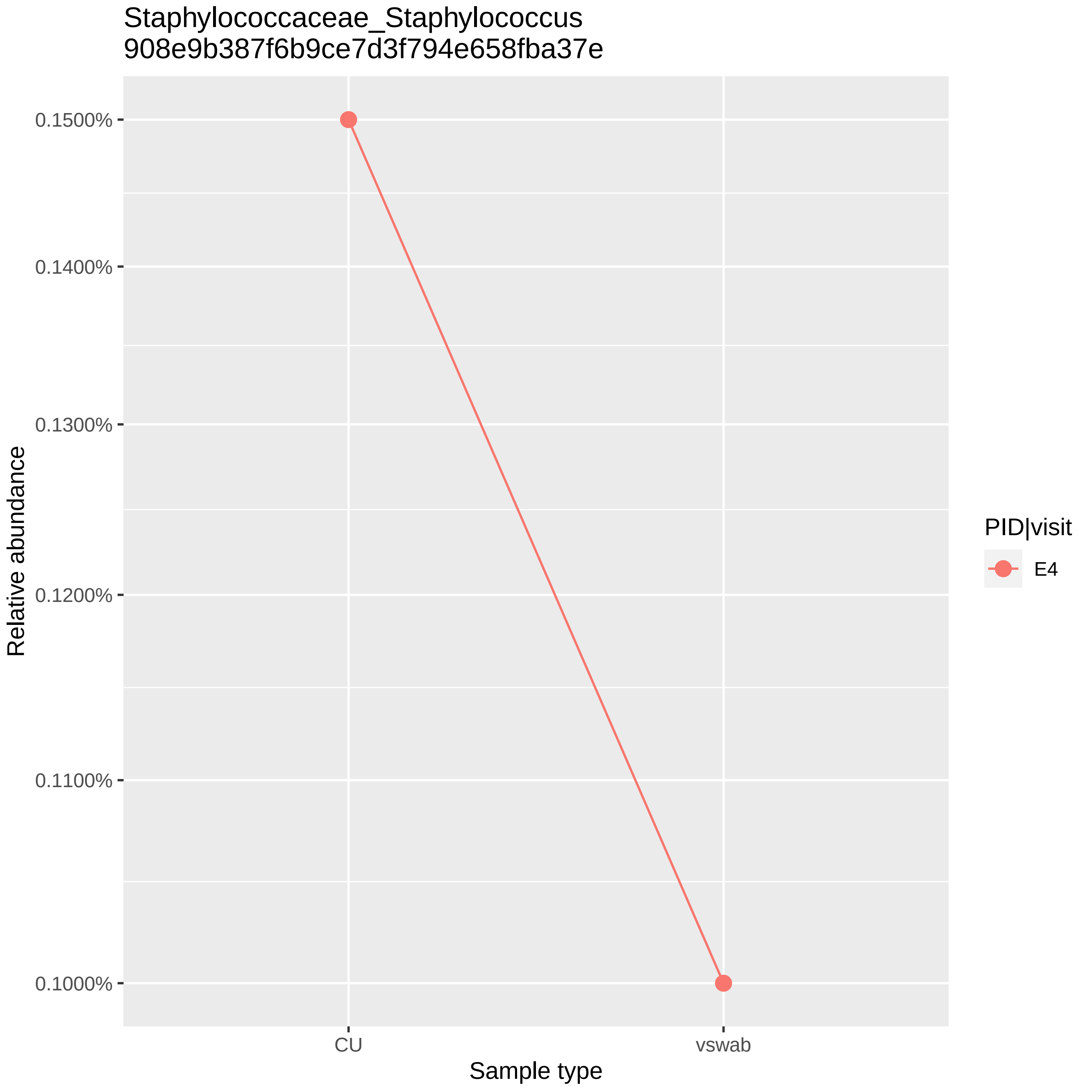
Staphylococcaceae_Staphylococcus
908e9b387f6b9ce7d3f794e658fba37e
0.1500%
0.1400%
0.1300%
0.1200%
0.1100%
0.1000%
Relative abundance
CU
vswab
Sample type
PID|visit
E4
