# Supplementary material for: Longitudinal variability in the urinary microbiota of healthy premenopausal women and the relation to neighboring microbial communities: A pilot study
Source: PLoS One. 2022 Jan 14;17(1):e0262095. doi: 10.1371/journal.pone.0262095 (PMC8759677; doi:10.1371/journal.pone.0262095)
Supplement: Attachment — Submitted filename: Response to Reviewers.pdf [file pone.0262095.s014.pdf]

Point-by-point reply to reviewer comments on manuscript

Title: Longitudinal variability in the urinary microbiota of healthy premenopausal women and the relation to neighboring microbial communities: a cohort study
ID: PONE-D-21-11667

Reviewer comments:

**Reviewer/ Comment**	**Reviewer comment**	**Reply by authors**	**Line in new manuscript**
Editor/1	Journal Requirements<br>We note that you have included the phrase "data not shown" in your manuscript. Unfortunately, this does not meet our data sharing requirements. PLOS does not permit references to inaccessible data. We require that authors provide all relevant data within the paper, Supporting Information files, or in an acceptable, public repository. Please add a citation to support this phrase or upload the data that corresponds with these findings to a stable repository (such as Figshare or Dryad) and provide and URLs, DOIs, or accession numbers that may be used to access these data. Or, if the data are not a core part of the research being presented in your study, we ask that you remove the phrase that refers to these data.	Yes, we have now included this data. See reply to Rev1/9 for details.	Supplementary S1 Fig
Reviewer1/1	Title<br>I would suggest changing the study description from "a cohort study" to "a pilot study" considering the low number of included participants.	We have changed the title accordingly.	p.1, Title
Rev1/2	Introduction<br>L. 72-73: please put in the reference.	Thank you for this remark, we have included the reference now.	P. 5, l. 69
Rev1/3	Methods<br>L. 148: please provide name of manufacturer.	The name of the manufacturer is mentioned in line 130. We prefer not to repeat this information.	No changes applied.

Reviewer/ Comment	Reviewer comment	Reply by authors	Line in new manuscript
Rev1/4	Methods<br>L. 152-160: Have replicate DNA extractions been made or only one extraction per participant? Use of replicates would strengthen the study, especially when analyzing samples with low DNA concentrations and sample numbers are low.	Unfortunately, we did not do replicate DNA extractions. In the light of these low-abundance samples, we totally agree that replicates would be of high value.<br>We added the following sentence to our discussion of limitations: *"In addition, we did not do extraction replicates that would have allowed us to confirm the microbiota compositions seen in these low-abundance samples."*	p. 29, L 606
Rev1/5	Results<br>L. 237-238: How did antibiotic treatment affect the results? And what was the time span from treatment to sample collection.	We agree that details on the antibiotic exposure and assessment of potential influence are important in any study on human microbiota. As background information, please find a short overview of the antibiotic exposure in our cohort:<br>- Volunteer C received six days amoxicillin, sample 4CU was taken 40 days after the last day of antibiotic treatment (ABx)<br>- Volunteer D received six days of ciprofloxacin, sample 3CU (=the third catheter urine sample) was taken 16 days after the last day of ABx, sample 4CU 63 days afterwards<br>- Volunteer E received seven days of amoxicillin, sample 4CU was taken 89 days afterwards<br>- Volunteer K developed a UTI and received a single day fosfomycin, the regularly planned sample 4CU was taken 35 days afterwards<br>While we deem this list as too detailled for the manuscript, we have now included information on the time span from treatment to sample collection both in the Results section on Sample Collection and in the Figure legend to Figure 1. Most samples were indeed taken quite long after antibiotic treatment.<br><br>Added in the figure legend:<br><br>*"Antibiotic exposure was reported 40 days prior sample C4CU, 16 and 63 days prior sample D3CU and D4CU, respectively, 89 days prior sample E4CU, and 35 days prior sample K4CU"*	Sample collection: p. 14, l. 248ff<br><br>Figure legend Fig1: p. 15 l. 286<br><br>Discussion, p. 29, l. 612ff.

Reviewer/ Comment	Reviewer comment	Reply by authors	Line in new manuscript
		Regarding the influence of antibiotic exposure in our cohort: When looking at the taxa bar plots in figure 1A, we do not see an obvious effect of antibiotic treatment.<br><br>However, we now mention in our discussion of limitations: "*Third, an unexpectedly high proportion of volunteers (4/15) received antibiotic treatment during the study, which possibly influenced their urinary microbiota. However, the time span between antibiotic exposure and urinary sampling was long for most samples, and taxa bar plots showed no clear effect on urinary microbiota compositions (C , D and E) except for a clear change seen in the one volunteer that developed a UTI (K). While the impact of antibiotic exposure on the urinary microbiota warrants further investigation, we do not feel like the treatments reported in our cohort significantly affected our study findings.*"	
Rev1/6	Results<br>L. 286-287: Were there any significant changes in alpha-diversity? It is very difficult to see anything from that figure.	Thank you for this comment. The revisions of this manuscript included a major modification of our main results since we now identified and removed sequences most likely stemming from contamination in our analysis (see Rev1/9). Figure S2 is now different and very obviously demonstrates a high variation in alpha diversity of urinary microbiota over the observation period. We do not feel that adding any statistical tests for significance is warranted to support the reported alpha diversity metrics.	No changes applied
Rev1/7	Results<br>L. 287-289: The authors write that the microbiota composition remained similar throughout the study for most volunteers. I disagree with this statement, since several of the participants show shifts in urotypes over the collection period. For instance, there are shifts between bacteroides urotypes and lactobacillus urotypes for participant A, B, I, J, K, L, M and O.	We agree that interpretation of microbiota dynamics as stable or non-stable is somewhat arbitrary. While after revision of our manuscript, the respective paragraphs in the results and discussion sections are now changed (see reply to Rev1/9), we still would like reply to the question of shifts between urotypes and explain why we did not refer to them.<br>While even for enterotypes, there is a controversial discussion on how a complex dynamic habitat such as the human gut microbiome can be stratified in a defined number of stable states (Costea Nat Microbiol 2018), we find the concept of urinotypes an interesting approach. We agree that it can be helpful to assess clustering into certain	No changes applied.

Reviewer/ Comment	Reviewer comment	Reply by authors	Line in new manuscript
		community types in microbiome studies in order to reduce complexity when assessing associations with health and disease. However, as seen in the non-concordance of urotype categorization between ECUQ and sequencing data in Price et. al (Price BJOG 2020), the reliability of these categories and their clinical relevance are yet to be established.<br>Instead of referring to urinotypes, we addressed the determination of stability/variability via Jensen-Shannon divergence metrics and beta diversity analyses and by contrasting the longitudinal variability of urinary microbiota with that in fecal samples. We feel that our approach complements previous analyses and hopefully leads to further research on the longitudinal variability of urinary microbiota.	
Rev1/8	Discussion<br>L. 407-409: Again, I disagree with the conclusion on a long-term stability of the urinary microbiota. The composition appears to shift a great deal.	Please see reply to Rev1/9 and the now revised Results and Discussion sections. We now observed even greater shifts in microbiota compositions and modified our interpretation.	See Rev1/9
Rev1/9	Discussion<br>L. 430-432: The authors note that, unlike others, they find a high proportion of urine samples with high relative abundances of Bacteroides. This is very puzzling, and I am concerned that cross-contamination – either during sampling or the following lab analyses, occurred. The authors claim that it is highly unlikely that Bacteroides was introduced as a laboratory contaminant and that the negative controls were instead contaminated by urine samples. I find this less likely. Instead the use of increased input volumes of extracted DNA, as described in the methods section, could increase contamination contribution from sampling or extraction procedures. Do they see an overlap between samples with higher input volumes and Bacteroides urotypes? Could the authors please	We sincerely have to thank you for thoroughly questioning this part of our former analyses and interpretation. Consequently, we reassessed our sequencing data and now agree on the interpretation of Baceroides as a major contaminant.<br>Since this step substantially changed our results and manuscript, we would like to explain, why we misinterpreted this part in the first place and how we now proceeded:<br>- Initially, we did not look at all negative controls thoroughly. We had included a couple of negative controls in a test run, the results of which were later omitted due to lower quality of reads (too short). At that point, we did not proceed with bioinformatics analysis of that test run since we decided to repeat it. Unfortunately, when repeating this run with the optimized protocol (the one reported in the manuscript and applied to included samples), we only repeated few of the negative controls.<br>- Please see figure 1 in this document below for the negative controls that we had from the later runs and that we took into	Abstract modified.<br><br>Methods: p. 8, l 135;<br>p. 11, l. 198<br><br>Results: all sequencing results modified; Supplementary S1 Fig added (showing control samples);<br><br>Discussion: results and interpretation modified, paragraph on contamination on p. 29, l. 597ff

Reviewer/ Comment	Reviewer comment	Reply by authors	Line in new manuscript
	provide sequencing data on the negative controls and further describe how the negative controls were constructed and handled? This is lacking in the methods section.<br>Furthermore, the authors speculate that the high degree of Bacteroides could be specific for premenopausal women. However, two studies (Price et al. 2019. The urobiome of continent adult women: a cross-sectional study and Amitzbøll et al., 2021. Pre- and postmenopausal women have different core urinary microbiota), have investigated the urinary microbiota (catheterized) of premenopausal women, and did not identify Bacteroides as a main contributor. The authors should include a discussion of their results against these two studies.	account before this revision. Only 2/9 control samples had a relevant detection of Bacteroides at that point.<br>- After receiving your comments, we went back to the results of this initial test run and noticed the much more frequent detection of Bacteroides with high abundances as seen in our study samples (see Supplementary Figure S1).<br>- In order to determine the reliability of this run, we compared the microbiota compositions of samples that were repeated, see Figure 2 in this document with highly similar results despite the shorter read length.<br>- We then assessed the correlation between the abundance of certain ASVs and the DNA concentration after extraction (see Karstens Nat Rev Urol 2018).<br>- Since no consensus for the criteria to remove sequences as contaminants exist, we applied the following: *"In order to eliminate sequences originating from contamination during sampling and procession steps, we filtered amplicon sequence variants (ASVs) with the following criteria: mean abundance in negative controls over 0.5%, presence of ASV in all negative sampling controls and relative abundance of ASV >5% in at least one negative sampling control. These ASVs were deleted from the analysis."*<br>- We identified 6 ASVs belonging to 4 genera as highly likely contaminants and removed their sequences from the complete dataset.<br>- Consequently, we repeated our bioinformatics analysis and corrected the results in our manuscript<br>- We now mention more details on how control samples were collected and handled<br>- We tried to identify the source of the contamination, but unfortunately the reagents and exact tubes were no more available. We repeated negative controls from the catheters and collection tubes, but did not find the identical sequences. Since the contaminating ASVs were also present in our PMA	

Reviewer/ Comment	Reviewer comment	Reply by authors	Line in new manuscript
		pretreated samples, we assume that contamination most likely originated from extraction reagents, but could not proof this.<br>- We decided to remove the part on PMA pretreatment, as this was initially planned as a way to exclude external contamination by sequences from non viable cells. Since this attempt was unsuccessful in our case and the initial results not quite consistent, we decided to repeat these experiments within future studies.<br><br>While we are aware that we clearly missed the opportunity to detect this contamination earlier, we also feel that reporting this and including a transparent discussion on this is of merit for future research on urinary microbiota. As shown in a recent study (Cumpanas Diagnostics 2020) only a small proportion of paper report how they the risk of contamination into account.	
Rev1/10	Discussion<br>L. 438: The authors have recorded the time of menstruation for their participants, but do not report on how this might affect their results. Does collection of samples always occur at the same time in the menstrual cycle for all women? Or does it vary for the individual participants and also between participants?	No, sampling did not always occur at the same time within the menstrual cycle of one individual. To clarify timing of sampling in relation to menstrual cycle, we added the information, that this was recorded for each sampling visit plus the following statement in the Method section:<br>*“Timing of sample visits was regardless of exact week in menstrual cycle, but sampling did not take place during menstruation.”*<br><br>Regarding the influence on the results: We already included week in menstrual cycle as a variable in the multivariate analysis in Table 2.	p. 8, l 119ff
Rev2/1	Major<br>-The biggest methodological limitation in this study is the high proportion of missed samples, patients with unexpected antibiotics, and infections. While this is unavoidable in a cohort study, it limits the conclusions that can be drawn	Thank you for this comment. Please see Rev 1/5 regarding the samples taken after antibiotic exposure.<br>However, we respectfully disagree that there is a high proportion of missed samples.<br>- Within the regular cohort, we planned 60 catheter urine (CU) samples and collected 58.	See changes listed under Rev1/5.<br><br>And p. 29, l. 593

Reviewer/ Comment	Reviewer comment	Reply by authors	Line in new manuscript
	from an already small sample size. This should be more clearly stated as a limitation.	- Within the follow-up cohort (visit 5, which was not initially planned) we collected 10/10 CU samples<br>- There is one additional CU sample after a UTI. Development of UTI was foreseen to occur in this cohort and collection in case of UTIs was already planned beforehand.<br>From our experience, this is quite a good adherence to sampling schedules for an observational study lasting more than six months per participant.<br><br>We now mention the two missed CU samples in the discussion of our limitations:<br>*"In two volunteers, one CU sampling each was missed."*	
Rev2/2	Major<br>-The authors should consider performing metagenomic analysis, even if only on a subset of samples, in future studies to assess strain-level taxonomic resolution and to definitively clarify whether strains in feces and urine overlap.	Thank you for this important suggestion. We are currently conducting follow-up studies and are planning to include metagenomic sequencing in order to assess strain overlap between reservoirs in more detail.<br>Since we already mention this suggestion for future research in our discussion, we did not apply any changes to the manuscript.	No changes applied.
Rev2/3	Intro<br>-Line 88 – this may be true for the female microbiome, but there is evidence in males (e.g. PMID 30143471). This should be cited or re-worded.	Thank you for bringing this to our attention. We now cite this article in the introduction and reworded the following sentence by including the word "female":<br>*"Since both reservoirs may influence urogenital disorders, it seems important to characterize previously described differences also in <u>female</u> asymptomatic volunteers."*	p. 6; l. 88 + l. 90
Rev2/4	Methods<br>As the authors acknowledge, the difference in sample processing methodology between samples of varying sources could add significant confounding effects on the comparison of data between them. Ideally, a standardized protocol should have been developed, but this is now beyond the scope of this manuscript.	Thank you, yes we agree that this is something to improve in future work.	No changes applied

Reviewer/ Comment	Reviewer comment	Reply by authors	Line in new manuscript
Rev2/5	Methods<br>Were women with inflammatory bowel disease included?	No, none of the participants had IBD though it was not an exclusion criterion in the first place. See Table 1 and table legend for details of underlying diseases of participants.	No changes applied.
Rev2/6	Methods<br>What taxa were identified in negative extraction and PCR controls? Were these subtracted out from specimens?	Thank you for this important question. We now show details of negative extraction and PCR controls, see reply to Rev1/9 and changes mentioned there.	See Rev1/9
Rev2/7	Methods<br>The "week of menstrual cycle" component of this study is an admirable attempt at capturing this, but the small number of samples per patient weakens this. I suspect that large differences occur over the short period of time when menstruation is dynamically changing and a variety of products are being utilized.	Yes, we agree. Of note, sampling did not take place during actual menstruation (see reply to Rev1/10), so we were rather collected this data with regards to hormonal influences on the microbiota than to influences of menstrual products in use. Nevertheless, we agree that this is something not well studied yet.<br><br>We now added the following sentence to our discussion of limitations *"In particular, to study changes related to the menstrual cycle, more frequent sampling within one cycle would have been necessary."*	p. 29, l. 596
Rev2/8	Methods<br>Was ethnicity captured in this data set?	No, unfortunately not. The large majority of volunteers was European-Caucasian.	No changes applied.
Rev2/9	Results<br>Clarify whether the patient with MS had any neurogenic bladder symptoms or workup	Thank you for this important remark. We added a short information on this in the table legend to Table 1: "*<u>Newly diagnosed</u> Multiple sclerosis <u>without any symptoms or signs of neurogenic bladder</u>*"	p. 13, l. 238
Rev2/10	Results<br>The high rate of infection and use of antibiotics by participants during this study is a potential limitation and should be stated as such.	Please see reply to Rev1/5 and Rev2/1. We now report more in detail, which time span there was between antibiotic exposure and sampling.	See changes applied to Rev1/5 and Rev2/1
Rev2/11	Results<br>Why were ~30% of EQUC isolates not seen on 16s? Is this a classification error? Is there better correlation at genus/family/order levels?	We did check for correlation on family level and in some cases, there is a better overlap. However, if you look at our table S3, only in 16% of samples, not all EQUC isolates were confirmed by 16S rRNA gene sequencing.<br>This proportion of incongruence is not higher than in previous studies (i.e. Price EJOC 2020: 26 of 115 EQUC positive samples were completely negative in 16S).	No changes applied

Reviewer/ Comment	Reviewer comment	Reply by authors	Line in new manuscript
Rev2/12	Results<br>Time since last intercourse is a known variable affecting the microbiome. The authors should expand upon this in the discussion as a key component of the study.	We have modified our discussion after the current revision of our results (see Rev 1/9). Furthermore, we now mention this finding in the conclusion paragraph of our discussion:<br>*"Despite the small sample size, we were able to analysis of all CU samplesconfirm suggested a potential impact of time since last sexual intercourse (R^2=0.09) on urinary microbiota compositions in both models with and without stratification to volunteer ID."*<br><br>In addition, we now mention this finding in our abstract.<br>*"After stratification by volunteer, time since last sexual intercourse was shown to be a factor significantly contributing to beta diversity (R^2=0.14, p=0.001)."*	Discussion, p. 26, l. 520ff<br><br>Abstract, p. 3, l. 42ff
Rev2/13	Discussion<br>More time could be spent discussing how an individual's microbiome can be so different than other healthy volunteers and yet stable and non-pathogenic. Does this imply a large degree of tolerability in the female GU microbiome? It certainly will make pathogenic characteristics more difficult to identify.	We definitely agree that this in an interesting question and worth elaboration, even now that our revised results show less stability in urinary microbiota.<br>However, we feel that the discussion of what is a "uropathobiont", a "urocommensal" and a clear "uropathogen" would go beyond the scope of this manuscript. It clearly is something needed to readdress during research on urinary microbiota and urogenital diseases. However, we have included the thought on the difficulties for association-studies in our abstract and discussion.<br><br>Sentence in abstract:<br>*"The higher intraindividual variability of urinary microbiota as compared to fecal microbiota will be a challenge for future studies investigating. Our findings on the longitudinal variability of urinary microbiota may facilitate the identification of alterations associationsed with urogenital diseases and aiming at identifying pathogenic microbiota signatures."*<br><br>Added in discussion:<br>*"This comparison is of particular interest for future research on the role of urinary microbiota in health and disease. While already many associations between specific alterations of the gut microbiota and*	No changes applied<br><br>Abstract p. 4, l. 57ff<br><br><br>Discussion p. 25, l. 516

Reviewer/ Comment	Reviewer comment	Reply by authors	Line in new manuscript
		disease have been well described, the higher intraindividual variability in healthy urinary microbiota likely complicates the establishment of similar associations for urinary microbiota."	
Rev2/14	Discussion<br>The PMA discussion section could be significantly shortened	After revision of our results (see Rev 1/9) we felt that this section does no more fit within this manuscript.	PMA section deleted from manuscript
Rev2/15	References<br>I would recommend that the authors update their literature search, as several urinary microbiome studies have been recently published that warrant consideration and/or discussion	Yes, thank you. We included some more recent publications and updated our references.	See changes in reference list
Rev2/16	Figures<br>The low resolution nature of the figures makes them illegible for review. This makes review of the manuscript very difficult.	Thank you for this feedback. We now increased the resolution more.	Fig 1 + 2

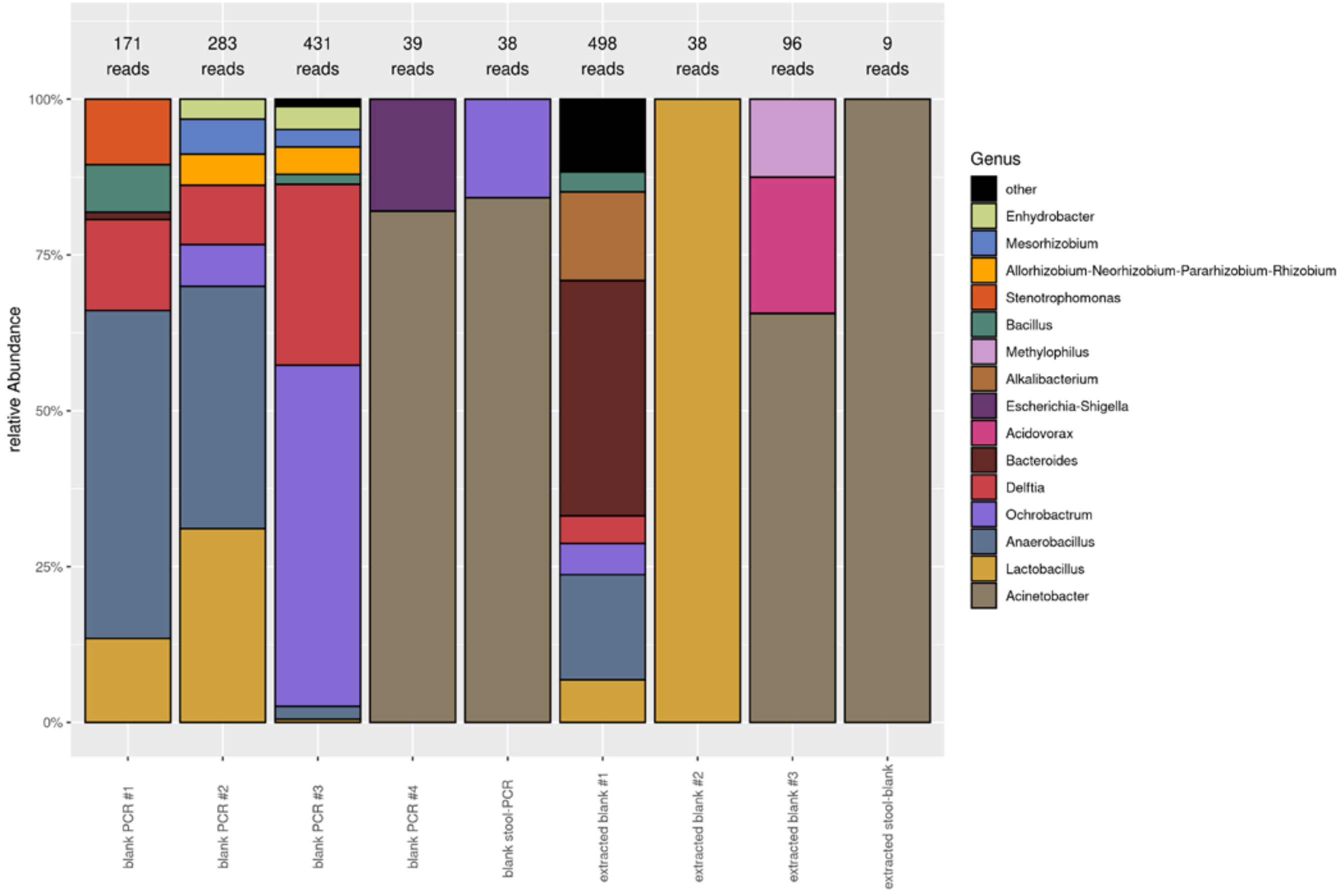

Figure 1: Relative abundance of bacterial genera in negative controls that were included in the later sequencing runs. Above the taxa bar plots the number of reads of the respective sample are indicated

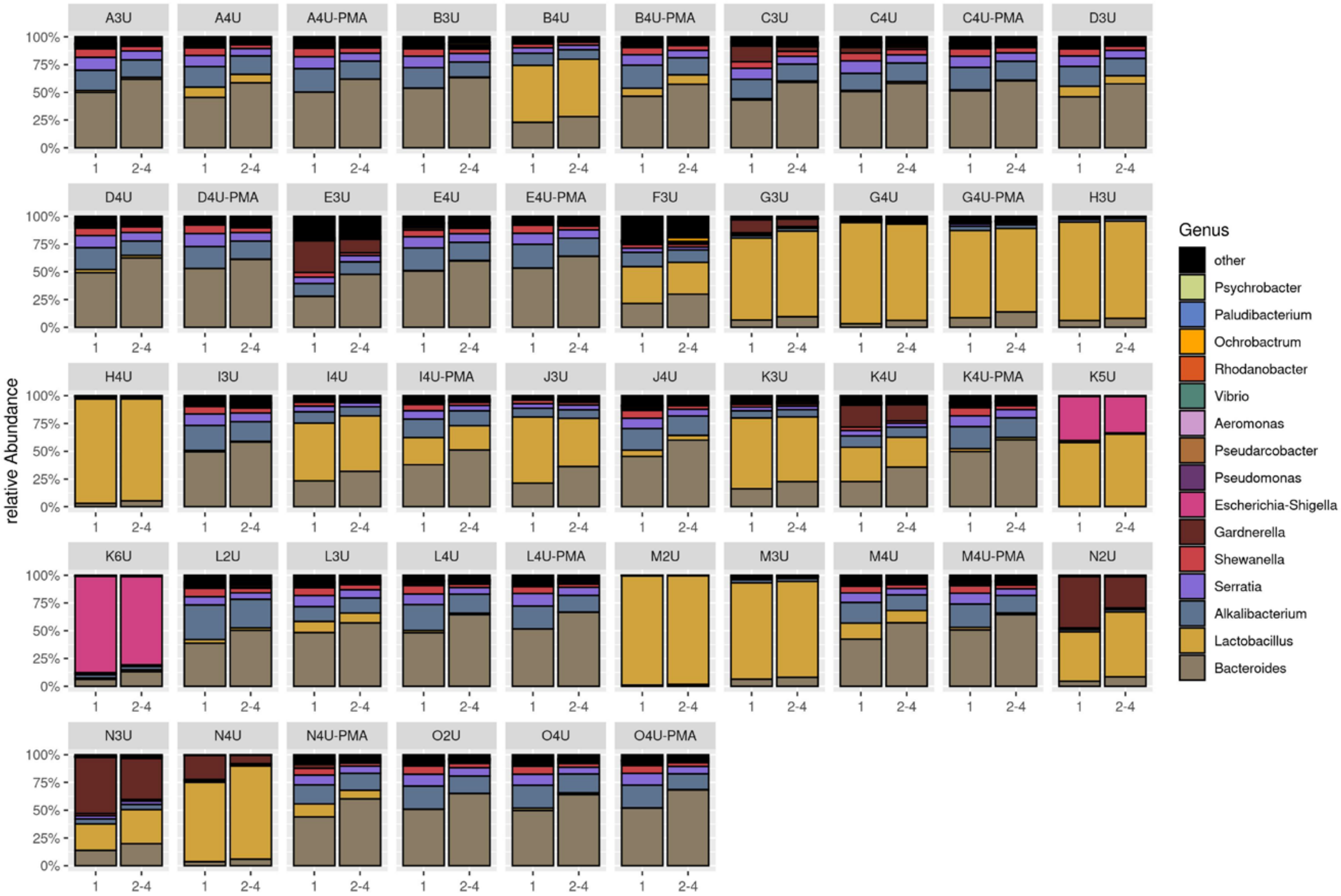

Figure 2: Relative abundance of bacterial genera in samples that were repeated after the initial test run (column 1)
