# Supplementary material for: Longitudinal variability in the urinary microbiota of healthy premenopausal women and the relation to neighboring microbial communities: A pilot study
Source: PLoS One. 2022 Jan 14;17(1):e0262095. doi: 10.1371/journal.pone.0262095 (PMC8759677; doi:10.1371/journal.pone.0262095)
Supplement: Attachment — Submitted filename: ResponsetoReviewer_PONE-D-21-11667R1.pdf [file pone.0262095.s015.pdf]

Point-by-point reply to reviewer comments on manuscript

Title: Longitudinal variability in the urinary microbiota of healthy premenopausal women and the relation to neighboring microbial communities: a pilot study
ID: PONE-D-21-11667R1

Reviewer comments:

**Reviewer/ Comment**	**Reviewer comment**	**Reply by authors**	**Line in new manuscript (version with track changes)**
Editor/1	Please explain/justify the use of the stepwise multivariate analysis. Also please explain which variables went into the model and how it was conducted in more detail.	Thank you for this comment. We have to apologize for this wording. We performed a multivariate analysis using PERMANOVA, but did not include all variables that were used in the univariate analysis due to strong interdependence. The variables diet, fluid intake and intake of contraceptive pill were nearly 100% consistent among samples of one volunteer.<br>We now clarified this step and omitted the term “stepwise”.	l. 312-314 and 325-327
Reviewer1/1	In line 315-318 you write that there are only few intra-individual chages in alpha-diversity (S2), and furthermore that the relative abundance of taxa remain similar throughout the study. I think that you have forgotten to update this section according to your new analyses.	Thank you for this remark. We changed the sentence.	l. 289ff
Reviewer2/1	The use of stepwise multivariate models is controversial and should be justified if not validated<br>Vaughan et al (PMID 34181466) should be cited	Please see our reply to Editor/1 regarding the use of the term „stepwise“. In addition, we now added the paper by Vaughan et. al to our discussion.	l. 451-452
Reviewer2/2	Line 168 – lysozyme is missing the “e”	Thank you, corrected.	l. 143
Reviewer2/3	Line 385 – unclear if the “fig” is supposed to be there	Thank you, deleted.	l. 388
Reviewer2/4	Line 400 – Visible is misspelled	Thank you, corrected.	l. 372
Reviewer2/5	Line 402 – peptoniphilus is misspelled	Thank you, corrected.	l. 375
