# Supplementary material for: Longitudinal variability in the urinary microbiota of healthy premenopausal women and the relation to neighboring microbial communities: A pilot study
Source: PLoS One. 2022 Jan 14;17(1):e0262095. doi: 10.1371/journal.pone.0262095 (PMC8759677; doi:10.1371/journal.pone.0262095)
Supplement: Attachment — Submitted filename: Revisions_Longitudinal variability in the urinary microbiota of healthy premenopausal women and the relation to neighboring microbial communities - a cohort study.docx [file pone.0262095.s013.docx]

Longitudinal variability in the urinary microbiota of healthy premenopausal women and the relation to neighboring microbial communities: a cohort study

Revisions

The authors have submitted a well written manuscript, describing an interesting study. Studies on the urinary microbiota are being published, but so far, they have mostly been of cross-sectional design. Longitudinal studies are highly warranted.

I do, however, have a few comments on the manuscript.

**Title**

I would suggest changing the study description from “a cohort study” to “a pilot study” considering the low number of included participants.

**Introduction**

L. 72-73: please put in the reference.

**Methods**

L. 148: please provide name of manufacturer.

L. 152-160: Have replicate DNA extractions been made or only one extraction per participant? Use of replicates would strengthen the study, especially when analyzing samples with low DNA concentrations and sample numbers are low.

**Results**

L. 237-238: How did antibiotic treatment affect the results? And what was the time span from treatment to sample collection.

L. 286-287: Were there any significant changes in alpha-diversity? It is very difficult to see anything from that figure.

L. 287-289: The authors write that the microbiota composition remained similar throughout the study for most volunteers. I disagree with this statement, since several of the participants show shifts in urotypes over the collection period. For instance, there are shifts between *bacteroides* urotypes and *lactobacillus* urotypes for participant A, B, I, J, K, L, M and O.

**Discussion**

L. 407-409: Again, I disagree with the conclusion on a long-term stability of the urinary microbiota. The composition appears to shift a great deal.

L. 430-432: The authors note that, unlike others, they find a high proportion of urine samples with high relative abundances of *Bacteroides.* This is very puzzling, and I am concerned that cross-contamination – either during sampling or the following lab analyses, occurred. The authors claim that it is highly unlikely that *Bacteroides* was introduced as a laboratory contaminant and that the negative controls were instead contaminated by urine samples. I find this less likely. Instead the use of increased input volumes of extracted DNA, as described in the methods section, could increase contamination contribution from sampling or extraction procedures. Do they see an overlap between samples with higher input volumes and *Bacteroides* urotypes? Could the authors please provide sequencing data on the negative controls and further describe how the negative controls were constructed and handled? This is lacking in the methods section.

Furthermore, the authors speculate that the high degree of *Bacteroides* could be specific for premenopausal women. However, two studies (Price et al. 2019. The urobiome of continent adult women: a cross-sectional study and Amitzbøll et al., 2021. Pre- and postmenopausal women have different core urinary microbiota), have investigated the urinary microbiota (catheterized) of premenopausal women, and did not identify *Bacteroides* as a main contributor. The authors should include a discussion of their results against these two studies.

L. 438: The authors have recorded the time of menstruation for their participants, but do not report on how this might affect their results. Does collection of samples always occur at the same time in the menstrual cycle for all women? Or does it vary for the individual participants and also between participants?
